# Incorporation of a Highly Reactive Oxalyl Thioester-Based Interacting Handle into Proteins

**DOI:** 10.1021/acs.orglett.3c01846

**Published:** 2023-06-29

**Authors:** Benjamin Grain, Rémi Desmet, Benoît Snella, Oleg Melnyk, Vangelis Agouridas

**Affiliations:** † Univ. Lille, CNRS, Inserm, CHU Lille, Institut Pasteur de Lille, U1019 - UMR 9017 - CIIL - Center for Infection and Immunity of Lille, F-59000 Lille, France; ‡ Centrale Lille, F-59000 Lille, France

## Abstract

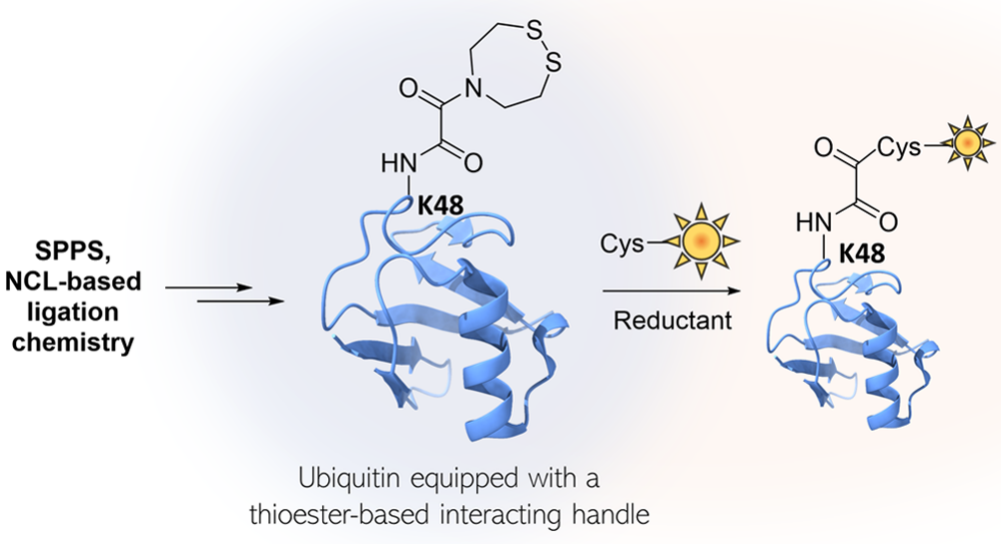

Providing biomolecules with extended physicochemical, biochemical, or biological properties is a contemporary challenge motivated by impactful benefits in life or materials sciences. In this study, we show that a latent and highly reactive oxalyl thioester precursor can be efficiently introduced as a pending functionality into a fully synthetic protein domain following a protection/late-stage deprotection strategy and can serve as an on-demand reactive handle. The approach is illustrated with the production of a 10 kDa ubiquitin Lys48 conjugate.

The reaction of a C-terminal peptide thioester with a cysteinyl peptide is a well-documented synthetic tool that enables the formation of a native peptide bond between two unprotected peptide segments under very mild pH and temperature conditions (Figure 1a).^1−3^ This reaction, referred to as native chemical ligation (NCL), has demonstrated its power for assembling functional protein domains of hundreds of amino acids (aa).^4,5^ In contrast, the use of NCL for protein site-selective modification lags a step well behind compared to other chemoselective chemistries such as the azide–alkyne Huisgen cycloaddition for several reasons.^6^ While C-terminal peptide or protein thioesters can be accessed with ease using biological,^7^ biochemical,^8^ or chemical^2^ methods, installation of the thioester functionality on internal protein sites is challenging. As a consequence, conjugation using NCL is mostly restricted to protein C or N extremities, and the works describing the site-selective introduction of thioesters at internal sites of proteins can be counted on the fingers of one hand.^9−11^ Moreover, the resort to NCL as a conjugation method is complicated by the modest reactivity of classical thioesters under dilute conditions and their moderate stability over long times in water.^12^ Thus, expanding the scope of NCL to the modification of internal protein sites requires new tools that enable one to overcome the aforementioned position and reactivity limitations, with the prerequisite of being applicable at the level of large protein domains.

**Figure 1:**
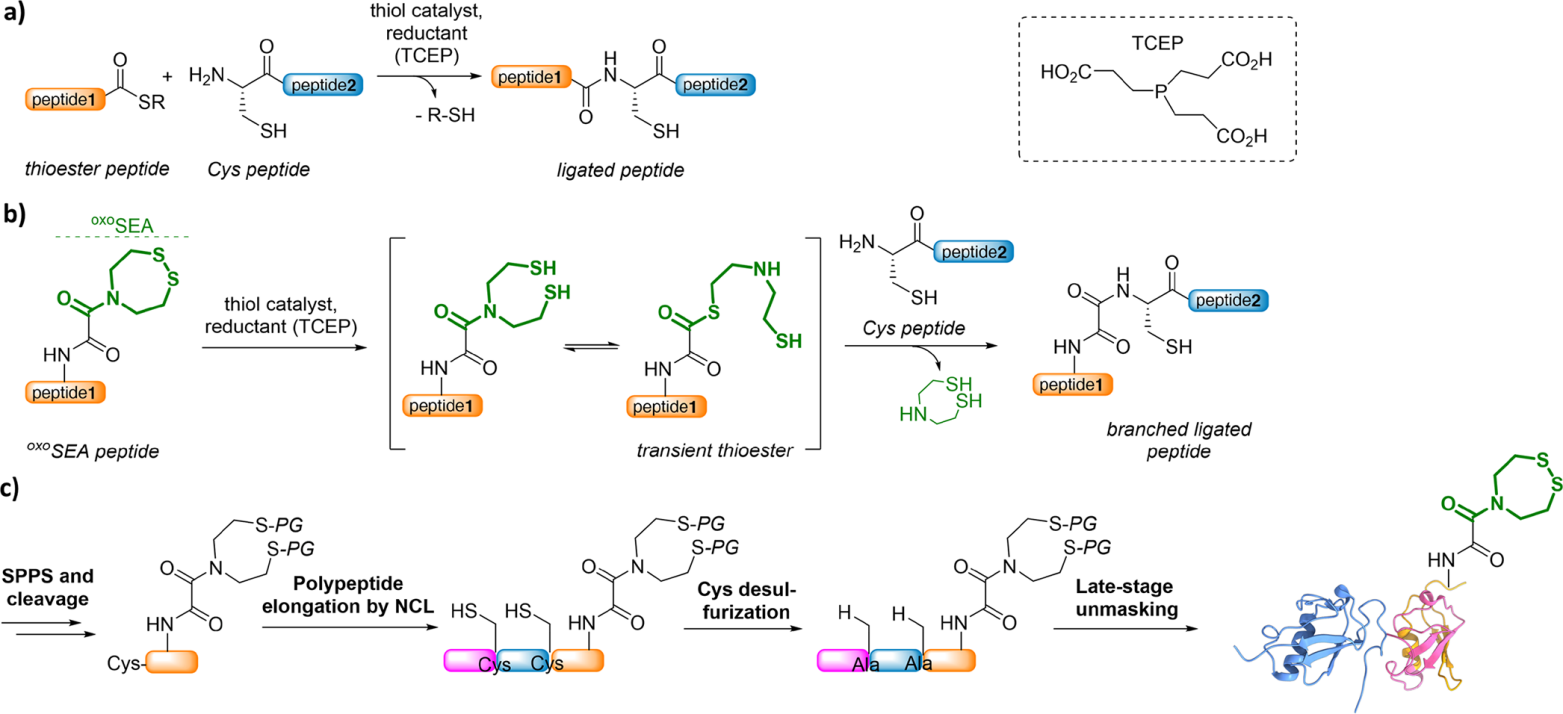
a) Principles of the NCL reaction. b) Principles of the ^oxo^SEA group-mediated ligation of peptides and proteins through the formation of a highly activated transient oxalyl thioester. c) Protecting group-based approach for the introduction of a pending ^oxo^SEA motif in polypeptides produced by NCL.

We recently addressed the reactivity problem by introducing an oxalic acid-derived thioester precursor, named the ^oxo^SEA group.^13^ This moiety can be grafted onto the side chain of a modified lysine residue and incorporated into peptide segments produced by solid phase peptide synthesis (SPPS) (Figure 1b). Masked in the form of an inert cyclic disulfide, its reactivity as an acyl donor is unveiled on demand upon exposure to disulfide reductive agents, such as *tris*(2-carboxyethyl)phosphine (TCEP). Under such conditions, the reduction of the disulfide bond triggers an intramolecular rearrangement that results in the formation of a highly activated transient oxalyl thioester capable of reacting with a Cys peptide. This reaction proceeds at rates of up to 30 M^–1^ s^–1^ and enables the formation of a branched conjugate in the high nanomolar concentration range. While the ^oxo^SEA handle displays attractive characteristics for protein modification, including in mixtures as complex as crude protein extracts of cell lysates, its incorporation is currently limited to small peptides produced by SPPS (<50 aa). Indeed, the ^oxo^SEA group cannot survive during the most popular reactions used for protein semisynthesis or total synthesis, namely, the NCL reaction itself and the desulfurization of cysteine residues into alanines,^14^ which takes place under reducing conditions. In this study, we disclose a general method for accessing proteins featuring an ^oxo^SEA motif at internal sites based on a protection/late-stage deprotection strategy (Figure 1c).

In such an approach, the selection of a convenient sulfhydryl protecting group (PG) is challenging because it is subject to many constraints. Indeed, the PG must survive the elongation steps during SPPS, including the trifluoroacetic acid (TFA)-mediated cleavage from the solid support, which must be resistant to polypeptide assembly through NCL and desulfurization reactions, and above all, its removal should occur without the intermediacy of free thiols, as doing so would inevitably trigger the premature activation of the ^oxo^SEA group. Based on these constraints, the 4-methoxybenzyl group (Mob) was identified as a potential thiol PG candidate for the ^oxo^SEA group for being readily installed from commercial reagents with reasonable synthetic effort and being quite resistant to concentrated TFA.^15,16^ However, we had to overcome the challenge of finding appropriate oxidative removal conditions since metal-assisted or oxidative cleavages of *S*-Mob groups are notoriously difficult.^17^

We started with the synthesis of the Fmoc-SPPS compatible lysine derivative **1**, which harbors a doubly Mob-protected ^oxo^SEA group on its side chain. According to the strategy described in Scheme 1, Mob groups were installed on amine **2** using 4-methoxybenzyl mercaptan. Acylation with ethyl oxalyl chloride and subsequent hydrolysis yielded acid **3** which was further coupled on the side chain of a Boc/O^*t*^Bu-protected lysine derivative to provide intermediate **4**. Installation of the Nα-Fmoc group was achieved in two steps to obtain final compound **1** on the gram scale, in six linear steps, and with an overall yield of 38%. Lysine derivative **1** was found to be stable during standard Fmoc-SPPS elongation procedures, and the stability of the Mob group toward TFA-mediated cleavage from the resin was assessed.

**Scheme 1:**
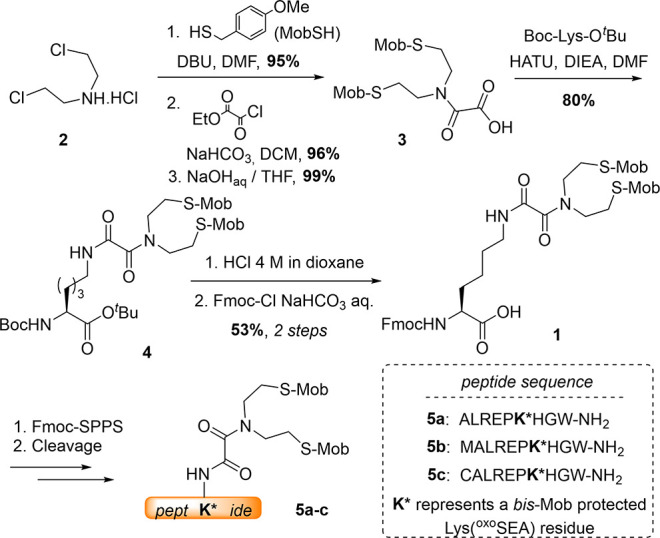
Synthesis of Mob-Protected Lysine Derivative **1** and Its Incorporation into Peptides

Mob thioethers have been reported to be stable in neat TFA at room temperature.^17^ However, *S*-Mob protecting groups can be removed by heating to 40 °C when scavengers are added to the cleavage cocktail.^18,19^ In our case, the cleavage of peptide **5a** from a Rink amide-functionalized solid support in a TFA/triisopropylsilane (TIS)/water/ethane-1,2-dithiol (90:5:2.5:2.5) mixture at 20 °C for 2 h 30 min led to the obtention of peptide **5a** (52% HPLC conversion), albeit with the formation of byproducts coming from the loss of one Mob group (30%) or two Mob groups (18%). Conversions were determined by UV detection at 215 nm, and the peak areas of peptides were corrected for the absorbance of the 4-methoxybenzyl moiety. While adapting the cocktail composition led to little change, decreasing the cleavage and deprotection time to 1 h at 20 °C enabled us to significantly modify the distribution of products in favor of peptide **5a** (69, 21, and 10% conversion). The same conditions applied to peptides containing methionine (Met) **5b** or Cys residue **5c** consistently resulted in similar conversion levels. With model peptides **5a**–**c** in hand, we next screened for deprotection conditions that would enable the generation of the ^oxo^SEA peptides directly in their cyclic form, **6a**–**c** (Scheme 2, Table 1). Note that Met- and Cys-containing peptides **5b**,**c** were used to investigate the influence of sulfur-containing moieties on the outcome of the reaction, as further discussed below.

**Scheme 2:**
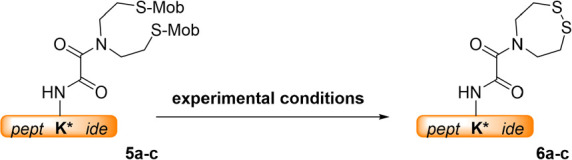
*S*-Mob Removal from Peptides **5a-c** to Provide ^oxo^SEA Peptides **6a-c**

**Table 1:** Optimization of the ^oxo^SEA Group Regeneration

Entry	Peptide	Experimental conditions	Yield for **6a**–**c** (%)^a^
1	**5a**	TFA/TIS (95:5 v:v), 2 h	6
2	**5a**	TFA/thioanisole (95:5 v:v), 4 h	54
3	**5a**	Ph_2_SO, MeSiCl_3_, TFA, 2 h	3
4	**5a**	Ph_2_SO, MeSiCl_3_, TFA/thioanisole (95:5 v:v), 2 h	75
5	**5a**	MeSiCl_3_, TFA/thioanisole (95:5 v:v), 2 h	65
6	**5a**	Ph_2_SO, MeSiCl_3_, TFA/thioanisole (95:5 v:v), 1 h	83, 34^b^
7	**5b**	Ph_2_SO, MeSiCl_3_, TFA/thioanisole (95:5 v:v), 4 h	73
8	**5c**	Ph_2_SO, MeSiCl_3_, TFA/thioanisole (95:5 v:v), 1 h	68

a HPLC conversions. Reactions were performed at a 1 mM peptide concentration and at 20 °C. When present, Ph_2_SO and MeSiCl_3_ were used at a respective concentration of 10 mM and 100 mM.

b Isolated yield (reaction performed on the preparative scale).

Using aqueous iodine as the oxidant proved unsuccessful. The ultimate goal of forming the ^oxo^SEA handle at the protein level led us to discard the other reported methods for the oxidative removal of *S*-Mob. Having observed a partial removal of Mob groups during the peptide deprotection and cleavage step, we were curious to examine the consequence of treating peptide **5a** with a TFA/TIS (95:5) mixture for prolonged reaction times (Table 1, entry 1).^19^ As expected, these conditions resulted in a poor conversion to **6a**. The desired product was accompanied by numerous byproducts, including substantial amounts of free-thiol intermediates. We then came across a recent report by Yang et al., who established that TIS can reduce Cys disulfide bonds formed on the solid phase during the cleavage and deprotection step in TFA.^20,21^ We therefore substituted hydrosilane with thioanisole. Interestingly, doing so resulted in the clean formation of the target ^oxo^SEA peptide **6a**, albeit with slow kinetics (Table 1, entry 2).

In the search for a more rapid deprotection system, our attention was attracted by the diphenylsulfoxide (Ph_2_SO)/trichloromethylsilane (MeSiCl_3_)/TFA cocktail used by Akaji et al. to cleave a large variety of Cys-derived thioethers.^22−24^ Unfortunately, incubating peptide **5a** in the presence of Ph_2_SO and MeSiCl_3_ in TFA at 20 °C resulted in a complex crude mixture of partially and fully deprotected products accompanied by side products coming from the alkylation of the peptide by unscavenged Mob carbocations (Table 1, entry 3). However, combining Akaji conditions with thioanisole as a scavenger resulted in the clean conversion of the starting material to the fully deprotected and cyclized ^oxo^SEA-containing peptide **6a** in less than 2 h (Table 1, entry 4 and Figure 2a). When the reaction was conducted in the absence of Ph_2_SO (Table 1, entry 5), lower conversions and accumulation of the monodeprotected intermediate were observed. Replacing thioanisole by other scavengers gave poor results as well (Supporting Information). This indicates that the reaction can likely proceed through activation by MeSiCl_3_ alone but that the presence of diphenylsulfoxide and thioanisole is important to promoting Mob oxidative cleavage (Figure 2b). We propose that, in addition to the known silyl chloride-sulfoxide pathway,^23^ thioanisole may be involved in a competitive pathway for ^oxo^SEA formation through thioether activation by Mob carbocations.^25^ The fact that thioanisole used alone in TFA permits the reaction (Table 1, entry 2) substantiates such a hypothesis. Moreover, thioanisole is expected to participate in fast and reversible sulfide–thiosulfonium interchanges with reactive intermediates *I* and *II*(26) and therefore to enable cross-talk between the two pathways, leading to the formation of ^oxo^SEA disulfide.

**Figure 2:**
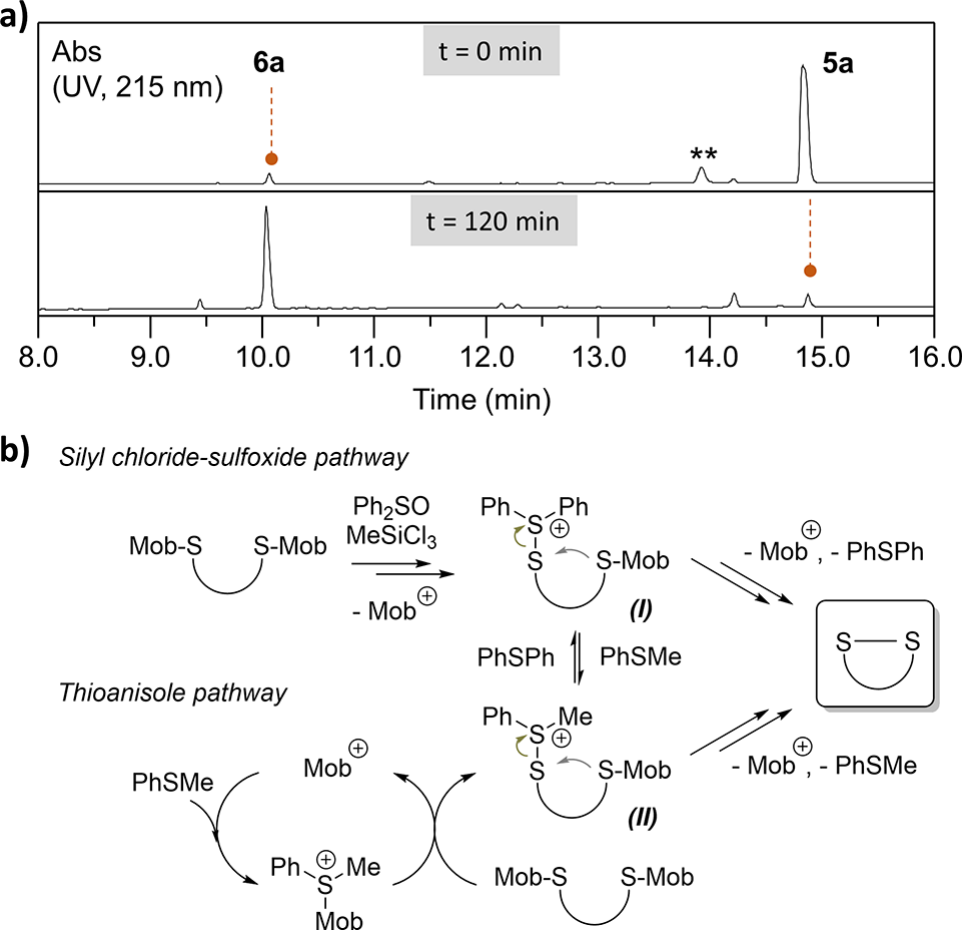
a) HPLC chromatogram of Mob removal on peptide **5a** under the conditions described in Table 1, entry 4 at 0 and 120 min (**: nonpeptidic material; Ph_2_SO removed by diethyl ether extraction before injection). b) Proposed mechanism for Mob deprotection in the presence of Me_3_SiCl and Ph_2_SO in TFA/thioanisole.

The reaction was repeated on the multimilligram scale and enabled the isolation of the expected product **6a** with 34% yield after RP-HPLC purification (Table 1, entry 6). When the experiment was performed on the preparative scale, we evidenced a scale dependency of the reaction rate, as the maximal conversion was reached in half the time that was observed for analytical samples. Although its origin remains to be established, this scale effect was found to be reproducible.

We also investigated whether a Met side chain thioether group would survive under the new conditions (Table 1, entry 7). When peptide **5b** underwent Mob oxidative deprotection, no product corresponding to the demethylation or oxidation of the Met residue was detected in the chromatogram, although the reaction was slower. Peptide **6b** was indeed obtained as the major product (73% HPLC conversion after 4 h). Finally, we were curious to investigate the impact of the presence of a nearby free thiol group by studying the Mob deprotection of peptide **5c** having a Cys residue in the vicinity of the Mob-protected ^oxo^SEA group (Table 1, entry 8). The expected peptide **6c** was again obtained as the major species (68% HPLC conversion after 1 h).

Importantly, tryptophan (Trp) residues were reported by Akaji et al. to be highly susceptible to the silyl chloride-sulfoxide treatment, even in the presence of anisole or 3-methylindole used as scavengers.^23^ The application of the deprotection protocol thus required the mandatory use of an *N*-formyl-protected indole side chain derivative. In our case, when unprotected Trp-containing peptides **5a**–**c** were treated in the presence of thioanisole used as a scavenger (Table 1, entry 4), no byproducts stemming from Trp alteration were observed. As such, these results extend the application of the MeSiCl_3_/Ph_2_SO/thioanisole system to unprotected Trp-containing peptides.

The developed conditions demonstrated satisfactory effectiveness and sufficient selectivity to be applied at the level of the synthetic protein domain (Figure 3a). In order to ensure that the Mob protection would survive the whole assembly sequence, we engaged model thioester precursor **7a** and the protected ^oxo^SEA-containing peptide **5c** in an NCL reaction.^27,28^ Polypeptide **9a** was isolated in 75% yield. It is worth noting that using a 3-mercaptopropionic acid-derived peptide thioester instead of SEA peptide **7a** resulted in the exact same outcome, showing that different peptide acyl donors can be used for the NCL step (Supporting Information). Subsequently, the ligated peptide **9a** underwent a metal-free desulfurization reaction to provide compound **10a** in 52% isolated yield. Finally, the ^oxo^SEA motif was generated using the conditions described earlier to form ^oxo^SEA peptide **11a** in 15% yield after RP-HPLC purification. Its functionality as an acyl donor in NCL was verified through ligation with Cys peptide **12**. The ligation rate observed during the reaction was consistent with that reported in previous work (Supporting Information).^13^

**Figure 3:**
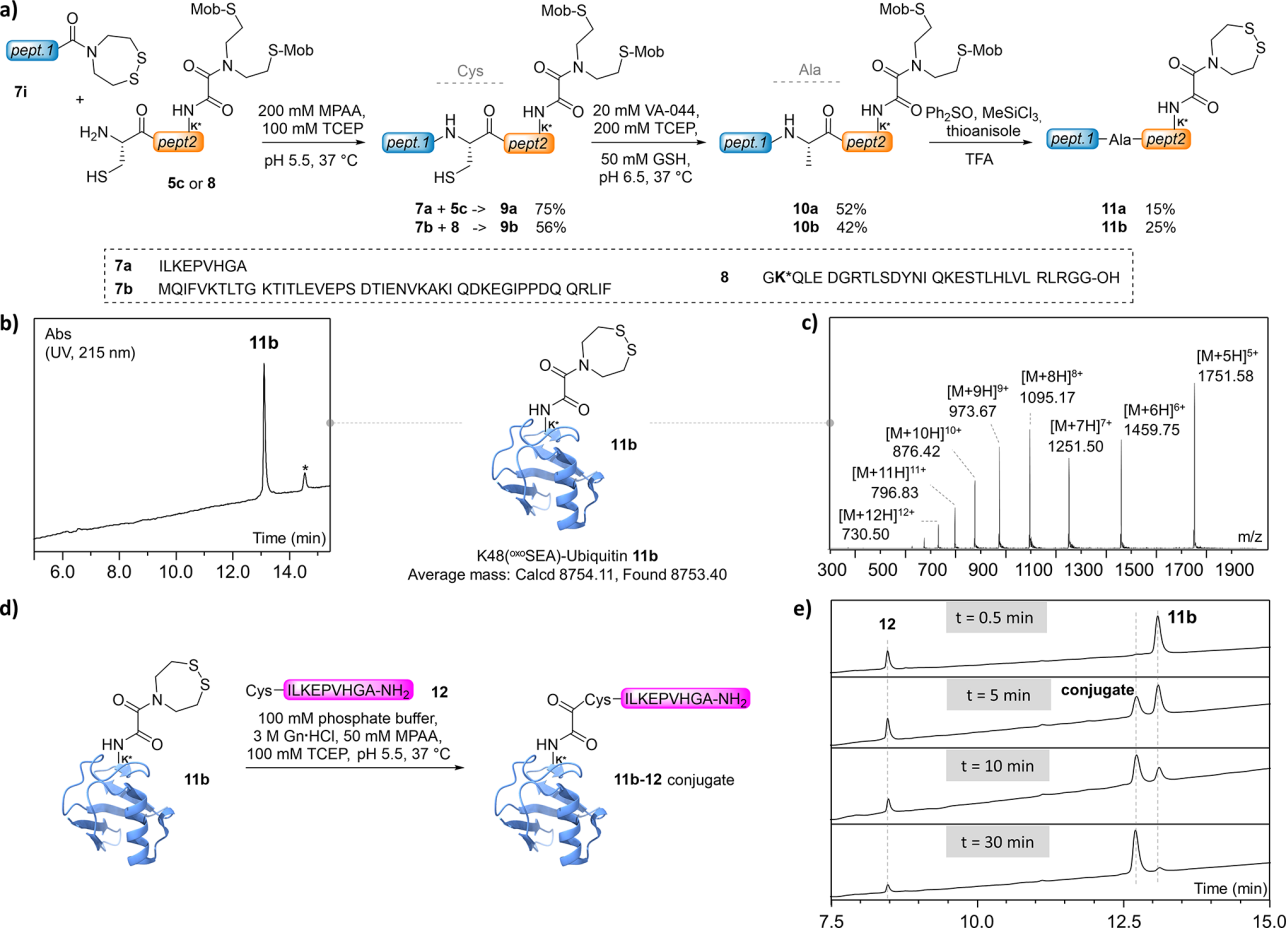
a) Synthesis of ^oxo^SEA-containing polypeptides produced by means of NCL and a desulfurization reaction. b) HPLC trace of purified **11b** (*: nonpeptidic contaminant). c) MS trace of the purified synthetic K48-modified ubiquitin **11b**. d) ^oxo^SEA-mediated conjugation of K48-functionalized ubiquitin with Cys peptide **12** (50 μM **11b** in 0.1 M phosphate buffer, 3 M guanidinium chloride, 1.5 equiv of **12**, 100 mM TCEP, 50 mM MPAA, pH 5.5, 37 °C). e) Kinetic monitoring of the formation of the Ub K48 conjugate **11b-12** (UV detection at 215 nm).

We next examined if the method would be suitable for the chemical synthesis of an ^oxo^SEA-functionalized protein, choosing the ubiquitin (Ub) protein **11b** carrying an ^oxo^SEA moiety on the side chain of the lysine 48 (K48) residue as a model. The assembly of **11b** started with the ligation of peptides **7b** and **8** to afford polypeptide **9b**.

The ligated product was readily desulfurized into peptide **10b** in 42% isolated yield and exposed to the Mob-deprotection cocktail. K48(^oxo^SEA)Ub **11b** was obtained in 25% yield after RP-HPLC purification and characterized by UPLC-MS (Figure 3b,c).

At this stage, it should be noted that the isolation of modified ubiquitin **11b** proved challenging due to solubility issues and to the erratic adsorption of the protein to glass and plastics. Indeed, neutralizing the positive charge of the K48 residue upon substitution by an ^oxo^SEA-functionalized lysine might affect the surface charge properties of ubiquitin by extending the hydrophobic patch centered on isoleucine 44. Such a dramatic change in hydrophobicity has been put forward to explain the changes in recognition and functions of ubiquitin upon K48 post-translational acetylation.^29^ Among the various protocols tested, direct injection of the crude reaction mixture in TFA on the HPLC column after dilution with phosphate buffer and diethyl ether extraction proved to be the most effective way to recover the targeted protein. In fact, maintaining the polypeptide in a strongly denaturing environment before purification probably prevented its aggregation and limited material loss through adsorption to the containers’ surfaces. Purified synthetic ubiquitin **11b** was then engaged in a site-selective conjugation with Cys peptide **12** in order to validate the incorporation of the ^oxo^SEA ubiquitin derivative. Considering the solubility issues encountered so far, the ^oxo^SEA-mediated ligation reaction was conducted with 50 μM **11b** and 1.5 equiv of peptide **12** in 3 M guanidinium chloride (Figure 3d,e). Under such conditions, the reaction was nevertheless complete in about 30 min and provided a conjugate which was fully characterized after Cys alkylation and trypsic digestion (Supporting Information).

In summary, we successfully introduced a highly reactive latent thioester precursor as a functional handle in a synthetic protein domain produced by means of thioester-based ligation reactions. To this end, we set up a protection/late-stage deprotection strategy utilizing a novel MeSiCl_3_/Ph_2_SO/thioanisole/TFA combination for removing *S*-Mob groups while generating the key ^oxo^SEA moiety. One important finding is that the addition of thioanisole as a scavenger enables clean Mob deprotection that can be applied to unprotected Trp-containing peptides. We have shown that the handle can be activated on demand and can be functional by producing a 10 kDa K48 conjugate of ubiquitin. The reactivity of oxalyl thioesters is particularly suited for conjugation at high dilution and can prove to be an interesting way to modify proteins having poor to moderate water solubility. As in the case of ubiquitin, however, the site of introduction of such a modification is to be considered with great care as it may modify the physicochemical properties of the protein and, consequently, its function. Beyond classical cross-linking approaches, ^oxo^SEA-based chemistry could also offer opportunities to access objects such as high-molecular-weight biopolymers through the mild polymerization of functionalized polypeptides or protein domains.

## Data Availability

The data that support the findings of this study are available in the present article and in the Supporting Information.

## Supporting Information Available

Experimental procedures and characterization of all compounds (PDF)
