# Supplementary material for: Incorporation of a Highly Reactive Oxalyl Thioester-Based Interacting Handle into Proteins
Source: Org Lett. 2023 Jun 29;25(27):5117–22. doi: 10.1021/acs.orglett.3c01846 (PMC10353032; doi:10.1021/acs.orglett.3c01846)
Supplement: Supplementary file 1 — ol3c01846_si_001.pdf [file ol3c01846_si_001.pdf]

Supplementary Information for

Incorporation of a highly reactive oxalyl thioester-based interacting handle into proteins

Benjamin Grain, Rémi Desmet, Benoît Snella, Oleg Melnyk,* Vangelis Agouridas[†]*

Univ. Lille, CNRS, Inserm, CHU Lille, Institut Pasteur de Lille, U1019 - UMR 9017 - CIIL - Center for Infection and Immunity of Lille, F-59000 Lille, France

[†] Centrale Lille, F-59000 Lille, France

* Corresponding author
ORCID 0000-0003-0911-1527
vangelis.agouridas@ibl.cnrs.fr

Table of content

1. General methods 3
Reagents and solvents 3
Peptide synthesis 4
Analyses 4
Purifications 5
2. Synthesis of Fmoc-Lys(^{oxo}SEA-Mob)-OH 1 6
General synthetic scheme 6
Protocols 7
3. Synthesis and characterization of model peptides 20
Amide peptides 20
Thioester peptides 27
4. Resistance of Mob groups during peptide cleavage from solid support after SPPS 29
5. Optimization of Mob cleavage conditions 31
6. Suitability for Cys- and Met-containing peptides 39
7. Synthesis and characterization of ^{oxo}SEA-containing polypeptide 43
Synthesis 43
^{oxo}SEA reactivity validation 53
8. Synthesis and characterization of Ubiquitin(^{oxo}SEA) protein 55
General synthetic scheme 55
Protocols 56
Functional characterization 67
9. References 75

1. General methods

Reagents and solvents

N-[(dimethylamino)1-1*H*-1,2,3-triazolo[4,5-*b*]-pyridin-1-ylmethylene]-N-methylmethanaminium hexafluorophosphate N-oxide (HATU), O-(1*H*-6-chlorobenzotriazole-1-yl)-1,1,3,3-tetramethyluronium hexafluorophosphate (HCTU) and *N*-Fmoc protected amino acids were obtained from Iris Biotech GmbH. Side-chain protecting groups used for the amino acids were Fmoc-Arg(Pbf)-OH, Fmoc-Asn(Trt)-OH, Fmoc-Asp(O^{t}Bu)-OH, Fmoc-Cys(Trt)-OH, Fmoc-Gln(Trt)-OH, Fmoc-Glu(O^{t}Bu)-OH, Fmoc-His(Trt)-OH, Fmoc-Lys(Boc)-OH, Fmoc-Ser(^{t}Bu)-OH, Fmoc-Thr(^{t}Bu)-OH, Fmoc-Trp(Boc)-OH and Fmoc-Tyr(^{t}Bu)-OH. Pseudoproline derivatives employed when required were Fmoc-Ile-Thr[Ψ(Me,Me)Pro]-OH and Fmoc-Leu-Thr[Ψ(Me,Me)Pro]-OH.

The synthesis of *bis*(2-sulfanylethyl)aminotrityl polystyrene (SEA PS) solid support was carried out as described elsewhere.[1,2] Piperidine, diisopropylcarbodiimide (DIC), 4-mercaptophenylacetic acid (97%, MPAA), 3-mercaptopropionic acid (MPA), *tris*(2-carboxyethyl)phosphine hydrochloride (≥ 98%), TCEP), triisopropylsilane (TIS), ethanedithiol (EDT), thioanisole, thiophenol, guanidine hydrochloride (Gn·HCl, ≥ 99%), sodium phosphate dibasic dihydrate (≥ 99%), hydrochloric acid (reagent grade, 37% w/v) and sodium hydroxide (pellets, 97%) were purchased from Sigma-Aldrich. All other reagents were purchased from Acros Organics or Merck and were of the purest grade available.

Peptide synthesis grade *N,N*-dimethylformamide (DMF), dichloromethane (CH_2Cl_2), diethylether (Et_2O), acetonitrile (CH_3CN), heptane, LC–MS-grade acetonitrile (CH_3CN, 0.1% TFA), LC–MS-grade water (H_2O, 0.1% TFA), *N,N*-diisopropylethylamine (DIEA), acetic anhydride (Ac_2O) were purchased from Biosolve and Fisher-Chemical. Trifluoroacetic acid (TFA) was obtained from Biosolve. Water was purified with a Milli-Q Ultra Pure Water Purification System.

---

[1] Ollivier, N.; Dheur, J.; Mhidia, R.; Blanpain, A.; Melnyk, O. *Bis*(2-sulfanylethyl)amino Native Peptide Ligation. *Org. Lett.* **2010**, *12*, 5238-5241.
[2] Ollivier, N.; Raibaut, L.; Blanpain, A.; Desmet, R.; Dheur, J.; Mhidia, R.; Boll, E.; Drobecq, H.; Pira, S. L.; Melnyk, O. Tidbits for the synthesis of *bis*(2-sulfanylethyl)amido (SEA) polystyrene resin, SEA peptides and peptide thioesters. *J. Pept. Sci.* **2014**, *20*, 92-97.

Peptide synthesis

Peptides were synthesized using standard Fmoc solid phase peptide synthesis methods. Peptide amides were prepared on a NovaSyn TGR solid support (0.25 mmol g^{-1}), peptide acids were prepared on a Wang solid support (0.82 mmol g^{-1}) and peptide thioesters were prepared on a SEA PS solid support (0.16 mmol g^{-1}).

Unless otherwise stated, peptide elongation was performed at room temperature (rt) using an automated peptide synthesizer. In brief, amino acids (10 equiv) were activated using HCTU (9.5 equiv)/DIEA (20 equiv) in DMF. The peptidyl solid support was acetylated after each coupling step using Ac_2O/DIEA/DMF 10/5/85 v/v/v. The removal of the Fmoc group was performed by treating the peptidyl solid support with DMF/piperidine 80/20 v/v. After elongation, the peptidyl solid support was washed with DMF (3 × 1 min), DCM (3 × 1 min) and Et_2O (2 × 1 min). The solid support was finally dried in vacuo. The experimental conditions used for the final cleavage and deprotection step are indicated for each peptide.

Analyses

^{1}H and ^{13}C NMR spectra were recorded on a Bruker Advance-300 spectrometer operating at 300 MHz and 75 MHz respectively. The spectra are reported as parts per million (ppm) down field shift using tetramethylsilane as internal reference. The data are reported as chemical shift (δ), multiplicity, relative integral, coupling constant (J Hz) and assignment where possible.

Analytical HPLC as well as micro preparative HPLC were performed with a Thermofischer system on a reverse phase column XBridge BEH300 C18 (3.5 µm, 300 Å, 4.6 × 150 mm) using a linear gradient of increasing concentration of eluent B in eluent A (eluent A: 0.1% by vol. of TFA in water; eluent B: 0.1% by vol. of TFA in acetonitrile). The column eluant were monitored by UV at 215 nm or 280 nm.

Purified products as well as reaction mixtures were characterized by analytical UPLC–MS using a System Ultimate 3000 UPLC (Thermofisher) equipped with a column Acquity peptide BEH300 C18 (1.7 µm, 2.1 × 100 mm), a diode array detector, a charged aerosol detector (CAD) and a mass spectrometer (Ion trap LCQfleet). Analyses were performed at 50 °C using a linear gradient of 0-50% of eluent B in eluent A over 15 min at a flow rate of 0.4 mL min^{-1}. The column eluate was monitored by UV at 215 nm or 280 nm and CAD. The peptide masses were measured by on-line UPLC–MS (LCQ Fleet Ion Trap Mass Spectrometer,

ThermoFisherScientific): heat temperature 350 °C, spray voltage 2.8 kV, capillary temperature 350 °C, capillary voltage 10 V, tube lens voltage 75 V.

HRMS analyses were performed on a Thermo Scientific Orbitrap Mass Spectrometer Exactive equipped with Heated Electrospray Ionisation Source (HESI-II) / Ultra High Resolution (100000). Instrument control, data acquisition and processing were performed using the associated XCalibur 2.2 and Exactive 1.1 softwares.

The IR spectra were recorded on a Bruker FT-IR spectrometer ALPHA.

The Fmoc-Lys(^{oxo}SEA-Mob)-OH amino acid **1** was prepared using classical instrumentation and methods used in synthetic organic chemistry. Structural characterization and purity were determined by ^{1}H and ^{13}C NMR, MS, HRMS, IR and polarimetric analyses.

Purifications

Preparative reverse phase HPLC of crude peptides were performed with a preparative HPLC Waters system using a reverse phase XBridge BEH300 Prep C18 column (5 µm, 300 Å, 10 × 250 mm or 19 × 150 mm) and appropriate linear gradient of increasing concentration of eluent B in eluent A (flow rate of 6 mL min^{-1} or 20 mL min^{-1}, detection at 215 nm). Selected fractions were then combined and lyophilized.

2. Synthesis of Fmoc-Lys(oxoSEA-Mob)-OH 1

General synthetic scheme

Fmoc-Lys(oxoSEA-Mob)-OH amino acid **1** was synthesized in 6 steps from commercially available *bis*(2-chloroethyl)amine hydrochloride **2** as described in Figure S1. Boc-Lys-O^{t}Bu derivative **15** was synthesized as described elsewhere.[3] In brief, Mob-protected oxoSEA synthon **13** was obtained after nucleophilic substitution of Mob mercaptan on starting material **2** followed by ethyl chlorooxoacetate acylation and saponification. Coupling of compound **3** to the side chain of lysine derivative **15** was achieved using classical activation protocols to provide **4**. The Boc carbamate and the O^{t}Bu ester were removed at once upon exposure to HCl in dioxane and the resulting free amine function was reprotected as a Fmoc group in a *one-pot* fashion, allowing to yield targeted amino acid **1**.

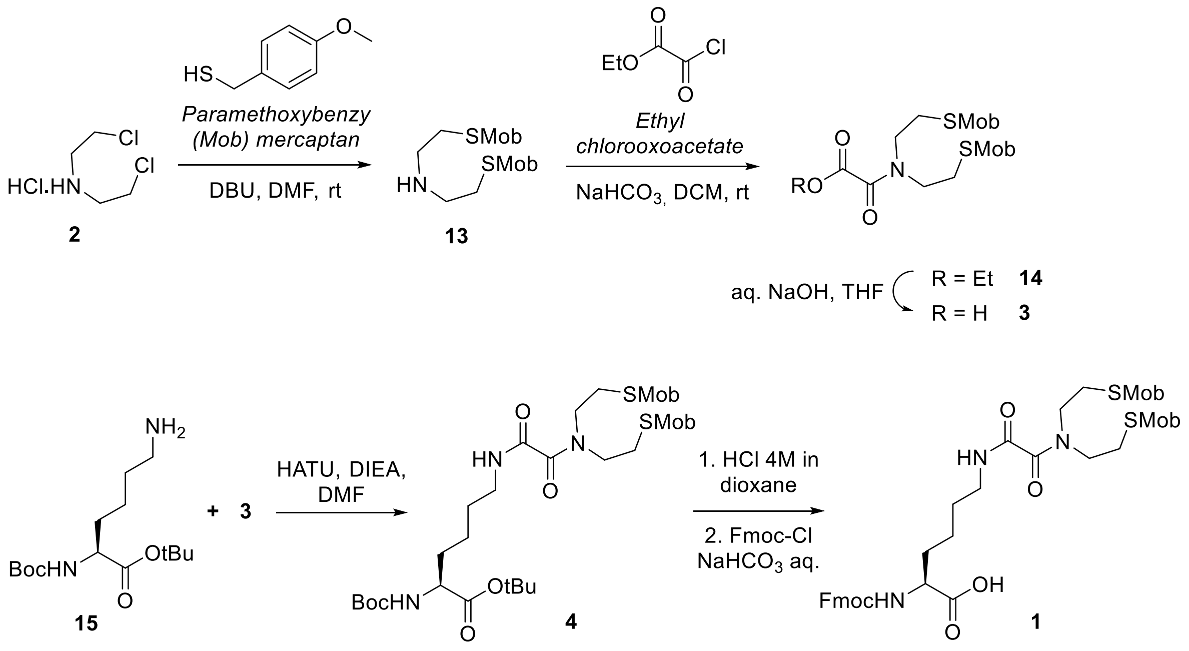

Figure S1. General synthetic scheme for the preparation of Fmoc-Lys(oxoSEA-Mob)-OH **1** in 6 steps from commercially available *bis*(2-chloroethyl)amine **2**.

---

[3] Snella, B.; Grain, B.; Vicogne, J.; Capet, F.; Wiltschi, B.; Melnyk, O.; Agouridas, V. Fast Protein Modification in the Nanomolar Concentration Range Using an Oxalyl Amide as Latent Thioester. *Angew. Chem., Int. Ed.* **2022**, *61*, e202204992.

Protocols

Bis[2-((4-methoxybenzyl)sulfenyl)ethyl]amine **13**

To a solution of *bis*(2-chloroethyl)amine **2** (1.78 g, 10 mmol) in DMF (30 mL) is added *p*-methoxybenzyl mercaptan (3.47 mL, 25 mmol). The resulting suspension is cooled to 0 °C under argon and 1,8-diazabicyclo[5,4,0]undec-7-ene (7.46 mL, 50 mmol) is added dropwise. The ice bath is removed and the reaction is left to proceed at room temperature during 24 hours. The solvent is evaporated under reduced pressure. Crude product is dissolved in DCM (60 mL) and washed with a 5% aqueous solution of KH_2PO_4 (3 × 60 mL). The organic layer is dried over $MgSO_4$ and concentrated to dryness under reduced pressure. The crude mixture is purified by column chromatography (Eluent: Cyclohexane/Ethyl acetate/Triethylamine from 70/30/0.1 to 60/40/0.1). Product **13** was isolated as a yellow oil (3.58 g, 95%).

^{1}H NMR (300 MHz, $CDCl_3$, Figure S2) δ 7.27 (m, *J* = 8.7 Hz, 4H), 6.88 (m, *J* = 8.7 Hz, 4H), 3.81 (s, 6H), 3.70 (s, 4H), 2.74 (t, *J* = 6.6 Hz, 4H), 2.58 (t, *J* = 6.3 Hz, 4H), 1.78 (s, 1H) ppm. **^{13}C NMR** (75 MHz, $CDCl_3$, Figure S3) δ 158.6, 130.3, 129.9, 113.9, 55.3, 47.9, 35.5, 31.5 ppm. **IR** (ATR, cm^{-1}) 2906, 2883, 1609, 1509, 1283, 1173, 1032. **MS** (positive detection mode, Figure S4) $[M+H]^+$ calcd. (monoisotopic): 378.15, found: 378.17.

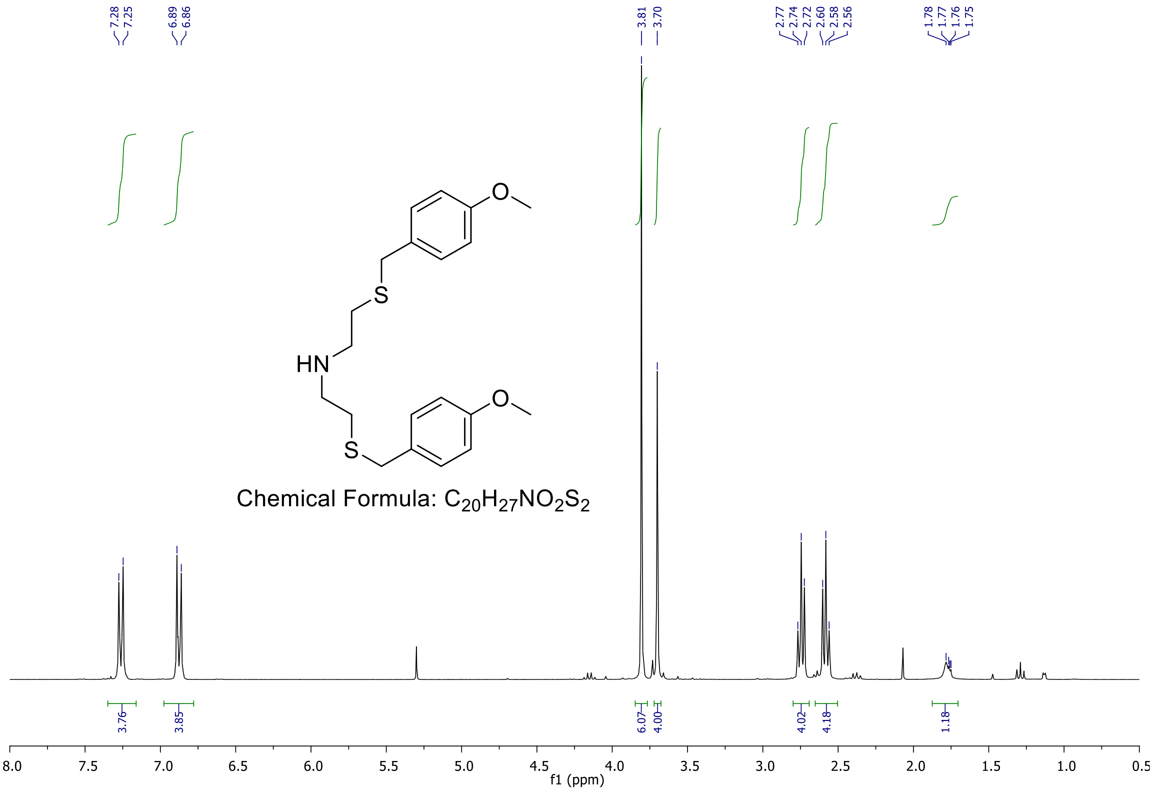

Figure S2. ^{1}H NMR (300 MHz) spectrum of compound **13** ($CDCl_3$, 298K).

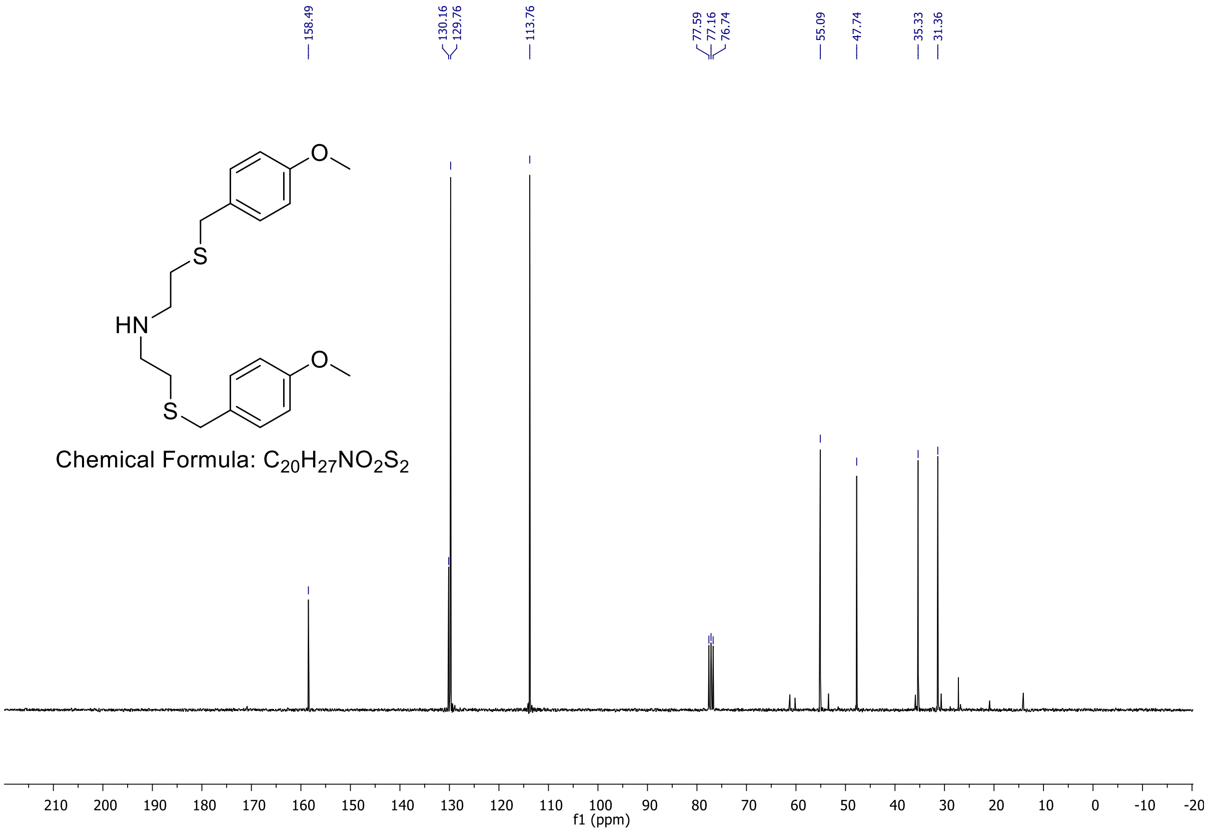

Figure S3. ^{13}C NMR (75 MHz) spectrum of compound **13** ($CDCl_3$, 298K).

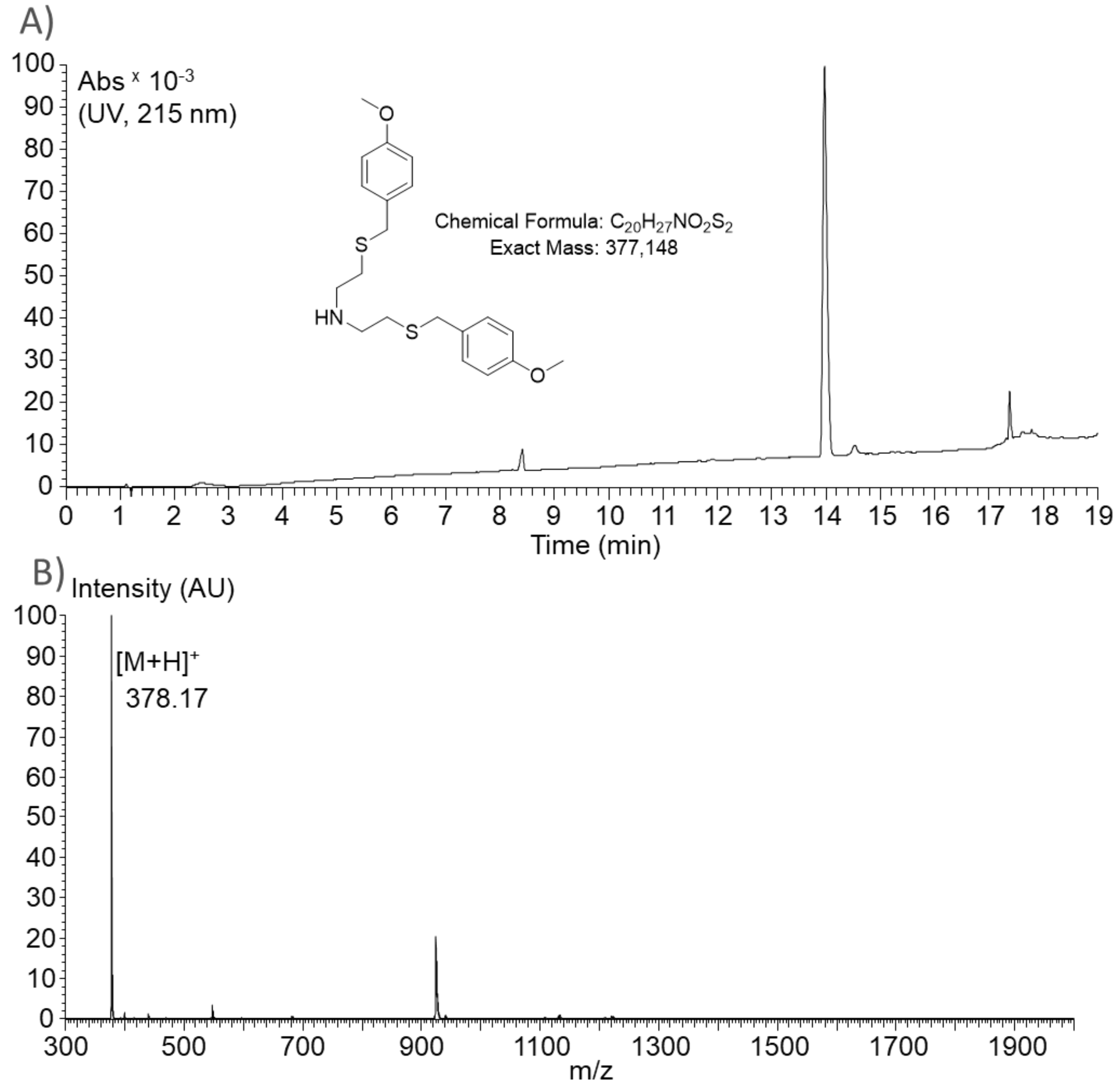

Figure S4. UPLC-MS analysis of compound **13**. A) LC trace. Eluent A 0.1% TFA in water, eluent B 0.1% TFA in CH_3CN. XBridge BEH C18 (3.5 µm, 300 Å, 2.1 × 150 mm), gradient 0-50% B in 15 min (0.4 mL min^{-1}, detection UV 280 nm). B) MS trace: $[M+H]^+$ m/z calcd. (monoisotopic): 378.15, found: 378.17.

Ethyl 2-(*bis*[2-((4-methoxybenzyl)sulfenyl)ethyl]amino)-2-oxoacetate **14**

To a solution of *bis*[2-((4-methoxybenzyl)sulfenyl)ethyl]amine **13** (3.6 g, 9.55 mmol) in DCM (100 mL) is added sodium bicarbonate $NaHCO_3$ (1.6 g, 19.1 mmol). The resulting suspension is cooled to 0 °C under argon and ethyl chlorooxoacetate (2.02 mL, 18.1 mmol) is added

dropwise. The ice bath is removed and the reaction is left to proceed at room temperature during 2 hours. Water (50 mL) is added. The aqueous layer is extracted with DCM (2 × 20 mL). The organic extracts are then combined and washed with water (20 mL), with a saturated bicarbonate aqueous solution (20 mL) and brine (20 mL). The organic layer is dried over $MgSO_4$ and concentrated to dryness under reduced pressure. Product **14** was isolated as a yellow oil (4.4 g, 96%).

R_f 0.32 (Eluent: Cyclohexane/Ethyl acetate 80/20). **^{1}H NMR** (300 MHz, $CDCl_3$, Figure S5) δ 7.24 (d, *J* = 8.3 Hz, 4H), 6.88-6.85 (dd, *J* = 8.6, 1.8 Hz, 4H), 4.31 (q, *J* = 7.1 Hz, 2H), 3.80 (2 s, 6H), 3.70 (s, 2H), 3.66 (s, 2H), 3.41-3.31 (m, 4H), 2.59-2.52 (m, 4H), 1.35 (t, *J* = 7.2 Hz, 3H) ppm. **^{13}C NMR** (75 MHz, $CDCl_3$, Figure S6) δ 162.4, 161.5, 158.9, 158.8, 130.1, 130.0, 129.7, 114.1, 114.0, 62.3, 53.3, 48.6, 45.4, 35.9, 35.7, 29.7, 28.1, 14.0 ppm. **IR** (ATR, cm^{-1}) 2936, 2836, 1732, 1654, 1609, 1510, 1243, 1173, 1155, 1031.

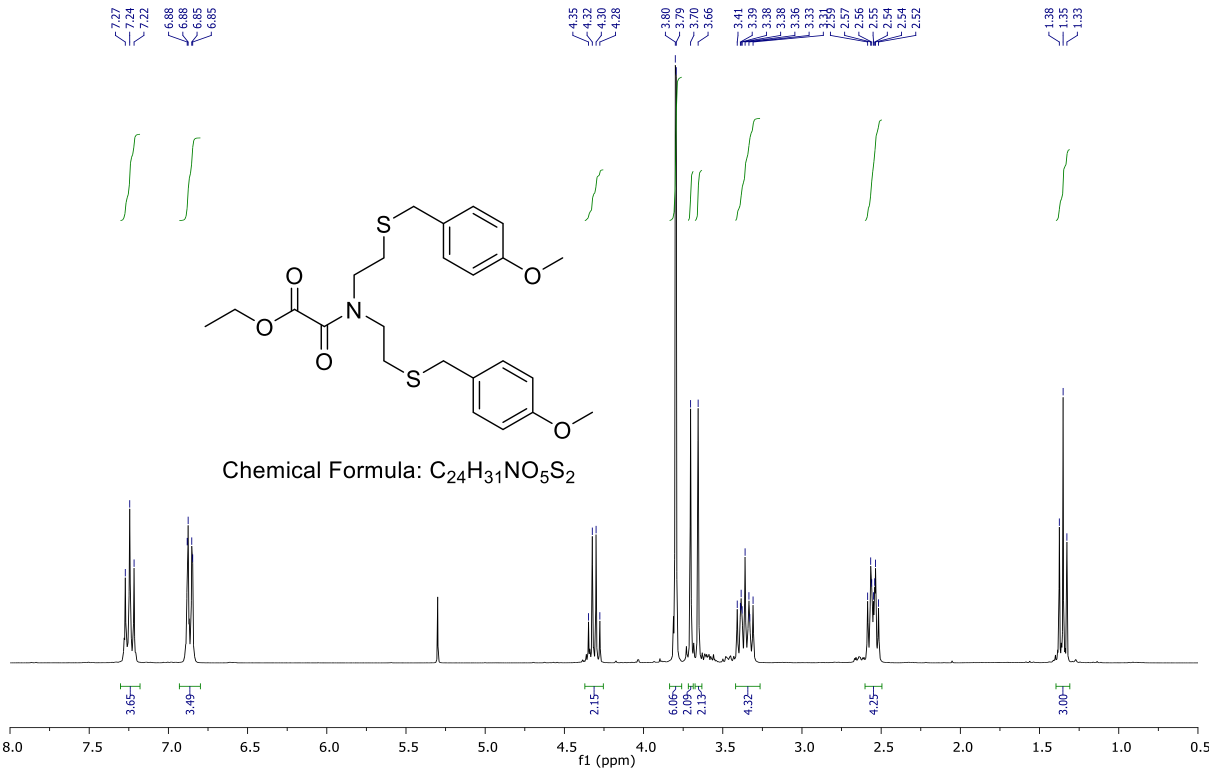

Figure S5. ^{1}H NMR (300 MHz) spectrum of compound **14** ($CDCl_3$, 298K).

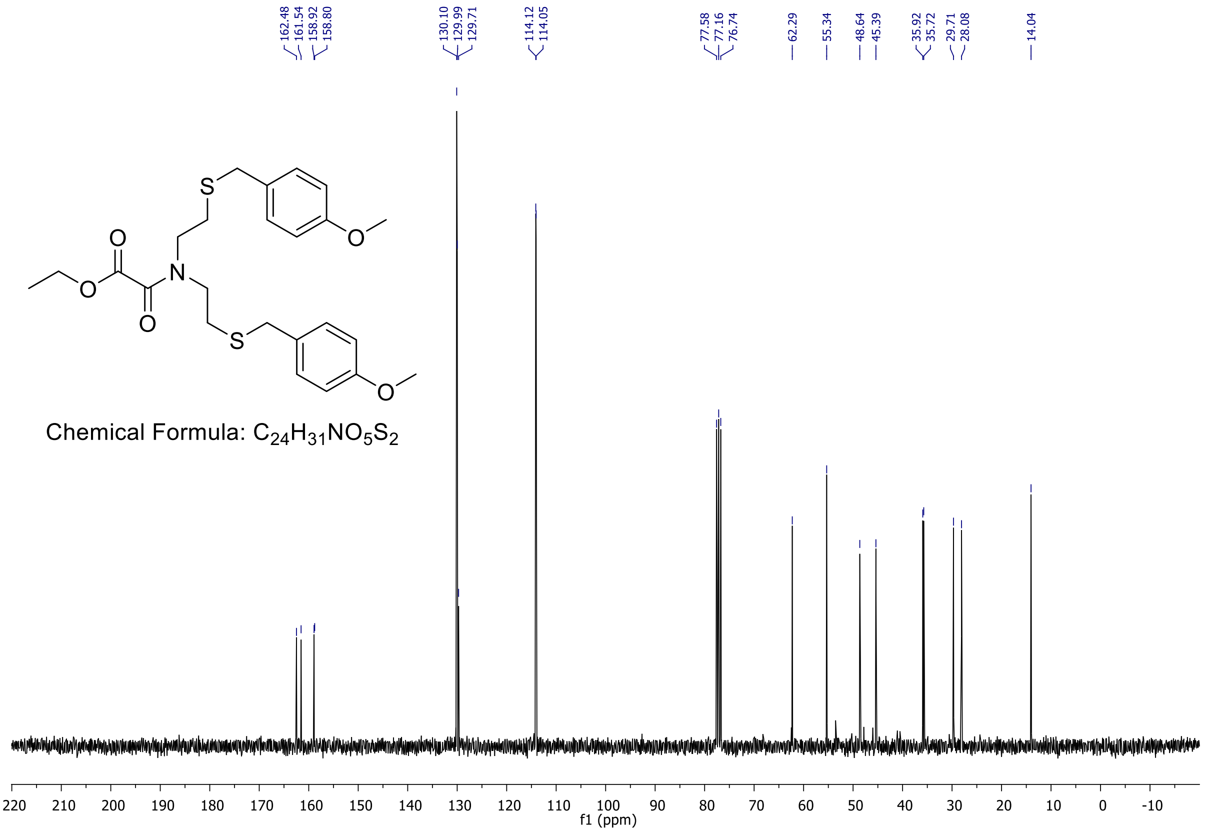

Figure S6. ^{13}C NMR (75 MHz) spectrum of compound **14** ($CDCl_3$, 298K).

<u>2-(*bis*[2-((4-methoxybenzyl)sulfenyl)ethyl]amino)-2-oxoacetic acid **3**</u>

To a solution of intermediate **14** (4.40 g, 9.2 mmol) in THF (120 mL) is added an aqueous solution of sodium hydroxide (2.17 g, 54.3 mmol) in water (120 mL). The resulting solution is stirred at room temperature during 2 hours. The crude mixture is acidified to pH 2 by addition of 1 M HCl. The aqueous layer is extracted with ethyl acetate (3 ×60 mL). Organic extracts are combined, washed with water (3 × 25 mL), brine (25 mL), dried over $MgSO_4$ and concentrated under reduced pressure. Product **3** was isolated as a yellow oil (4.10 g, 99%).

^{1}H NMR (300 MHz, $CDCl_3$, Figure S7) δ 10.51 (s, 1H), 7.26-7.22 (dd, *J* = 8.5, 2.0 Hz, 4H), 6.87 (d, *J* = 8.2 Hz, 4H), 3.79 (s, 6H), 3.67 (m, 6H), 3.40 (m, 2H), 2.60-2.53 (m, 4H) ppm. **^{13}C NMR** (75 MHz, $CDCl_3$, Figure S8) δ 161.7, 161.3, 158.8, 130.1, 129.9, 114.1, 55.3, 49.2, 47.2, 35.7, 35.7, 29.9, 27.9 ppm. **IR** (ATR, cm^{-1}) 2924, 1741, 1596, 1510, 1434, 1254, 1229, 1180, 1032.

Acid **3** decomposes during mass analysis (ESI)

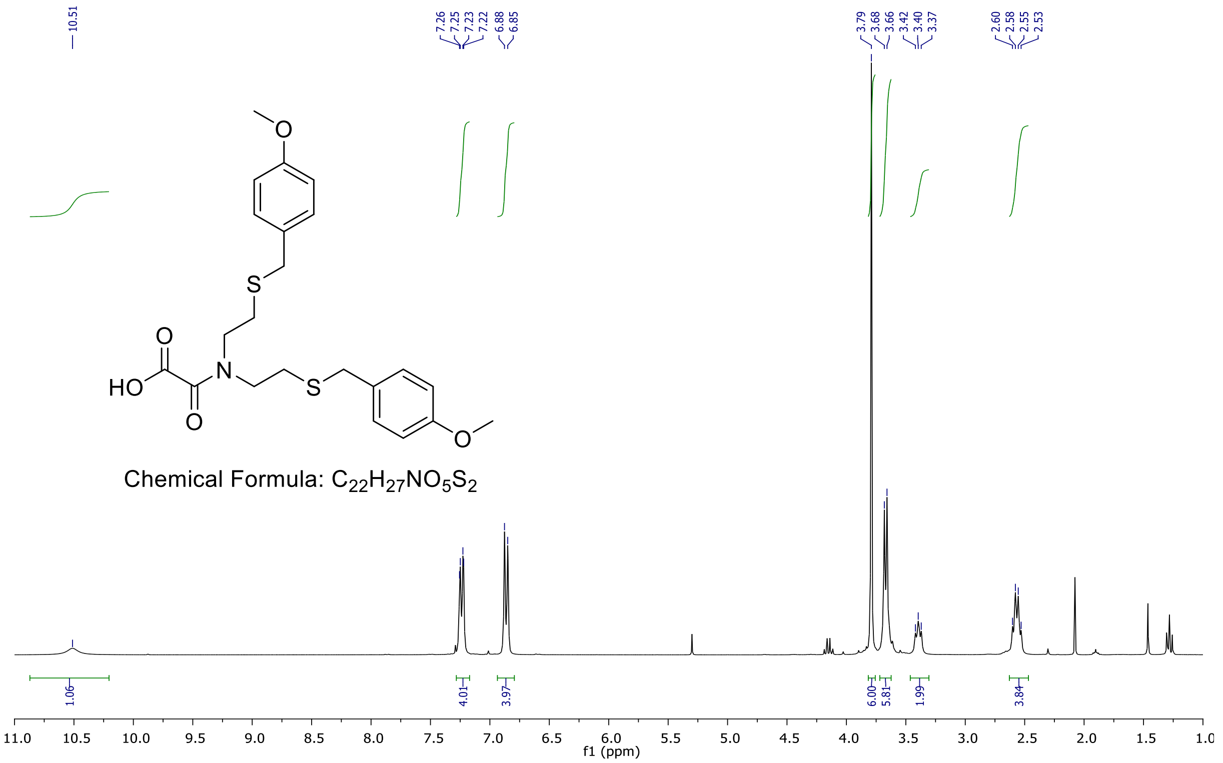

Figure S7. ^{1}H NMR (300 MHz) spectrum of compound **3** ($CDCl_3$, 298K).

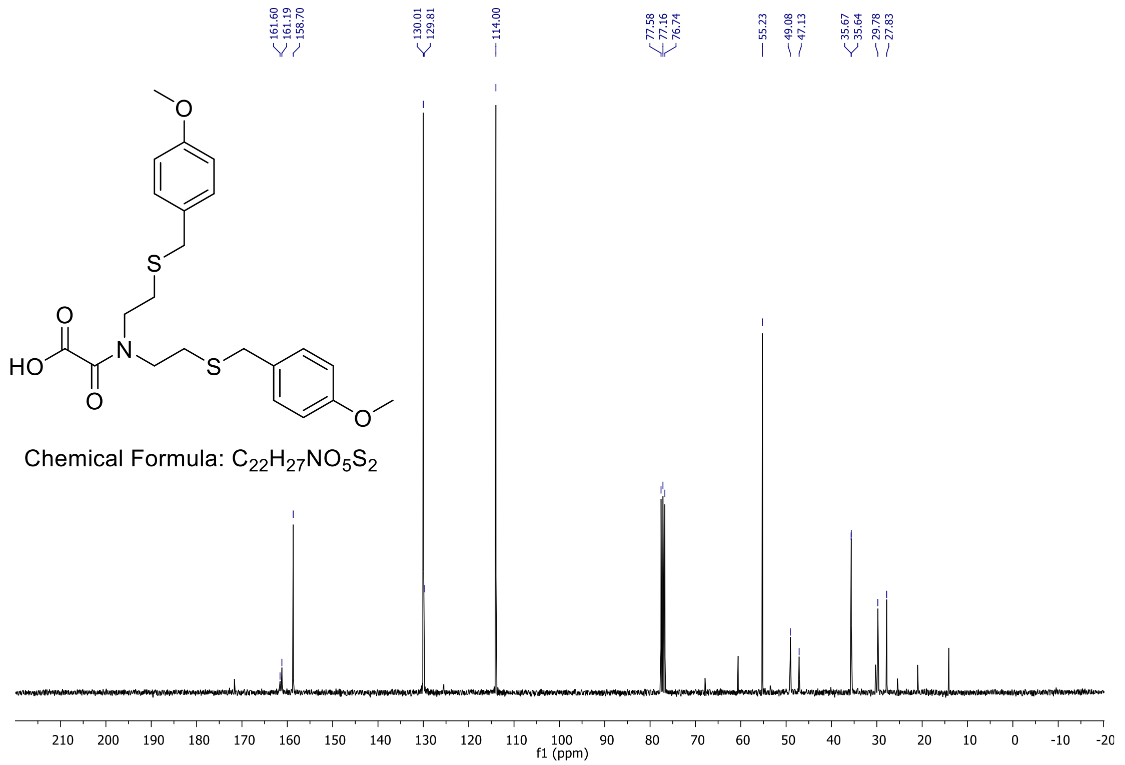

Figure S8. ^{13}C NMR (75 MHz) spectrum of compound **3** ($CDCl_3$, 298K).

Boc-Lys(^{oxo}SEA-Mob)-O^{t}Bu **4**

The coupling reaction of the oxalic acid derivative **3** involves highly reactive species which might decompose upon prolonged activation times. Therefore, all the reagents were predissolved in dry DMF as follows:

- Solution 1: acid **3** (2.34 g, 5.21 mmol) and *N,N*-diisopropylethylamine (2.4 mL, 13.89 mmol) were dissolved in DMF (14 mL).
- Solution 2: HATU (1.89 g, 4.96 mmol) was dissolved in DMF (8 mL)
- Solution 3: Boc-Lys-O^{t}Bu intermediate **15** (1.50 g, 4.96 mmol) was dissolved in DMF (8 mL)

Activation of the acid was performed by adding solution 2 to solution 1. The mixture was stirred 25 sec at room temperature (turns orange) and was then immediately added to solution 3. After 1 h at room temperature, the crude was analyzed by TLC showing complete consumption of the starting materials.

An aqueous saturated solution of ammonium chloride (100 mL) and ethyl acetate (100 mL) were added to the reaction. The two layers were separated. The aqueous layer was extracted with ethyl acetate (2 × 40 mL). The organic extracts were combined, washed with saturated NH_4Cl (6 × 40 mL), dried over $MgSO_4$ and concentrated under reduced pressure. The crude mixture was purified by silica gel column chromatography (Eluent: Cyclohexane/Ethyl acetate 70/30). Product **4** was isolated as a colorless glass (2.91 g, 80%).

R_f 0.29 (Eluent: Cyclohexane/Ethyl acetate 70/30). **^{1}H NMR** (300 MHz, $CDCl_3$, Figure S9) δ 7.51 (m, 1H), 7.26 (dd, *J* = 8.6, 2.6 Hz, 4H), 6.86 (d, *J* = 7.8 Hz, 4H), 5.20 (d, *J* = 8.2 Hz, 1H), 4.18-4.16 (m, 1H), 3.82 (m, 2H), 3.79 (s, 6H), 3.70 (s, 4H), 3.38 (m, 2H), 3.27 (m, 2H), 2.65-2.53 (m, 4H), 1.80-1.57 (m, 5H), 1.46 (2 s, 18H) ppm. **^{13}C NMR** (75 MHz, $CDCl_3$, Figure S10) δ 171.7, 161.9, 160.6, 158.5, 158.5, 155.2, 130.0, 129.9, 129.9, 129.8, 113.8, 113.7, 81.5, 79.3, 55.0, 53.6, 53.4, 48.8, 47.8, 39.0, 35.5, 35.3, 32.3, 30.1, 28.7, 28.2, 27.8, 22.5 ppm. **IR** (ATR, cm^{-1}) 3332, 2971, 2932, 1708, 1632, 1610, 1511, 1446, 1440, 1365, 1244, 1173, 1151, 1032, 832, 734. **MS** (positive detection mode, Figure S11) $[M+H]^+$ calcd. (monoisotopic, w/o Boc[4]):

---

[4] The Boc group is removed during mass analysis.

634.29, found: 634.17. **HRMS** (ES^+) calcd. for $C_{37}H_{56}N_3O_8S_2$: 734.3503, found: 734.3476 (δ_{ppm} -3.73).

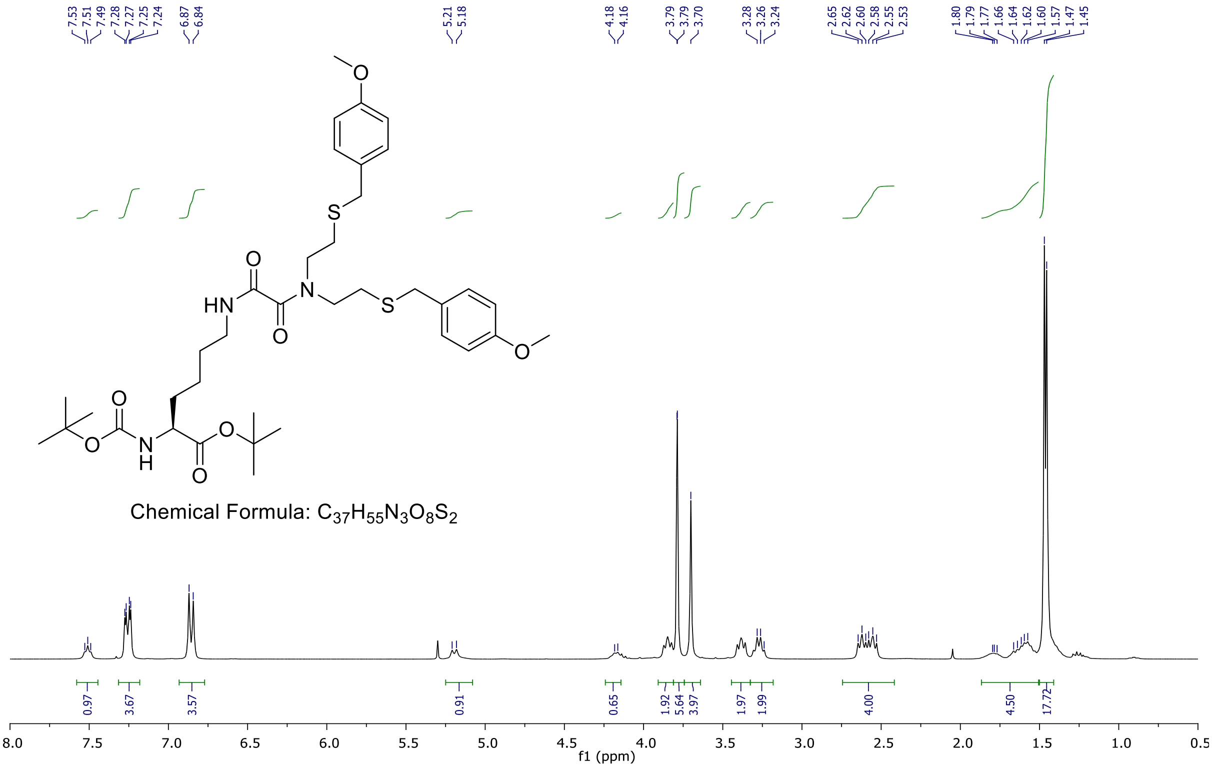

Figure S9. ^{1}H NMR (300 MHz) spectrum of compound **4** ($CDCl_3$, 298K).

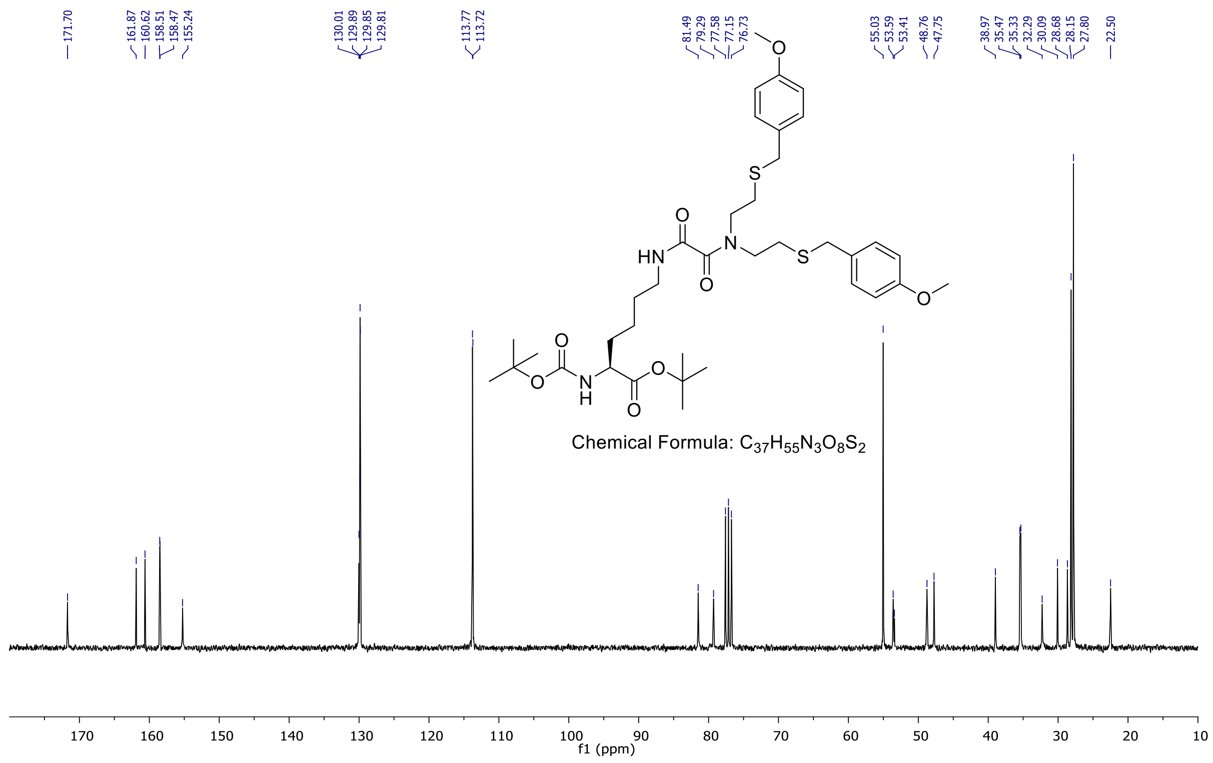

Figure S10. ^{13}C NMR (75 MHz) spectrum of compound **4** ($CDCl_3$, 298K).

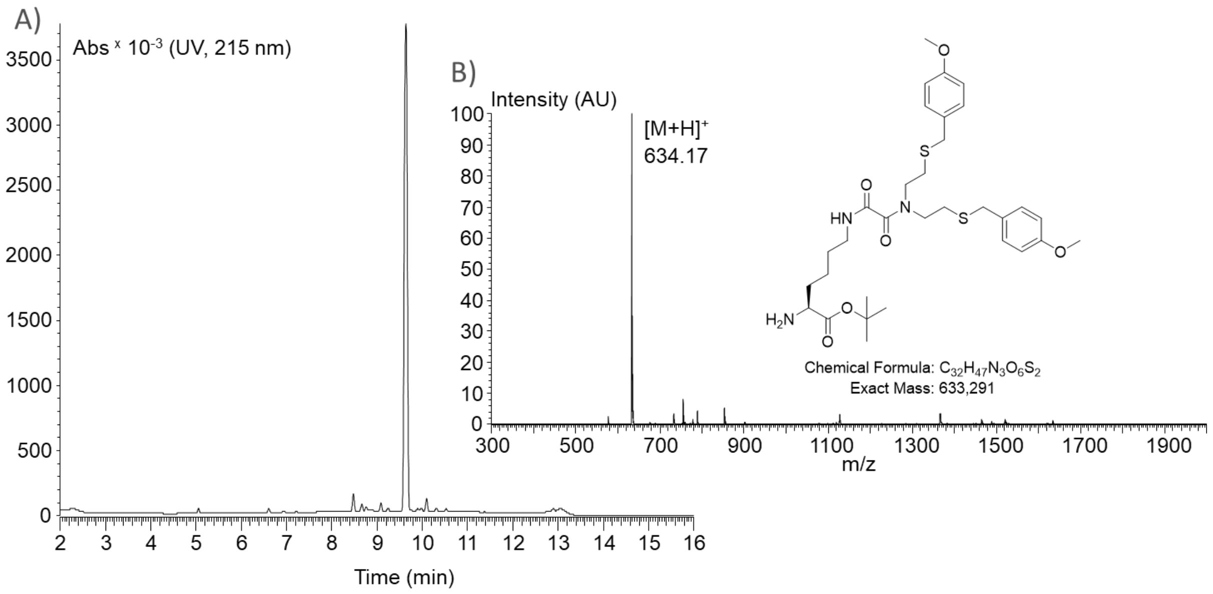

Figure S11. UPLC-MS analysis of intermediate **4**. A) LC trace. Eluent A 0.1% TFA in water, eluent B 0.1% TFA in CH_3CN. XBridge BEH C18 (3.5 µm, 300 Å, 2.1 × 150 mm), gradient 0-50% B in 15 min (0.4 mL min^{-1}, detection UV 215 nm). B) MS trace: $[M+H]^+$ m/z calcd. (monoisotopic w/o Boc[4]): 634.29, found: 634.17.

Fmoc-Lys(^{oxo}SEA-Mob)-OH **1**

The lysine derivative **4** (2.9 g, 4.0 mmol) is dissolved in a solution of HCl 4 M in dioxane (45 mL) at 0 °C. Reaction is stirred overnight at room temperature and under argon atmosphere. Sodium hydrogenocarbonate (1.76 g, 20.9 mmol) is added to the mixture. An aqueous solution of $NaHCO_3$ 10% (10 mL) is added followed by a solution of Fmoc chloroformate (1.09 g, 4.2 mmol) in dioxane (13 mL) at 0 °C. Reaction is stirred at room temperature during 5 hours. Water (40 mL) is added to the mixture and pH is decreased to 1-2 by addition of HCl 1 M. Aqueous layer is extracted with ethyl acetate (3 × 20 mL). Organic layers are washed with brine (20 mL), dried over $MgSO_4$ and concentrated under reduced pressure. The obtained product is purified by column chromatography (Eluent: Cyclohexane/Ethyl acetate 70/30 then pure EtOAc). Product **1** was isolated as a white amorphous solid (1.7 g, 53%).

^{1}H NMR (300 MHz, $CDCl_3$, Figure S12) δ 8.79 (m, 1H, COOH), 7.74 (d, *J* = 7.4 Hz, 2H), 7.62-7.57 (m, 3H), 7.37 (t, *J* = 7.3 Hz, 2H), 7.30-7.19 (m, 6H), 6.83 (m, 4H,), 5.79 (d, *J* = 8.0 Hz, 1H,), 4.45-4.36 (m, 3H,), 4.19 (m, 1H,), 3.81 (m, 2H,), 3.76 (s, 6H,), 3.65 (s, 4H,), 3.36-3.25 (m, 4H,), 2.60-2.49 (m, 4H), 1.90-1.43 (m, 6H,) ppm. **^{13}C NMR** (75 MHz, $CDCl_3$, Figure S13) δ 175.5, 162.4, 160.9, 158.7, 156.3, 143.9, 143.8, 141.3, 130.1, 130.1, 130.0, 127.7, 127.1, 125.2, 120.0, 114.0, 114.0, 67.1, 55.3, 49.0, 48.0, 47.1, 39.1, 35.8, 35.6, 31.8, 30.2, 28.6, 28.0, 22.5 ppm. **IR** (ATR, cm^{-1}) 3391, 3303, 2944, 1691, 1666, 1610, 1534, 1513, 1248, 1174. **MS** (positive detection mode, Figure S15) $[M+H]^+$ calcd. (monoisotopic): 800.30, found: 801.67. **HRMS** (ES^+) calcd. for $C_{43}H_{50}N_3O_8S_2$: 800.3034, found: 800.3029 (δ_{ppm} -0.62).

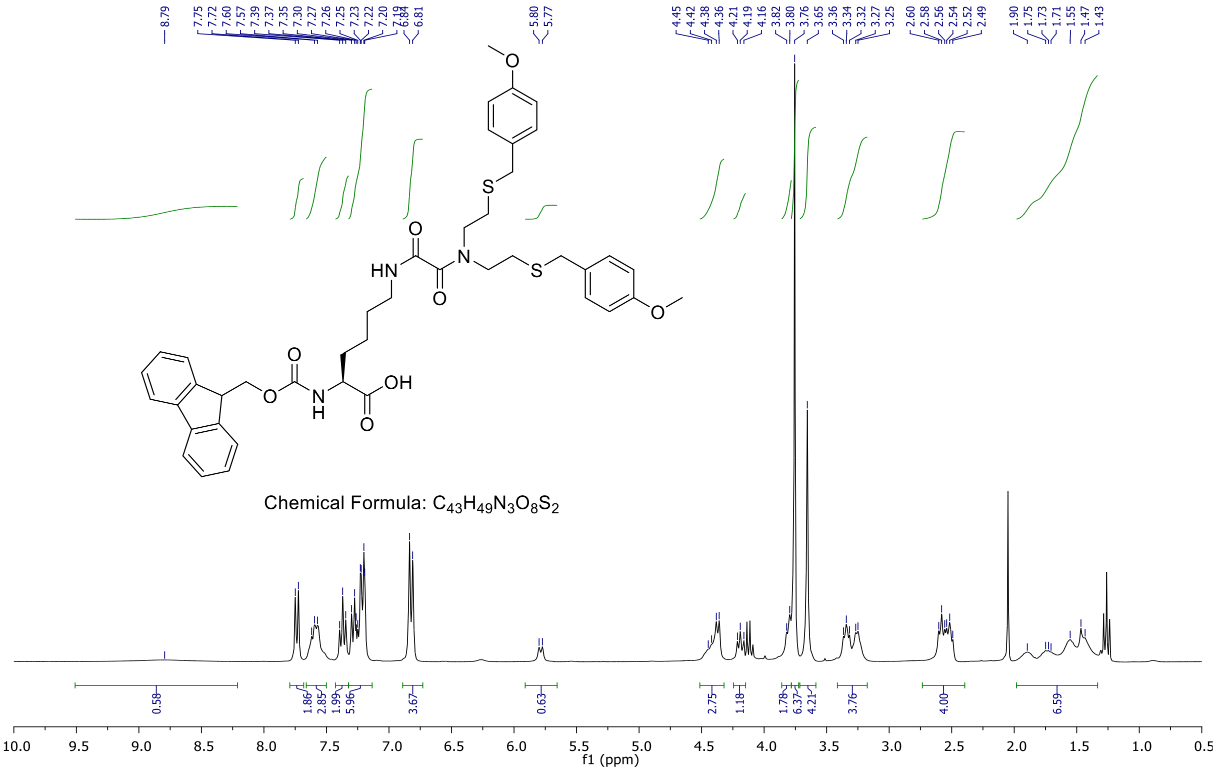

Figure S12. ^{1}H NMR (300 MHz) spectrum of compound **1** ($CDCl_3$, 298K).

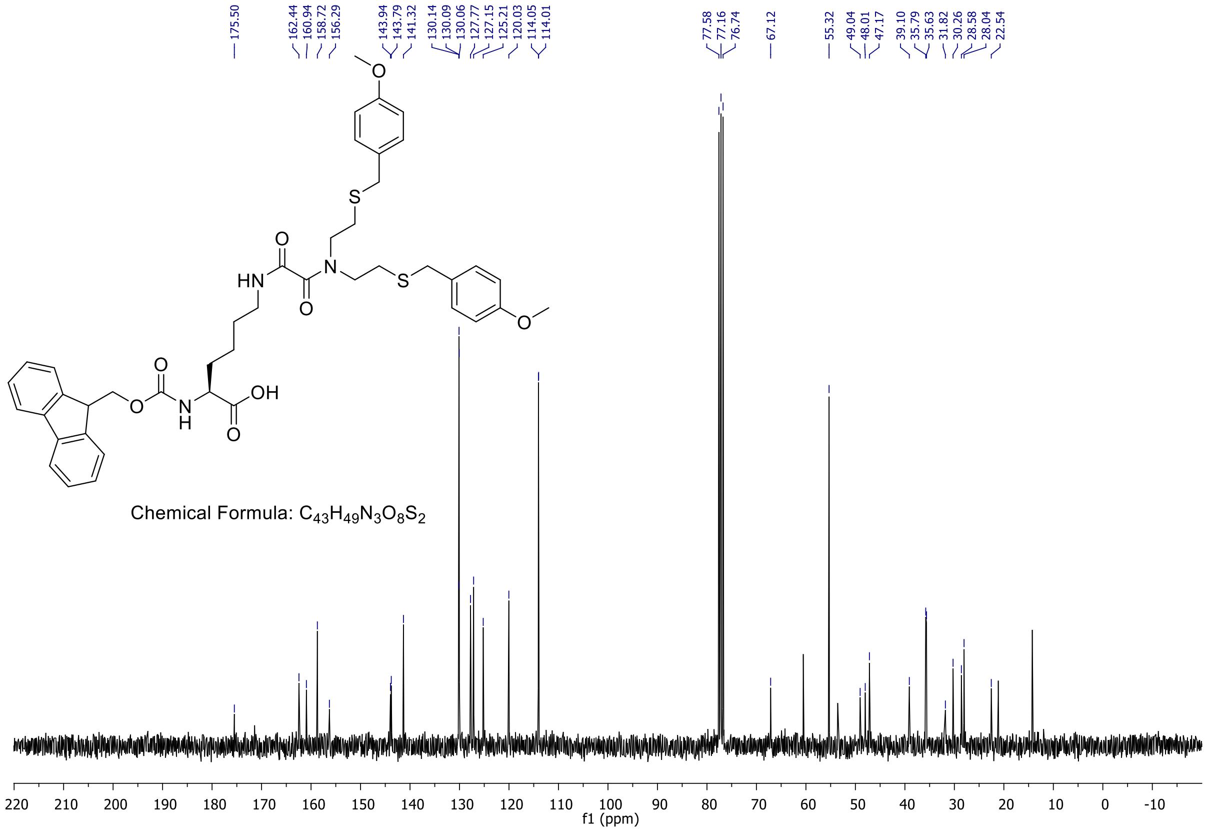

Figure S13. ^{13}C NMR (75 MHz) spectrum of compound **1** ($CDCl_3$, 298K).

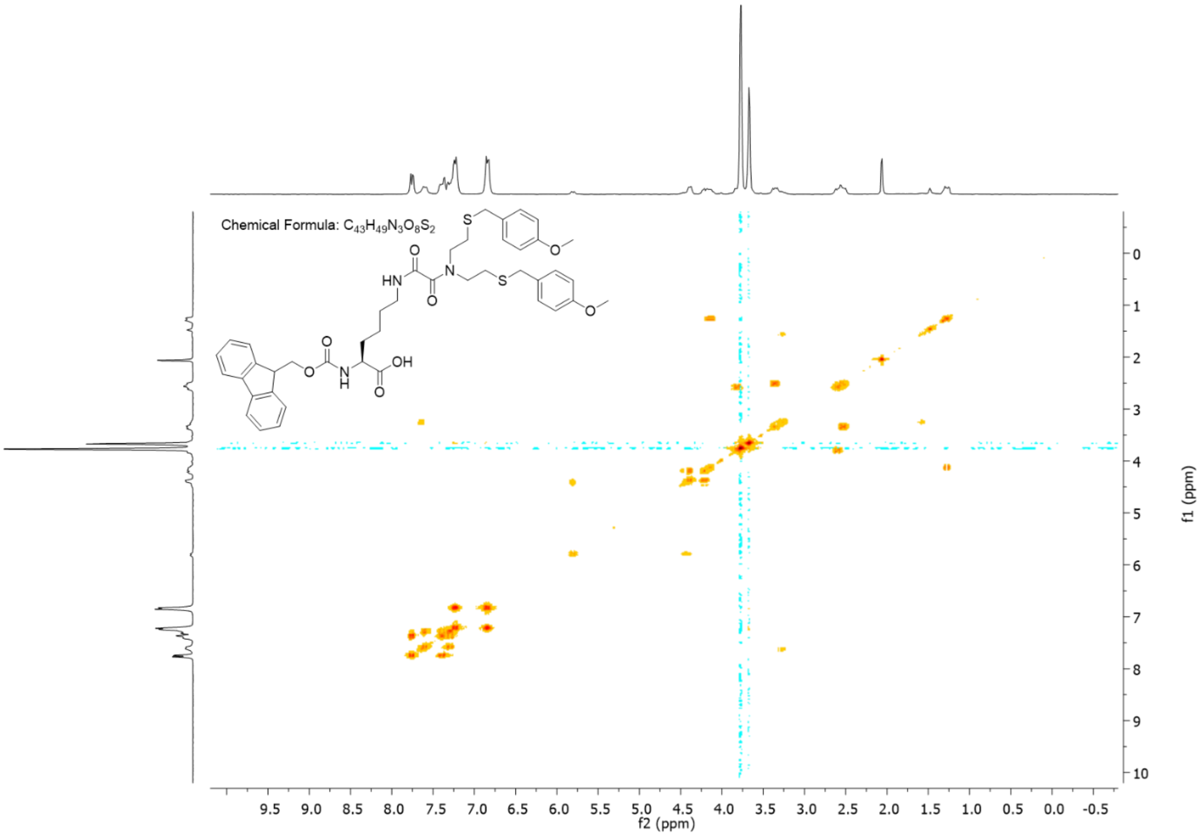

Figure S14. ^1H-^1H (COSY) spectrum of compound **1** ($CDCl_3$, 298K).

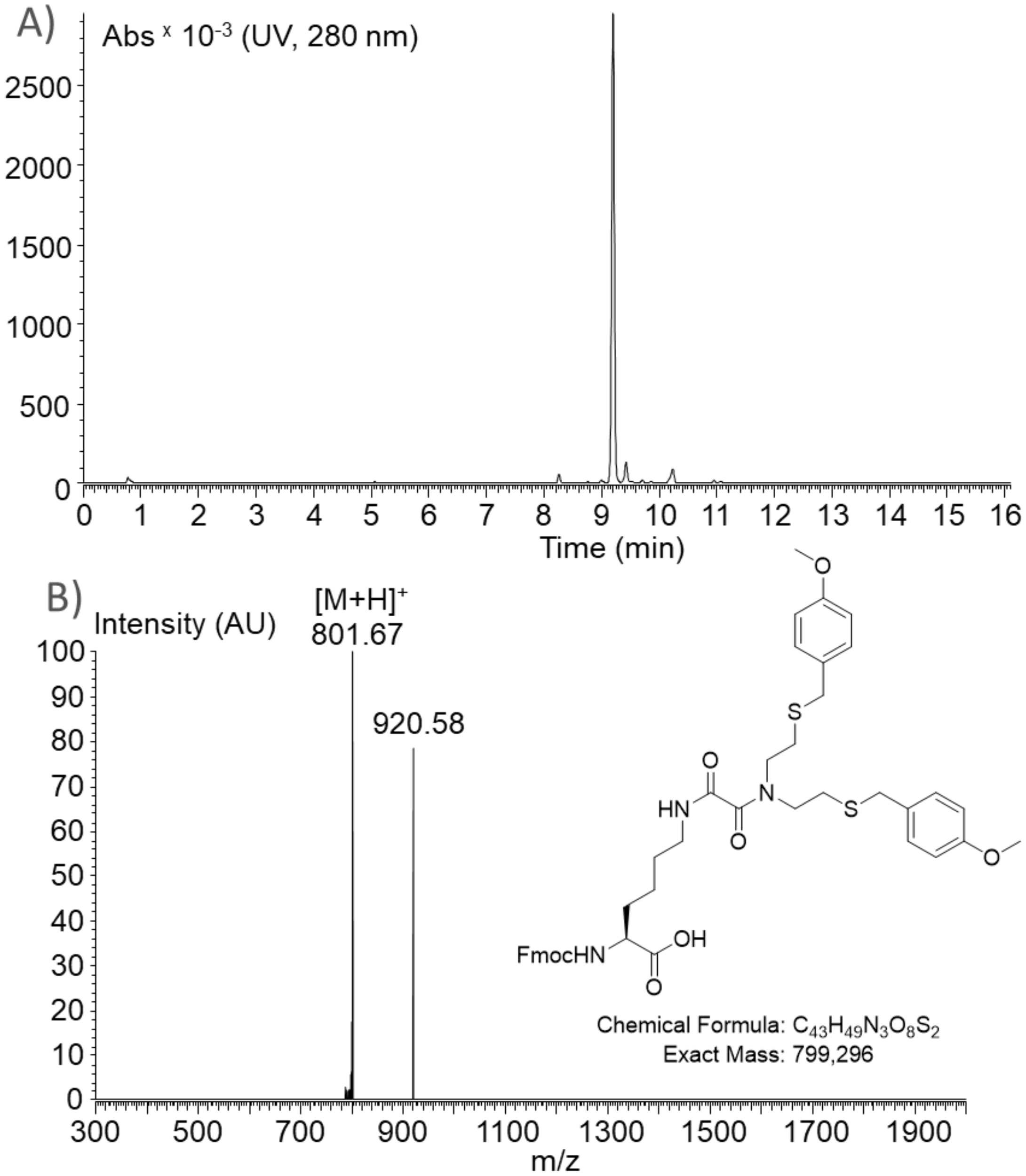

Figure S15. UPLC-MS analysis of compound **1**. A) LC trace. Eluent A 0.1% TFA in water, eluent B 0.1% TFA in CH_3CN. XBridge BEH C18 (3.5 µm, 300 Å, 2.1 × 150 mm), gradient 0-50% B in 15 min (0.4 mL min^{-1}, detection UV 280 nm). B) MS trace: $[M+H]^+$ m/z calcd. (monoisotopic): 800.30, found: 801.67.

3. Synthesis and characterization of model peptides

Amide peptides

Synthesis and characterization of ALREPK(^{oxo}SEA-Mob)HGW-NH_2 peptide **5a**

Peptide **5a** (ALREPK(^{oxo}SEA-Mob)HGW-NH_2) was synthesized on a 0.1 mmol scale as described in the general procedure presented in the Methods section. The peptide was cleaved from the solid support and deprotected using a cocktail TFA/H_2O/EDT/TIS 90/2.5/2.5/5 v/v/v/v (10 mL) during 1h, precipitated in 200 mL of ice-cold Et_2O/heptane 1/1 v/v, solubilized in water and lyophilized. Purification of the crude was performed by preparative RP-HPLC using a preparative C18 XBridge BEH300 column (5 µm, 300 Å, 19 × 150 mm, 50 °C, 215 nm, 20 mL min^{-1}, eluent A: 0.1% by vol. of TFA in water, eluent B: 0.1% by vol. of TFA in acetonitrile, 0-30% B in 10 min, then 30-50% B in 30 min). The purified fractions were combined and lyophilized to give the title peptide as a white solid (11.2 mg, 6%).

MS (ESI, positive detection mode, Figure S16) $[M+H]^+$ m/z calcd. (monoisotopic): 1523.72, found:1524.92.

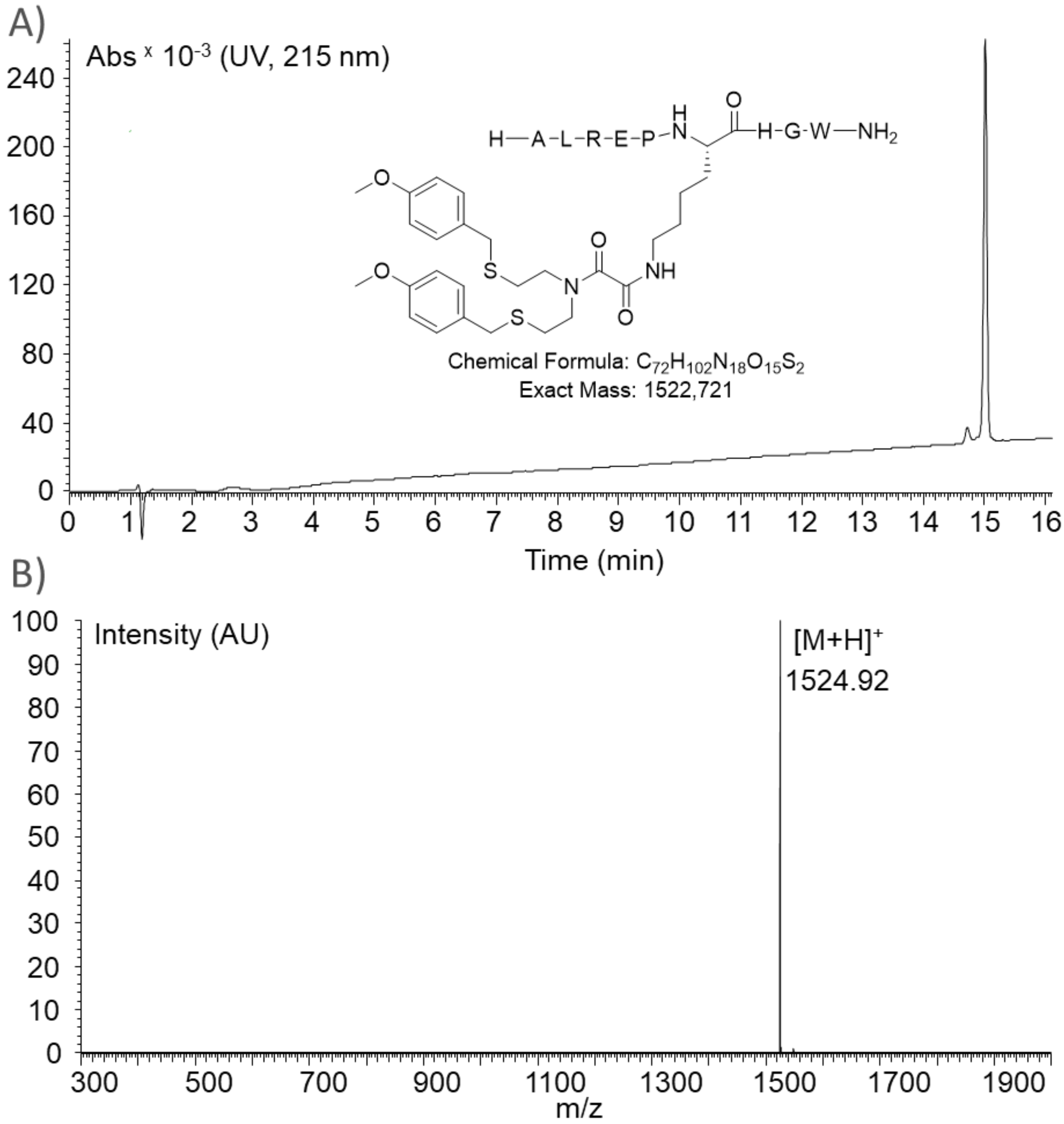

Figure S16. UPLC-MS analysis of ALREPK(^{oxo}SEA-Mob)HGW-NH$_2$ peptide **5a**. A) LC trace. Eluent A 0.1% TFA in water, eluent B 0.1% TFA in CH$_3$CN. XBridge BEH C18 (3.5 µm, 300 Å, 2.1 × 150 mm), gradient 0-50% B in 15 min (0.4 mL min^{-1}, detection UV 215 nm). B) MS trace: $[M+H]^+$ m/z calcd. (monoisotopic): 1523.72, found: 1524.92.

Synthesis and characterization of MALREPK(^{oxo}SEA-Mob)HGW-NH$_2$ peptide **5b**

Peptide **5b** (MALREPK(^{oxo}SEA-Mob)HGW-NH$_2$) was synthesized on a 0.05 mmol scale as described in the general procedure presented in the Methods section. The peptide was cleaved from the solid support and deprotected using a cocktail TFA/H$_2$O/EDT/TIS 90/2.5/2.5/5 v/v/v/v

(5 mL) during 1h, precipitated in 100 mL of ice-cold Et_2O/heptane 1/1 v/v, solubilized in water and lyophilized. Purification of the crude was performed by preparative RP-HPLC using a preparative C18 XBridge BEH300 column (5 µm, 300 Å, 19 × 150 mm, 50 °C, 215 nm, 20 mL min^{-1}, eluent A: 0.1% by vol. of TFA in water, eluent B: 0.1% by vol. of TFA in acetonitrile, 0-30% B in 10 min, then 30-50% B in 30 min). The purified fractions were combined and lyophilized to give the title peptide as a white solid (8.9 mg, 9%).

MS (ESI, positive detection mode, Figure S17) m/z = 1654.67 ($[M+H]^+$), 828.33 ($[M+2H]^{2+}$), 552.58 ($[M+3H]^{3+}$). Calcd. for [M] (average): 1655.03, found: 1654.17.

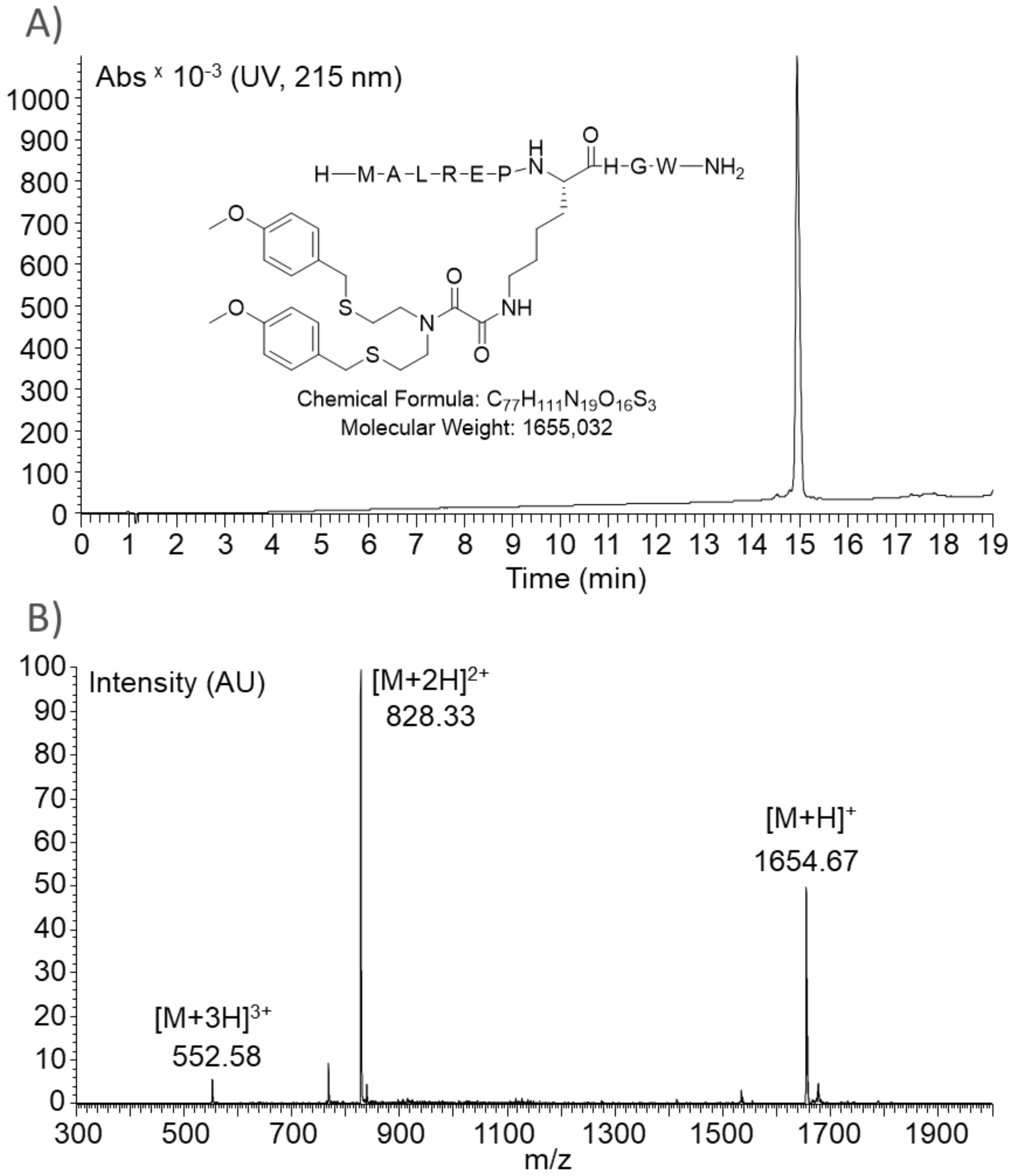

Figure S17. UPLC-MS analysis of MALREPK(oxoSEA-Mob)HGW-NH2 peptide **5b**. A) LC trace. Eluent A 0.1% TFA in water, eluent B 0.1% TFA in CH3CN. XBridge BEH C18 (3.5 µm, 300 Å, 2.1 × 150 mm), gradient 0-50% B in 15 min (0.4 mL min-1, detection UV 215 nm). B) MS trace: m/z = 1654.67 ($[M+H]^{+}$), 828.33 ($[M+2H]^{2+}$), 552.58 ($[M+3H]^{3+}$); Calcd. for [M] (average): 1655.03, found: 1654.17.

Synthesis and characterization of CALREPK(^{oxo}SEA-Mob)HGW-NH_2 peptide **5c**

Peptide **5c** (CALREPK(^{oxo}SEA-Mob)HGW-NH_2) was synthesized on a 0.1 mmol scale as described in the general procedure presented in the Methods section. The peptide was cleaved from the solid support and deprotected using a cocktail TFA/H_2O/EDT/TIS 90/2.5/2.5/5 v/v/v/v (10 mL) during 1h, precipitated in 200 mL of ice-cold Et_2O/heptane 1/1 v/v, solubilized in water and lyophilized. Purification of the crude was performed by preparative RP-HPLC using a preparative C18 XBridge BEH300 column (5 µm, 300 Å, 19 × 150 mm, 50 °C, 215 nm, 20 mL min^{-1}, eluent A: 0.1% by vol. of TFA in water, eluent B: 0.1% by vol. of TFA in acetonitrile, 0-30% B in 10 min, then 30-50% B in 30 min). The purified fractions were combined and lyophilized to give the title peptide as a white solid (15.6 mg,8%).

MS (ESI, positive detection mode, Figure S18) m/z = 1627.92 ([M+H]$^+$), 815.33 ([M+2H]$^{2+}$). Calcd. for [M] (average): 1626.98, found:1627.79.

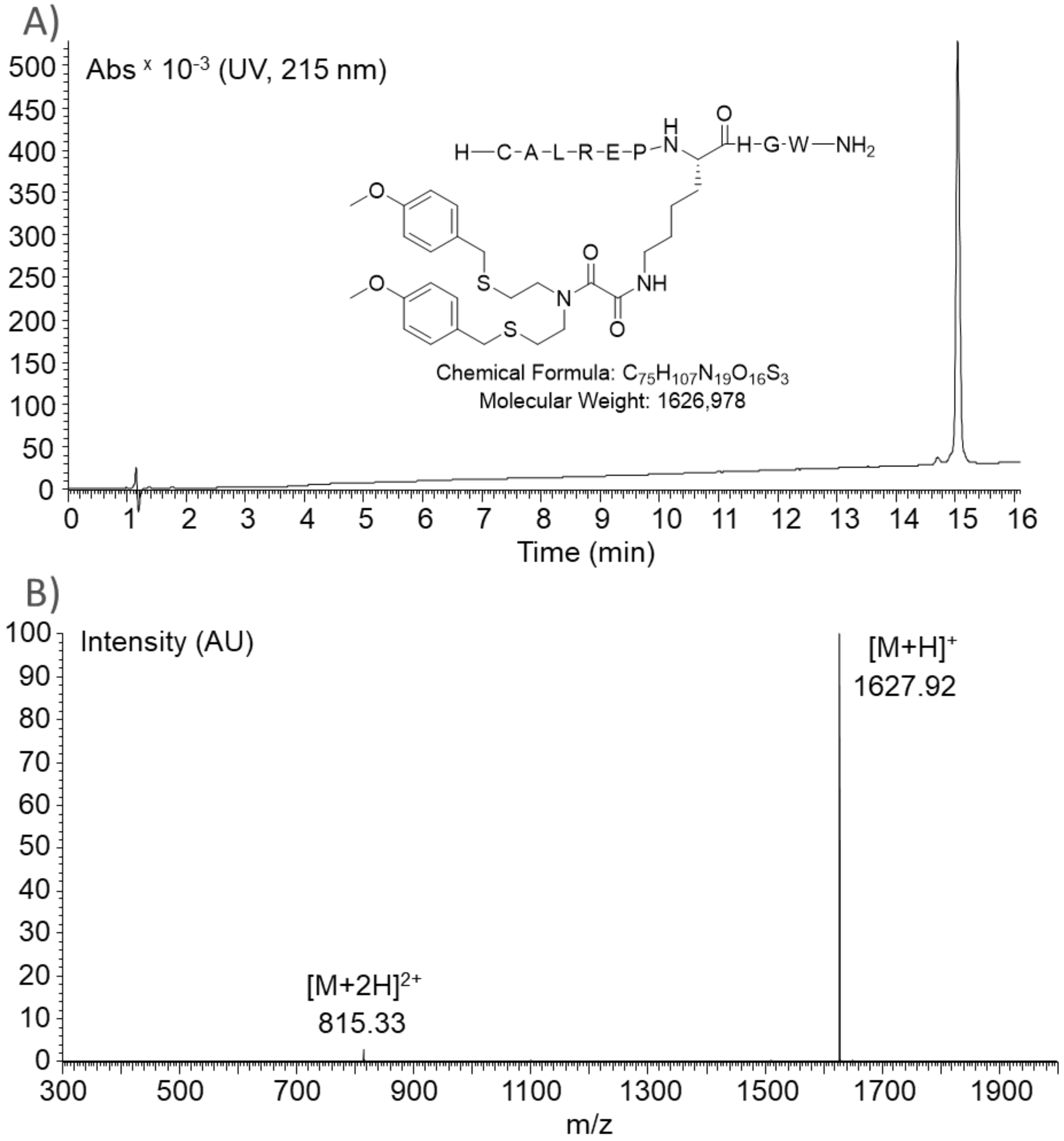

Figure S18. UPLC-MS analysis of CALREPK(^{oxo}SEA-Mob)HGW-NH_2 peptide **5c**. A) LC trace. Eluent A 0.1% TFA in water, eluent B 0.1% TFA in CH_3CN. XBridge BEH C18 (3.5 µm, 300 Å, 2.1 × 150 mm), gradient 0-50% B in 15 min (0.4 mL min^{-1}, detection UV 215 nm). B) MS trace: m/z = 1627.92 ([M+H]$^+$), 815.33 ([M+2H]$^{2+}$); Calcd. for [M] (average): 1626.98, found: 1627.79.

Synthesis and characterization of CILKEPVHGA-NH_2 peptide **12**

Peptide **12** (CILKEPVHGA-NH_2) was synthesized on a 0.1 mmol scale as described in the general procedure presented in the Methods section. The peptide was cleaved from the solid support and deprotected using a cocktail TFA/H_2O/EDT/TIS 90/2.5/2.5/5 v/v/v/v (10 mL) during 2h, precipitated in 200 mL of ice-cold Et_2O/heptane 1/1 v/v, solubilized in water and lyophilized. Purification of the crude was performed by preparative RP-HPLC using a preparative C18 XBridge BEH300 column (5 µm, 300 Å, 19 × 150 mm, 50 °C, 215 nm, 20 mL min^{-1}, eluent A: 0.1% by vol. of TFA in water, eluent B: 0.1% by vol. of TFA in acetonitrile, 0-15% B in 5 min, then 15-22% B in 30 min). The purified fractions were combined and lyophilized to give the title peptide as a white solid (94.3 mg, 67%).

MS (ESI, positive detection mode, Figure S19) m/z = 1066.33 ([M+H]$^+$), 533.58 ([M+2H]$^{2+}$). Calcd. for [M] (average): 1065.30, found:1065.25.

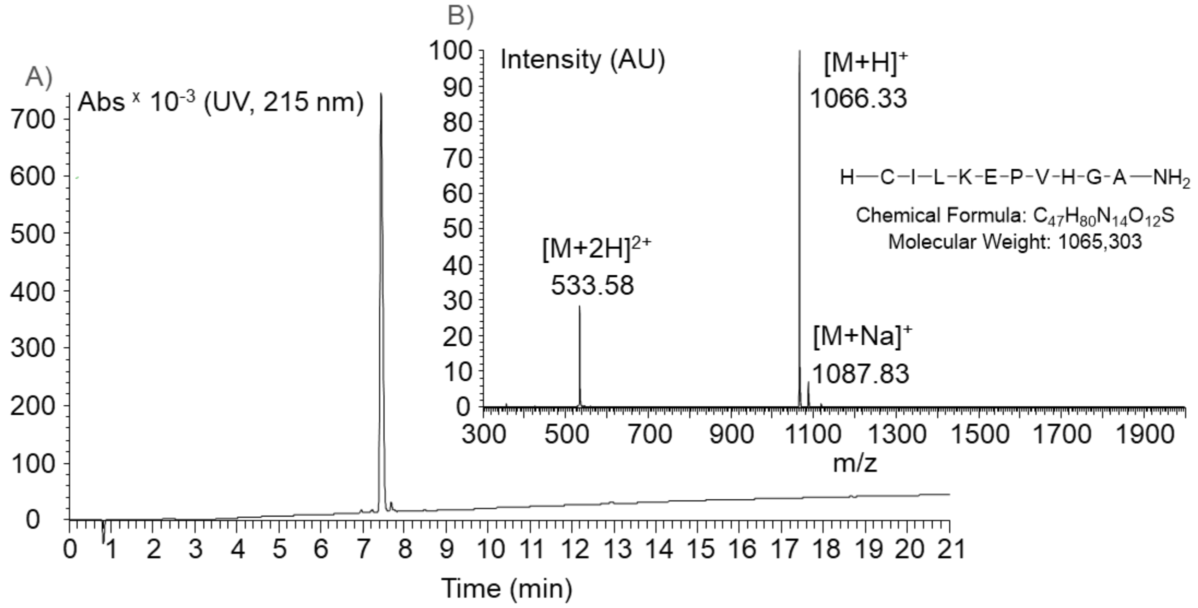

Figure S19. UPLC-MS analysis of CILKEPVHGA-NH_2 peptide **12**. A) LC trace. Eluent A 0.1% TFA in water, eluent B 0.1% TFA in CH_3CN. XBridge BEH C18 (3.5 µm, 300 Å, 2.1 × 150 mm), gradient 0-50% B in 15 min (0.4 mL min^{-1}, detection UV 215 nm). B) MS trace: m/z = 1066.33 ([M+H]$^+$), 533.58 ([M+2H]$^{2+}$); Calcd. for [M] (average): 1065.30, found: 1065.25.

Thioester peptides

<u>Synthesis and characterization of ILKEPVHGA-SEA peptide **7a**</u>

Peptide **7a** (ILKEPVHGA-SEA) was synthesized on a 0.5 mmol scale as described in the general procedure presented in the Methods section. The peptide was cleaved from the solid support and deprotected using a cocktail TFA/H_2O/EDT/TIS 90/2.5/2.5/5 v/v/v/v (20 mL) during 1.5h, precipitated in 400 mL of ice-cold Et_2O/heptane 1/1 v/v, solubilized in water and lyophilized. Crude was dissolved in AcOH/H_2O 1/4 v/v (final peptide concentration 0.5 mM). Iodine solution (200 mM in DMSO; 1.0 mmol; 5.0 mL) was added. After 30 s, DTT (65 mM in water; 1.0 mmol; 15.5 mL) was added to quench the excess of iodine. The mixture was immediately purified by preparative RP-HPLC using a preparative C18 XBridge BEH300 column (5 µm, 300 Å, 19 × 150 mm, 50 °C, 215 nm, 20 mL min^{-1}, eluent A: 0.1% by vol. of TFA in water, eluent B: 0.1% by vol. of TFA in acetonitrile, 0-22% B in 5 min, then 22-35% B in 50 min). The purified fractions were combined and lyophilized to give the title peptide as a white solid (366.0 mg, 51%).

MS (ESI, positive detection mode, Figure S20) m/z = 1080.58 ($[M+H]^+$), 540.92 ($[M+2H]^{2+}$). Calcd. for [M] (average): 1080.38, found: 1079.71.

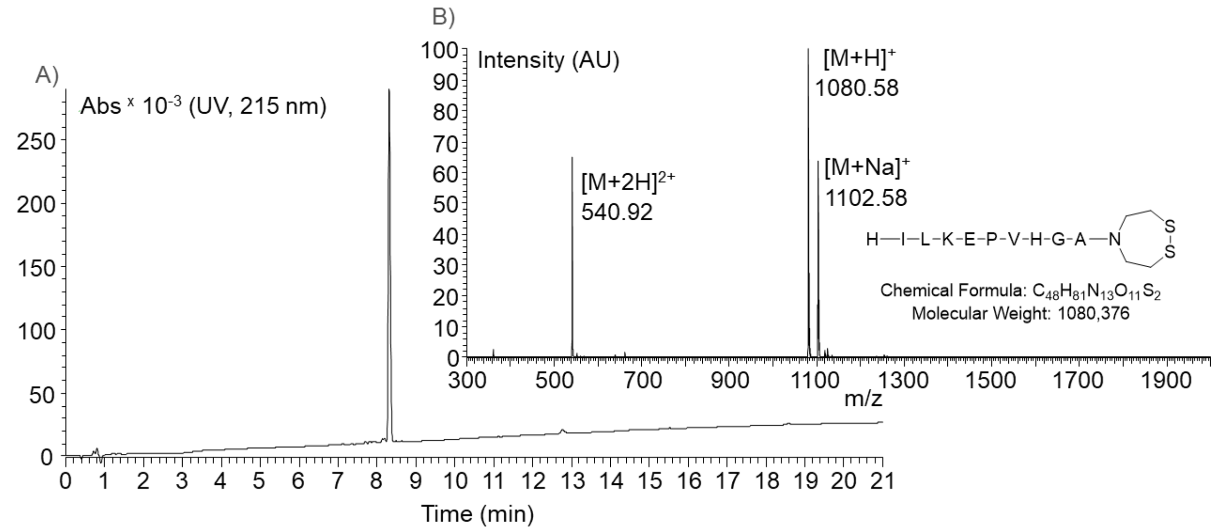

Figure S20. UPLC-MS analysis of ILKEPVHGA-SEA peptide **7a**. A) LC trace. Eluent A 0.1% TFA in water, eluent B 0.1% TFA in CH_3CN. XBridge BEH C18 (3.5 µm, 300 Å, 2.1 × 150 mm), gradient 0-50% B in 15 min (0.4 mL min^{-1}, detection UV 215 nm). B) MS trace: m/z = 1080.58 ($[M+H]^+$), 540.92 ($[M+2H]^{2+}$); Calcd. for [M] (average): 1080.38, found: 1079.71.

Synthesis and characterization of ILKEPVHGA-MPA peptide **16**

The above SEA peptide **7a** (99.65 mg) was dissolved in a 0.1 M TCEP aqueous solution containing 5% v/v 3-mercaptopropionic acid (MPA) of pH 4 (35 mL, 2 mM). The reaction was left to proceed under nitrogen atmosphere at 37 °C overnight (12-15 h). The mixture was quenched by addition of glacial acetic acid (4 mL) and extracted with diethyl ether (4 × 40 mL). Purification of the crude was performed by preparative RP-HPLC using a preparative C18 XBridge BEH300 column (5 µm, 300 Å, 19 × 150 mm, 50 °C, 215 nm, 20 mL min^{-1}, eluent A: 0.1% by vol. of TFA in water, eluent B: 0.1% by vol. of TFA in acetonitrile, 0-10% B in 5 min, then 10-25% B in 45 min). The purified fractions were combined and lyophilized to give the title peptide as a white solid (38.5 mg, 39%).

MS (ESI, positive detection mode, Figure S21) m/z = 1051.67 ($[M+H]^+$), 526.42 ($[M+2H]^{2+}$). Calcd. for [M] (average): 1051.27, found: 1050.76.

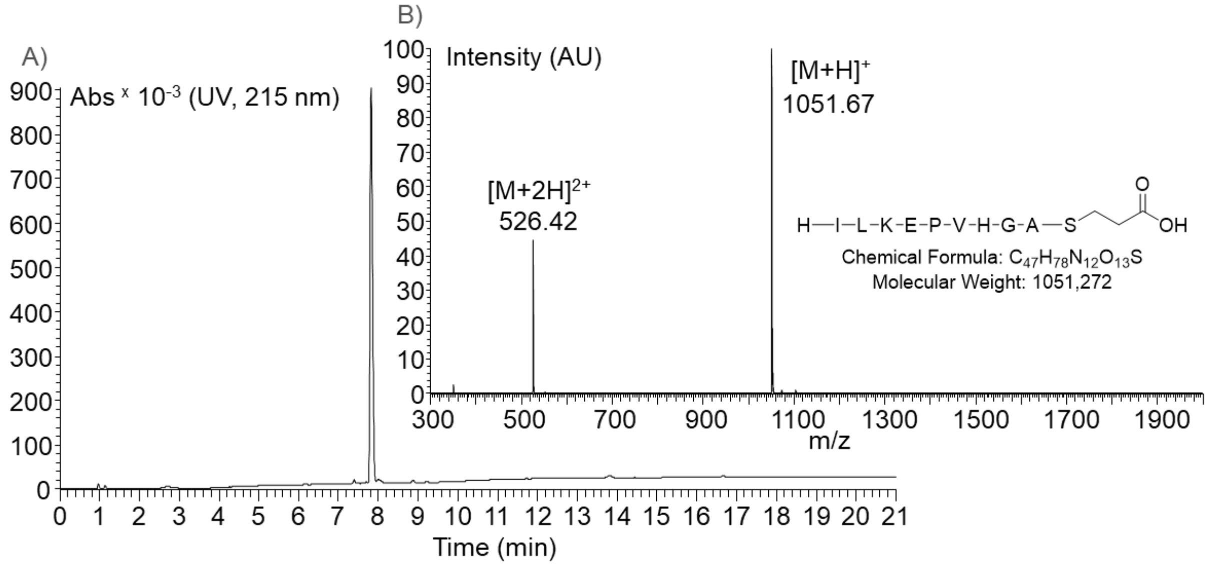

Figure S21. UPLC-MS analysis of ILKEPVHGA-MPA peptide **16**. A) LC trace. Eluent A 0.1% TFA in water, eluent B 0.1% TFA in CH_3CN. XBridge BEH C18 (3.5 µm, 300 Å, 2.1 × 150 mm), gradient 0-50% B in 15 min (0.4 mL min^{-1}, detection UV 215 nm). B) MS trace: m/z = 1051.67 ($[M+H]^+$), 526.42 ($[M+2H]^{2+}$); Calcd. for [M] (average): 1051.27, found: 1050.76.

4. Resistance of Mob groups during peptide cleavage from solid support after SPPS

Cleavage of ALREPK(^{oxo}SEA-Mob)HGW-NH_2 peptide **5a**

Long exposure time to the cleavage cocktail, showed partial to full deprotection of Mob groups. Limiting cleavage to 1 hour allowed to restrain side reactions to basal levels (Figure S22).

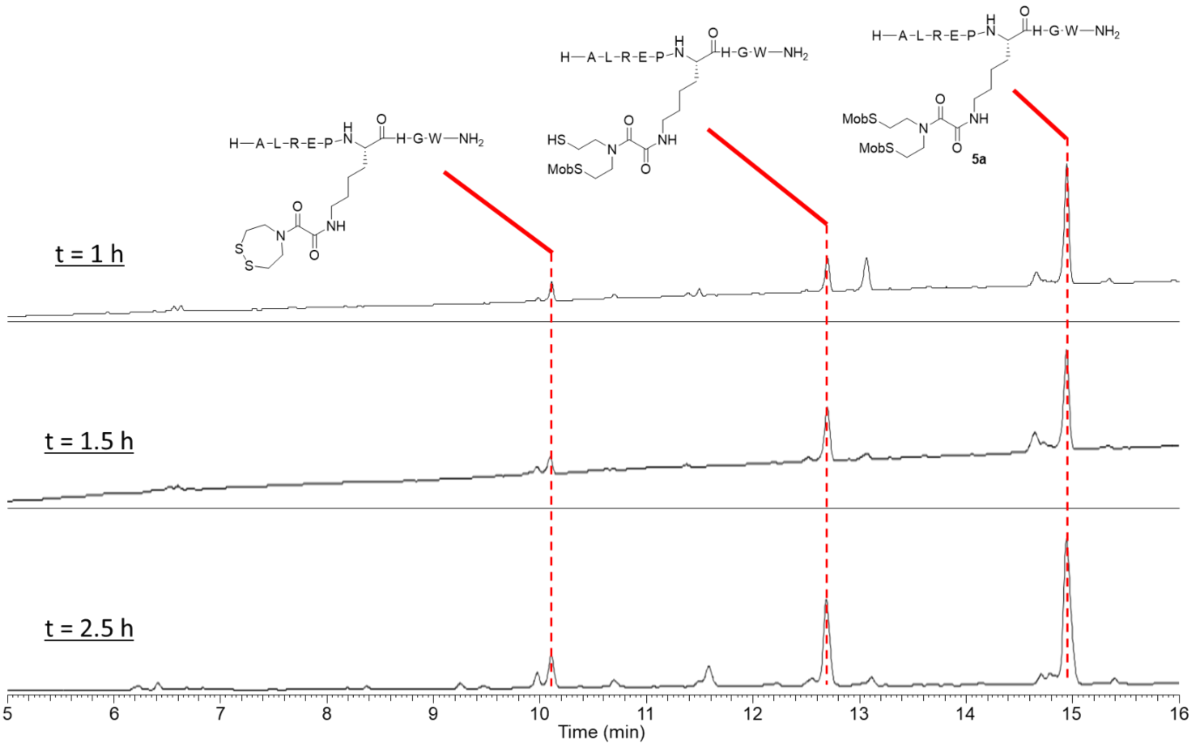

Figure S22. UPLC chromatograms of the monitoring at 215 nm of the cleavage from solid support of peptide **5a** (ALREPK(^{oxo}SEA-Mob)HGW-NH_2) (Cleavage cocktail: TFA/H_2O/EDT/TIS 90/2.5/2.5/5 v/v/v/v) (LC trace. Eluent A 0.1% TFA in water, eluent B 0.1% TFA in CH_3CN. XBridge BEH C18 (3.5 µm, 300 Å, 2.1 × 150 mm), gradient 0-50% B in 15 min (0.4 mL min^{-1}, detection UV 215 nm)).

Cleavage of CALREPK(^{oxo}SEA-Mob)HGW-NH_2 peptide **5c**

Long exposure time to the cleavage cocktail, showed the partial deprotection of Mob group and the formation of a byproduct stemming from the intramolecular Fast-SEA ligation reaction between the ^{oxo}SEA group and the *N*-terminal cysteine residue. Limiting cleavage to 1 hour allowed to restrain side reactions to low levels (Figure S23).

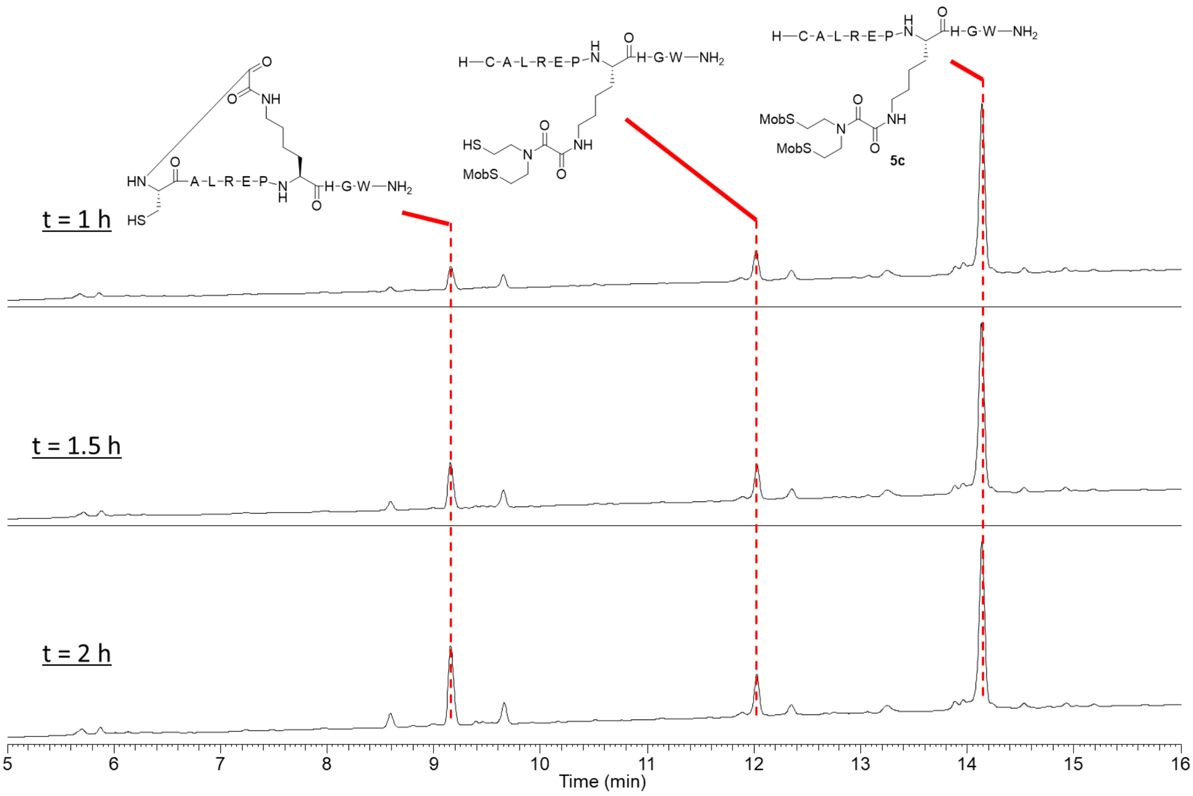

Figure S23. UPLC chromatograms of the monitoring at 215 nm of the cleavage from solid support of peptide **5c** (CALREPK(oxoSEA-Mob)HGW-NH_2) (Cleavage cocktail: TFA/H_2O/EDT/TIS 90/2.5/2.5/5 v/v/v/v) (LC trace. Eluent A 0.1% TFA in water, eluent B 0.1% TFA in CH_3CN. XBridge BEH C18 (3.5 µm, 300 Å, 2.1 × 150 mm), gradient 0-50% B in 15 min (0.4 mL min^{-1}, detection UV 215 nm)).

5. Optimization of Mob cleavage conditions

The Mob cleavage experimental conditions were optimized on peptide **5a** (ALREPK(^{oxo}SEA-Mob)HGW-NH_2) in order to form peptide **6a** (ALREPK(^{oxo}SEA)HGW-NH_2) (Figure S24). Reactions were monitored by analytical UPLC-MS.

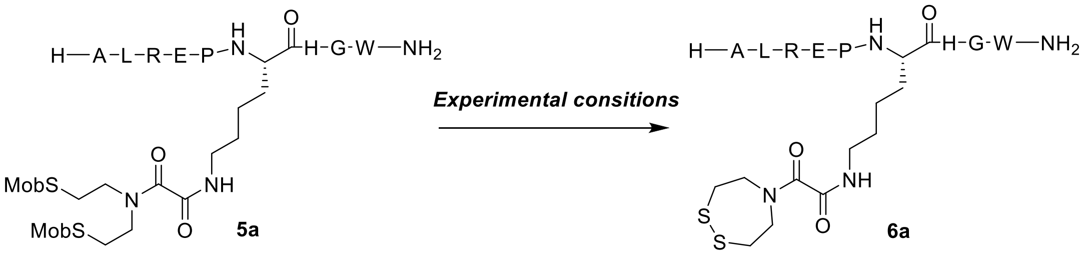

Figure S24. Synthetic scheme for the Mob cleavage and disulfide bond formation from peptide **5a** (ALREPK(^{oxo}SEA-Mob)HGW-NH_2).

<u>Mob cleavage using a TFA/TIS mixture</u>

Peptide **5a** (0.330 mg; 0.18 µmol; 1 mM) was dissolved in 180 µL of a TFA/TIS mixture (95/5 v/v) and the reaction was left to proceed at room temperature. The reaction was monitored by UPLC-MS through sampling of 5 µL aliquots which were quenched by addition of 0.2 M, pH 7.2 phosphate buffer (50 µL) and then extracted with diethyl ether (3 × 100 µL) before injection.

Formation of the target product **6a** was slow and accompanied by the formation of several intermediates and byproducts (Figure S25).[5]

---

[5] Oxidized byproducts ([M+16] and [M+32]) were also reported in previous work: Ste.Marie, E. J.; Hondal, R. J. Reduction of cysteine *S*-protecting groups by triisopropylsilane. *J. Pept. Sci*. **2018**, *24*, e3130.

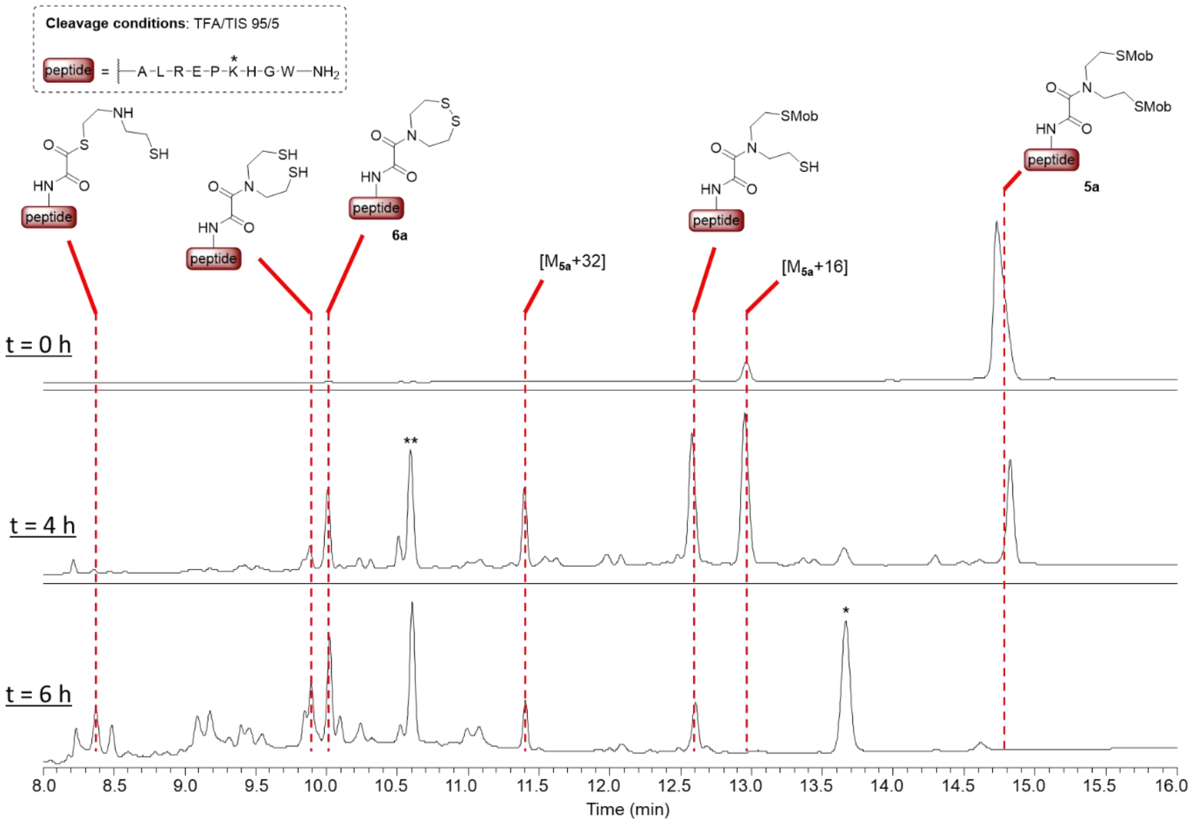

Figure S25. UPLC chromatograms of the monitoring at 215 nm of the Mob cleavage of peptide **5a** (ALREPK(^{oxo}SEA-Mob)HGW-NH$_2$) (Cleavage conditions: TFA/TIS 95/5 v/v) (LC trace. Eluent A 0.1% TFA in water, eluent B 0.1% TFA in CH_3CN. XBridge BEH C18 (3.5 µm, 300 Å, 2.1 × 150 mm), gradient 0-50% B in 15 min (0.4 mL min^{-1}, detection UV 215 nm)). *Non-peptidic impurity. **Mass corresponds to oxidized monodeprotected ^{oxo}SEA group [M_{5a}-M_{Mob}+16].

Mob cleavage using a TFA/thioanisole mixture

Peptide **5a** (0.358 mg; 0.19 µmol; 1 mM) was dissolved in 190 µL of a TFA/thioanisole mixture (95/5 v/v) and the reaction was left to proceed at room temperature. The reaction was monitored by UPLC-MS through sampling of 5 µL aliquots which were quenched by addition of 0.2 M, pH 7.2 phosphate buffer (50 µL) and then extracted with diethyl ether (3 × 100 µL) before injection.

Formation of the target product **6a** was efficient and presented no byproducts but was slow (Figure S26).

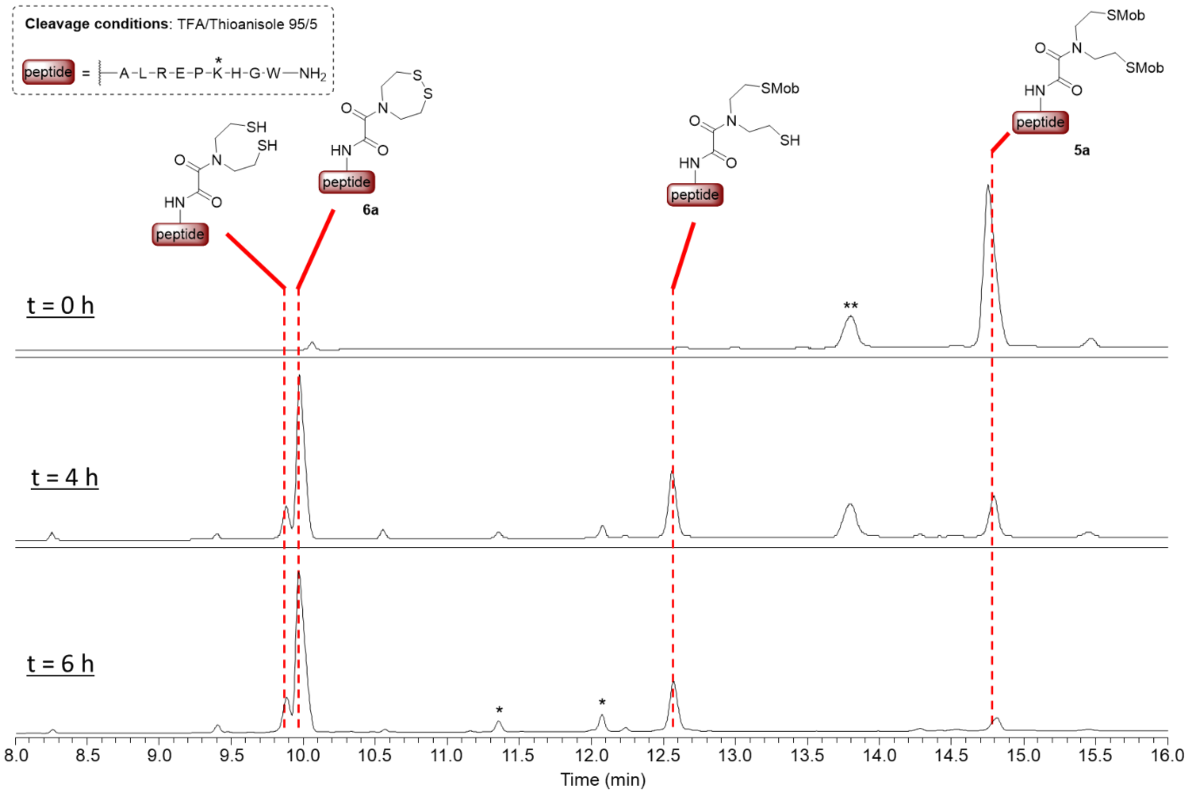

Figure S26. UPLC chromatograms of the monitoring at 215 nm of the Mob cleavage of peptide **5a** (ALREPK(^{oxo}SEA-Mob)HGW-NH$_2$) (Cleavage conditions: TFA/thioanisole 95/5 v/v) (LC trace. Eluent A 0.1% TFA in water, eluent B 0.1% TFA in CH_3CN. XBridge BEH C18 (3.5 µm, 300 Å, 2.1 × 150 mm), gradient 0-50% B in 15 min (0.4 mL min^{-1}, detection UV 215 nm)). *Non-peptidic impurity. **Thioanisole.

Mob cleavage using the diphenylsulfoxide trichloromethylsilane system in TFA

Trichloromethylsilane (0.71 µL; 6 µmol; 100 mM) and peptide **5a** (0.109 mg; 0.06 µmol; 1 mM) were dissolved in TFA (60 µL). Diphenylsulfoxide (0.120 mg; 0.6 µmol; 10 mM) was added to the mixture and the reaction was left to proceed at room temperature. The reaction was monitored by UPLC-MS through sampling of 5 µL aliquots which were quenched by addition of 0.2 M, pH 7.2 phosphate buffer (50 µL) before injection.

Only traces of the target product **6a** were observed accompanied by several intermediates and byproducts (Figure S27).

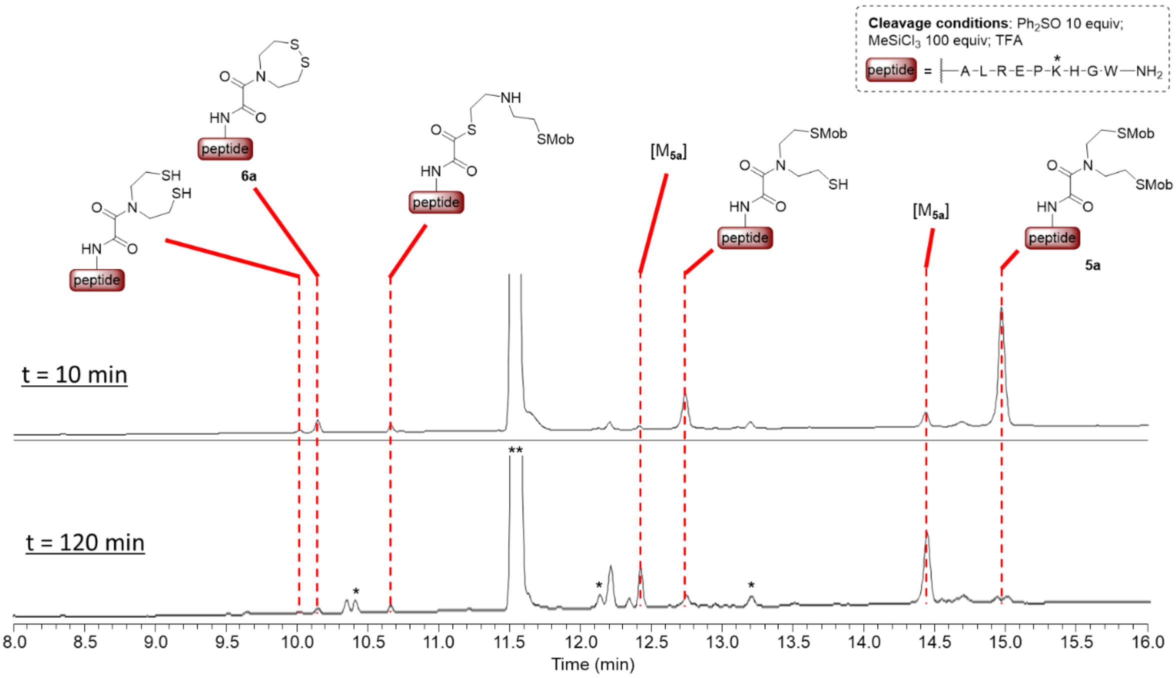

Figure S27. UPLC chromatograms of the monitoring at 215 nm of the Mob cleavage of peptide **5a** (ALREPK(^{oxo}SEA-Mob)HGW-NH$_2$) (Cleavage conditions: Ph$_2$SO 10 equiv; MeSiCl$_3$ 100 equiv; TFA) (LC trace. Eluent A 0.1% TFA in water, eluent B 0.1% TFA in CH$_3$CN. XBridge BEH C18 (3.5 µm, 300 Å, 2.1 × 150 mm), gradient 0-50% B in 15 min (0.4 mL min^{-1}, detection UV 215 nm)). *Non-peptidic impurity. **Ph$_2$SO.

Mob cleavage using the diphenylsulfoxide trichloromethylsilane system with catalytic thiophenol in TFA

Trichloromethylsilane (0.94 µL; 8 µmol; 100 mM), thiophenol (0.82 µL; 0.008 µmol; 0.1 mM or 4.1 µL; 0.04 µmol; 0.5 mM) and peptide **5a** (0.150 mg; 0.08 µmol; 1 mM) were dissolved in TFA (80 µL). Diphenylsulfoxide (0.160 mg; 0.8 µmol; 10 mM) was added to the mixture and the reaction was left to proceed at room temperature. The reaction was monitored by UPLC-MS through sampling of 5 µL aliquots which were quenched by addition of 0.2 M, pH 7.2 phosphate buffer (50 µL) before injection.

Only traces of the target product **6a** were observed accompanied by several intermediates and byproducts (Figure S28 and Figure S29).

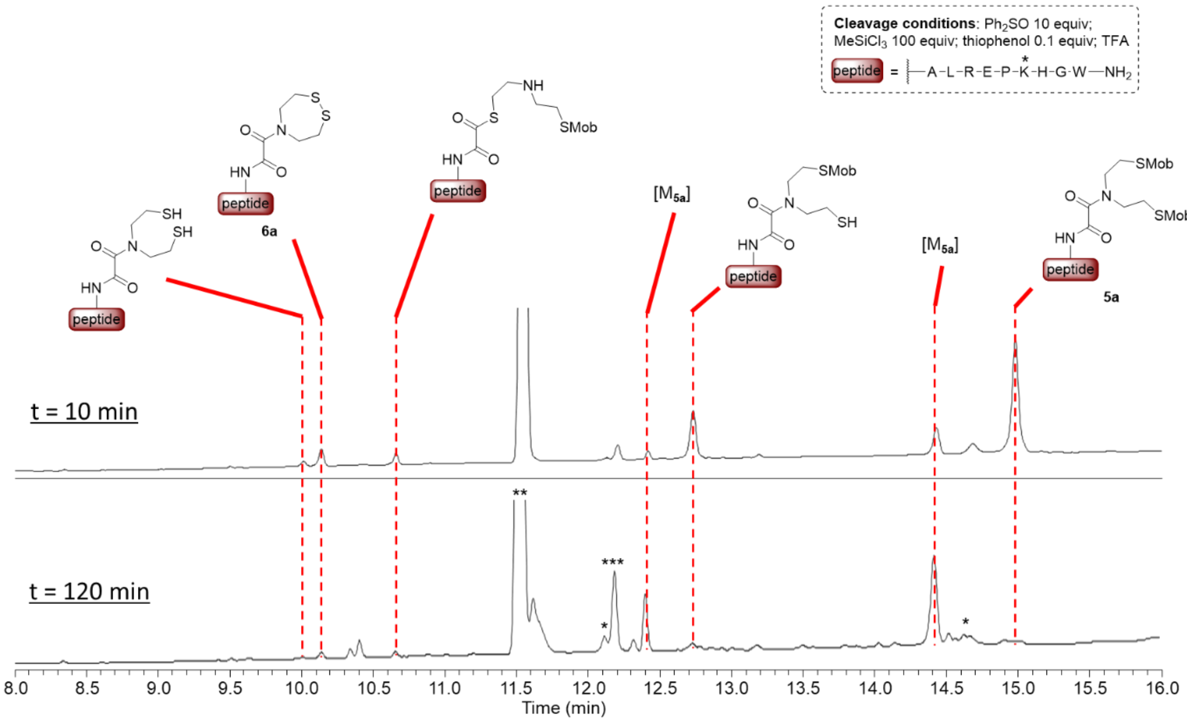

Figure S28. UPLC chromatograms of the monitoring at 215 nm of the Mob cleavage of peptide **5a** (ALREPK(^{oxo}SEA-Mob)HGW-NH$_2$) (Cleavage conditions: Ph$_2$SO 10 equiv; MeSiCl$_3$ 100 equiv; thiophenol 0.1 equiv; TFA) (LC trace. Eluent A 0.1% TFA in water, eluent B 0.1% TFA in CH$_3$CN. XBridge BEH C18 (3.5 µm, 300 Å, 2.1 × 150 mm), gradient 0-50% B in 15 min (0.4 mL min^{-1}, detection UV 215 nm)). *Non-peptidic impurity. **Ph$_2$SO. ***Uncharacterized peptidic material.

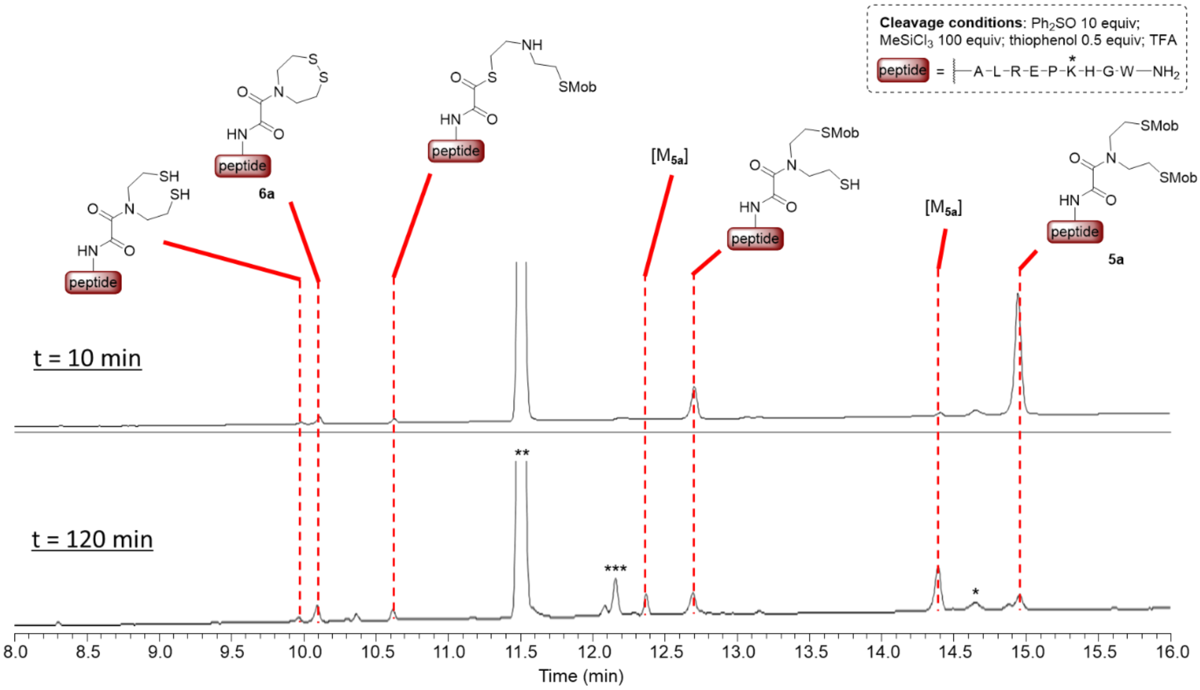

Figure S29. UPLC chromatograms of the monitoring at 215 nm of the Mob cleavage of peptide **5a** (ALREPK(^{oxo}SEA-Mob)HGW-NH$_2$) (Cleavage conditions: Ph_2SO 10 equiv; $MeSiCl_3$ 100 equiv; thiophenol 0.5 equiv; TFA) (LC trace. Eluent A 0.1% TFA in water, eluent B 0.1% TFA in CH_3CN. XBridge BEH C18 (3.5 µm, 300 Å, 2.1 × 150 mm), gradient 0-50% B in 15 min (0.4 mL min^{-1}, detection UV 215 nm)). *Non-peptidic impurity. **Ph_2SO. ***Uncharacterized peptidic material.

Mob cleavage using the diphenylsulfoxide trichloromethylsilane system in a TFA/TIS mixture

Trichloromethylsilane (1.63 µL; 14 µmol; 100 mM) and peptide **5a** (0.252 mg; 0.14 µmol; 1 mM) were dissolved in a TFA/TIS 95/5 v/v mixture (140 µL). Diphenylsulfoxide (0.280 mg; 1.4 µmol; 10 mM) was added to the mixture and the reaction was left to proceed at room temperature. The reaction was monitored by UPLC-MS through sampling of 10 µL aliquots which were quenched by addition of 0.2 M, pH 7.2 phosphate buffer (100 µL) before injection.

Efficient formation of the target product **6a** was observed but accompanied by several byproducts (Figure S30).

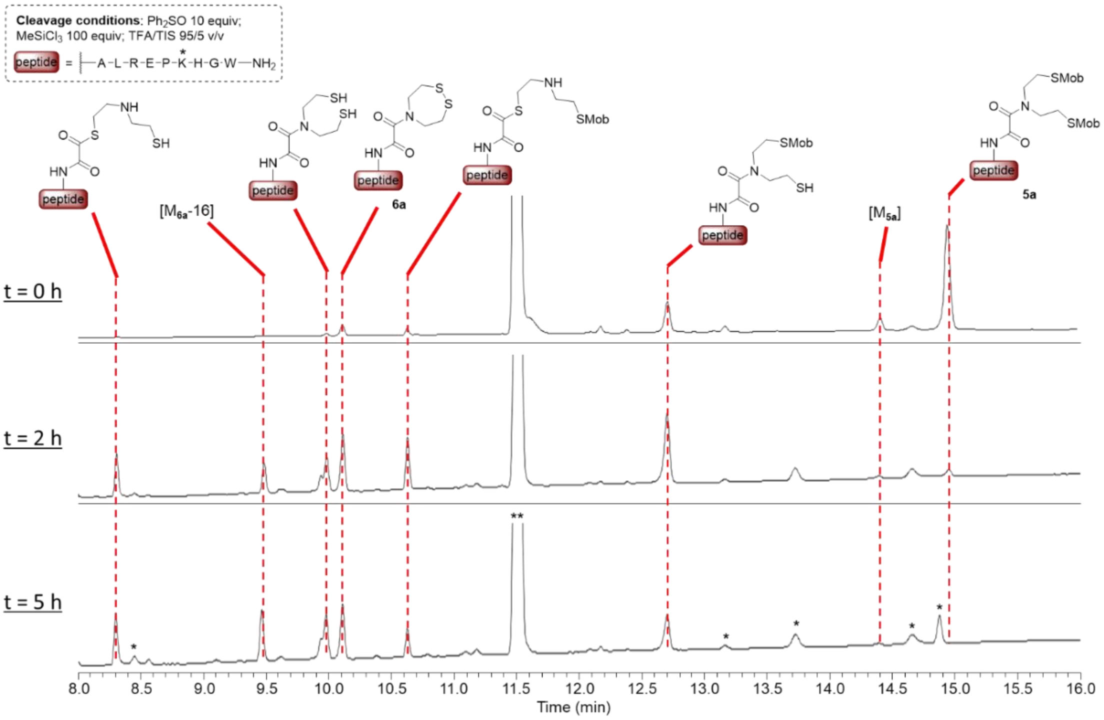

Figure S30. UPLC chromatograms of the monitoring at 215 nm of the Mob cleavage of peptide **5a** (ALREPK(^{oxo}SEA-Mob)HGW-NH$_2$) (Cleavage conditions: Ph_2SO 10 equiv; $MeSiCl_3$ 100 equiv; TFA/TIS 95/5 v/v) (LC trace. Eluent A 0.1% TFA in water, eluent B 0.1% TFA in CH_3CN. XBridge BEH C18 (3.5 µm, 300 Å, 2.1 × 150 mm), gradient 0-50% B in 15 min (0.4 mL min^{-1}, detection UV 215 nm)). *Non-peptidic impurity. **Ph_2SO.

Since most of the byproducts were reduced forms of the target product, we decided to perform an oxidative treatment. Therefore, reaction was monitored by UPLC-MS through sampling of 10 µL aliquots which were poured into a 100 µL solution of 4-mercaptophenyl acetic acid $(MPAA)_2$ disulfide (0.167 mg; 0.5 µmol; 50 equiv) in 0.2 M, pH 7.2 phosphate buffer. The pH of the solution was adjusted to 7 by addition of NaOH 6 M and oxidation was left to proceed during 15 minutes. The pH of the solution was adjusted to 2 by addition of HCl 6 M and the mixture was extracted with diethyl ether (3 × 500 µL) before injection.

Most byproducts were converted to the target product **6a** (Figure S31).

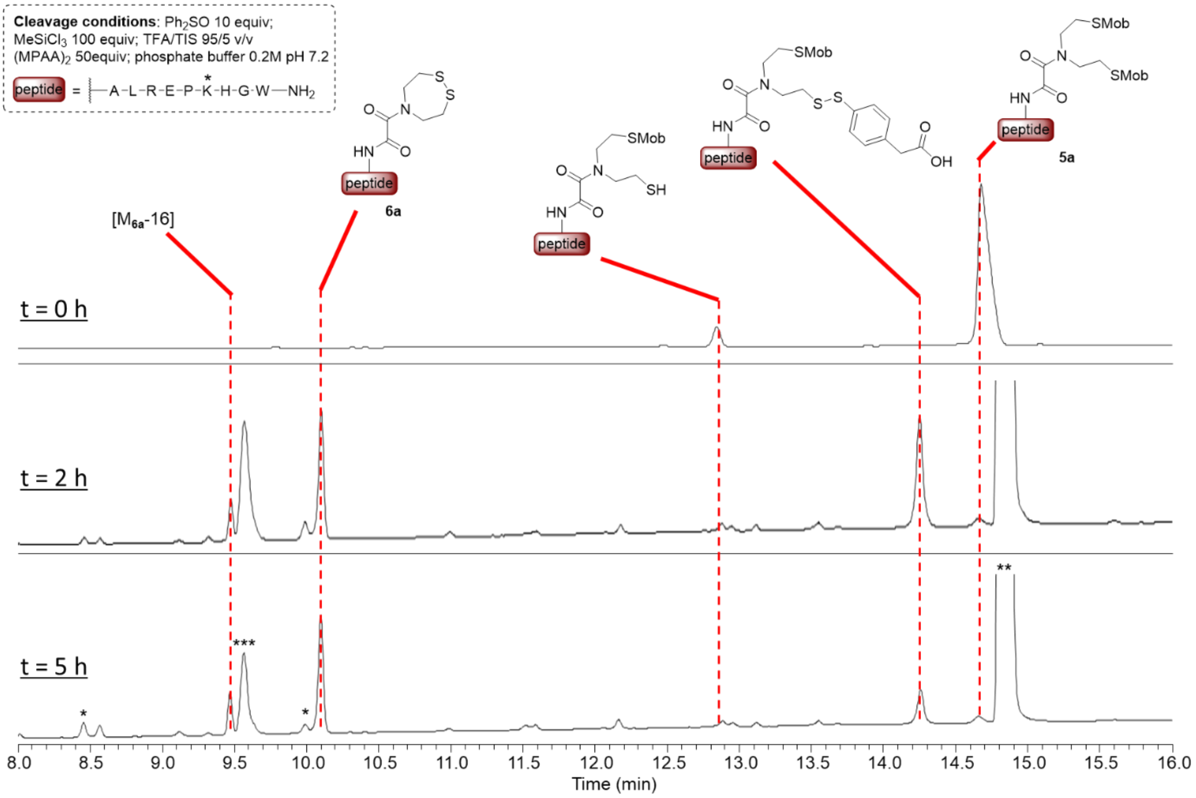

Figure S31. UPLC chromatograms of the monitoring at 215 nm of the Mob cleavage of peptide **5a** (ALREPK(^{oxo}SEA-Mob)HGW-NH_2) (Cleavage conditions: Ph_2SO 10 equiv; $MeSiCl_3$ 100 equiv; TFA/TIS 95/5 v/v; then $(MPAA)_2$ 50 equiv; phosphate buffer 0.2 M; pH 7.2) (LC trace. Eluent A 0.1% TFA in water, eluent B 0.1% TFA in CH_3CN. XBridge BEH C18 (3.5 µm, 300 Å, 2.1 × 150 mm), gradient 0-50% B in 15 min (0.4 mL min^{-1}, detection UV 215 nm)). *Non-peptidic impurity. **MPAA disulfide. ***MPAA.

Mob cleavage using the diphenylsulfoxide trichloromethylsilane system in a TFA/thioanisole mixture

Trichloromethylsilane (2.67 µL; 23 µmol; 100 mM) and peptide **5a** (0.430 mg; 0.23 µmol; 1 mM) were dissolved in a TFA/thioanisole 95/5 v/v mixture (230 µL). Diphenylsulfoxide (0.465 mg; 2.3 µmol; 10 mM) was added to the mixture and the reaction was left to proceed at room temperature. The reaction was monitored by UPLC-MS through sampling of 5 µL aliquots which were quenched by addition of 0.2 M, pH 7.2 phosphate buffer (50 µL) and then extracted with diethyl ether (3 × 100 µL) before injection.

Efficient formation of the target product **6a** was observed with only traces of byproducts (Figure S32).

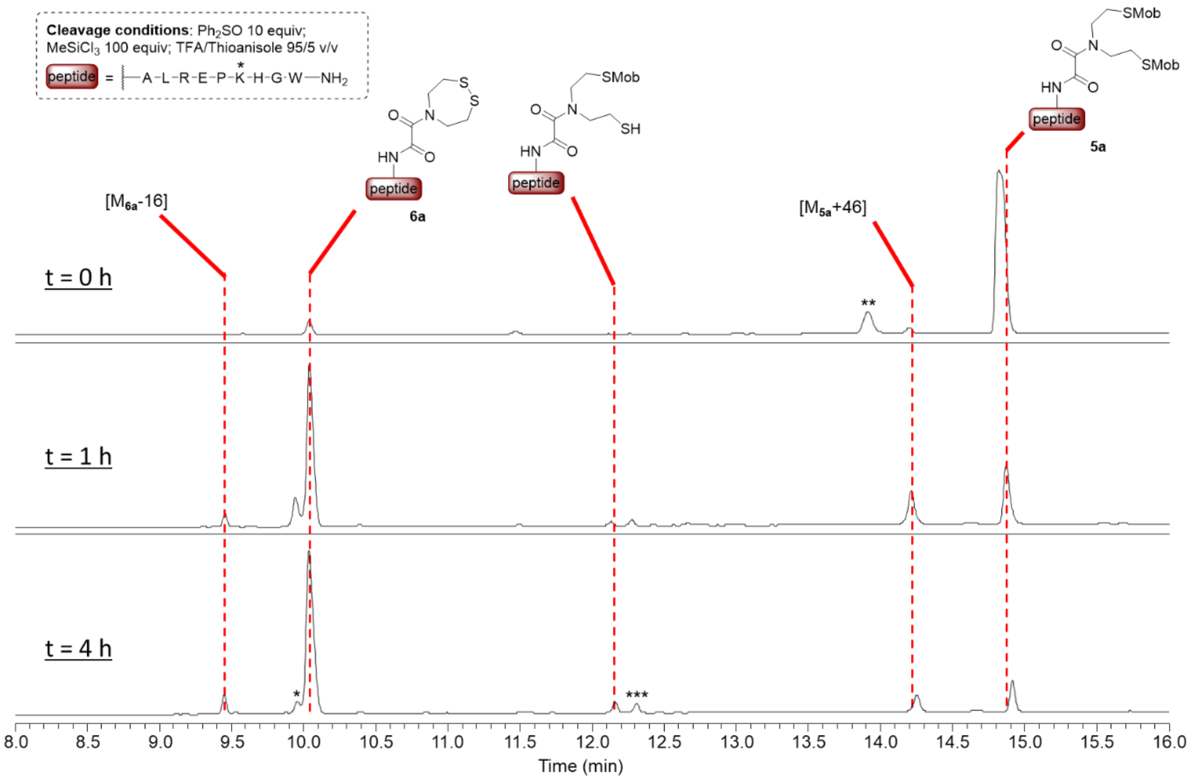

Figure S32. UPLC chromatograms of the monitoring at 215 nm of the Mob cleavage of peptide **5a** (ALREPK(oxoSEA-Mob)HGW-NH$_2$) (Cleavage conditions: Ph$_2$SO 10 equiv; MeSiCl$_3$ 100 equiv; TFA/thioanisole 95/5 v/v). (LC trace. Eluent A 0.1% TFA in water, eluent B 0.1% TFA in CH$_3$CN. XBridge BEH C18 (3.5 µm, 300 Å, 2.1 × 150 mm), gradient 0-50% B in 15 min (0.4 mL min^{-1}, detection UV 215 nm)). *Non-peptidic impurity. **Thioanisole. ***Uncharacterized peptidic material.

6. Suitability for Cys- and Met-containing peptides

Oxidizing conditions for the cleavage of Mob protecting groups were applied to peptide segments containing sensitive residues towards oxidation (cysteine or methionine) and the reaction was monitored by UPLC-MS (Figure S33).

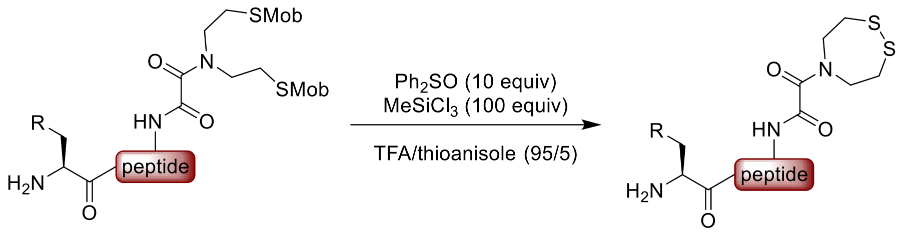

Figure S33. Procedure for the Mob cleavage of Met-peptide **5b** and Cys-peptide **5c**.

<u>Mob cleavage protocol – general procedure</u>

To 150 µL of a TFA/thioanisole mixture (95/5) was added trichloromethylsilane (1.74 µL; 15 µmol; 100 mM) and the peptide **5b** or **5c** (0.300 mg; 0.15 µmol; 1 mM). Diphenylsulfoxide (0.303 mg; 1.5 µmol; 10 mM) was added to the mixture and the reaction was left to proceed at room temperature. The reaction was monitored by UPLC-MS through sampling of 3 µL aliquots which were quenched by addition of 0.2 M, pH 7.2 phosphate buffer (30 µL) and then extracted with diethyl ether (3 × 100 µL) before injection.

<u>Mob cleavage of MALREPK(^{oxo}SEA-Mob)HGW-NH_2 peptide **5b**</u>

Cleavage of Mob groups was observed to be slower when a methionine residue is present in the sequence but still leading efficiently to the formation of the targeted product **5b**. No byproducts stemming from the oxidation of the methionine residue were observed.

MS (ESI, positive detection mode, Figure S34) m/z = 1412.67 ($[M+H]^+$), 707.08 ($[M+2H]^{2+}$), 471.83 ($[M+3H]^{3+}$). Calcd. for [M] (average): 1412.71, found: 1411.92.

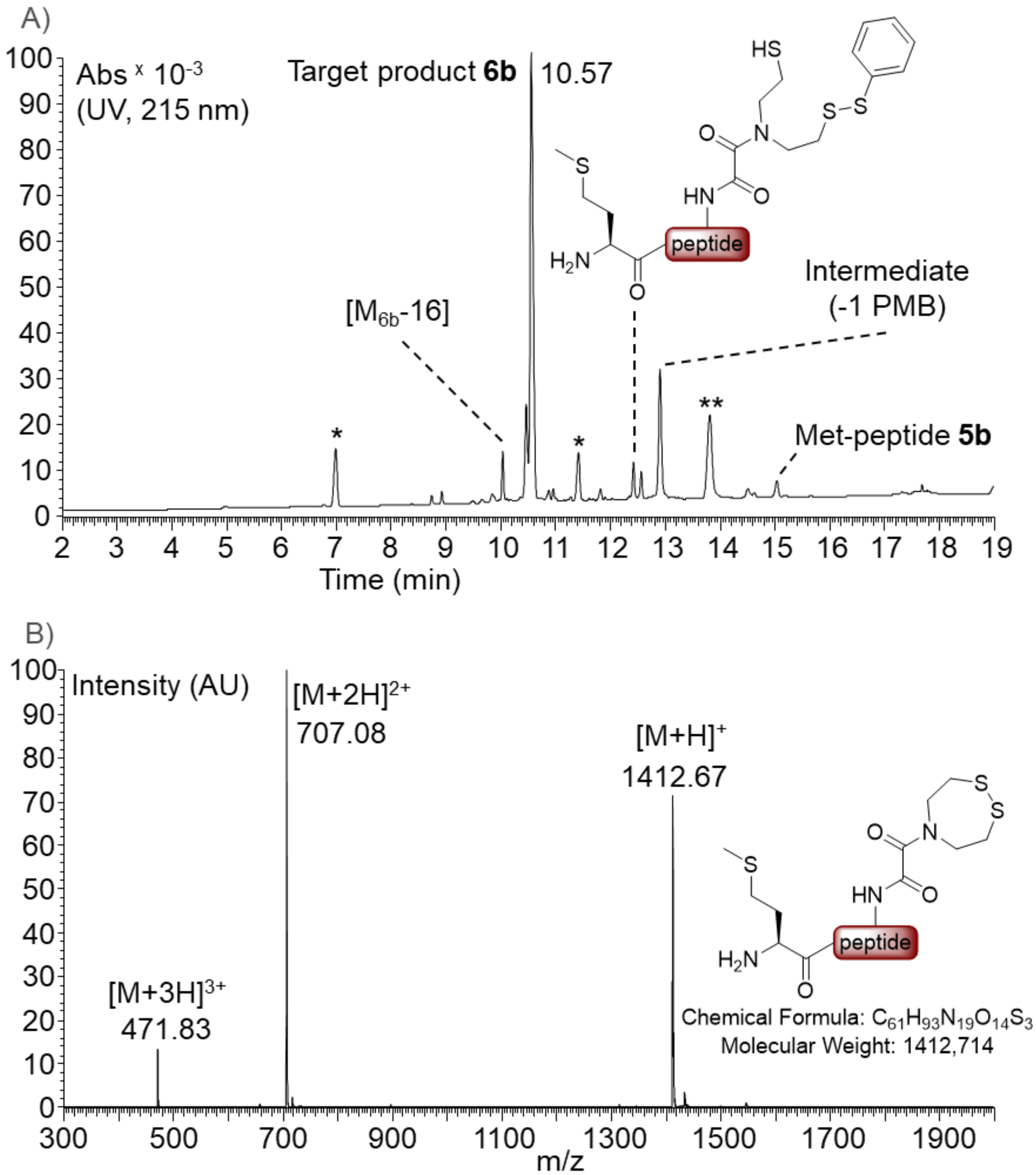

Figure S34. UPLC-MS analysis of the oxidative cleavage of Mob from MALREPK(^{oxo}SEA-Mob)HGW-NH_2 peptide **5b** after 4 hours of reaction. A) LC trace. Eluent A 0.1% TFA in water, eluent B 0.1% TFA in CH_3CN. XBridge BEH C18 (3.5 µm, 300 Å, 2.1 × 150 mm), gradient 0-50% B in 15 min (0.4 mL min^{-1}, detection UV 215 nm). B) MS trace at 10.57 min (compound **6b**): m/z = 1412.67 ([M+H]$^+$), 707.08 ([M+2H]$^{2+}$), 471.83 ([M+3H]$^{3+}$). Calcd. for [M] (average): 1412.71, found: 1411.92. *Non-peptidic impurity. **Thioanisole.

Mob cleavage of CALREPK(^{oxo}SEA-Mob)HGW-NH_2 peptide **5c**

Rapid and efficient cleavage of Mob groups was observed leading to the formation of the targeted product **6c**. No byproducts coming from oxidation of the cysteine residue were observed, however, formation of several byproducts stemming from side reactions employing the thiol function of the cysteine residue were detected.

MS (ESI, positive detection mode, Figure S35) m/z = 1384.67 ($[M+H]^+$), 693.08 ($[M+2H]^{2+}$), 462.33 ($[M+3H]^{3+}$). Calcd. for [M] (average): 1384.66, found: 1383.92.

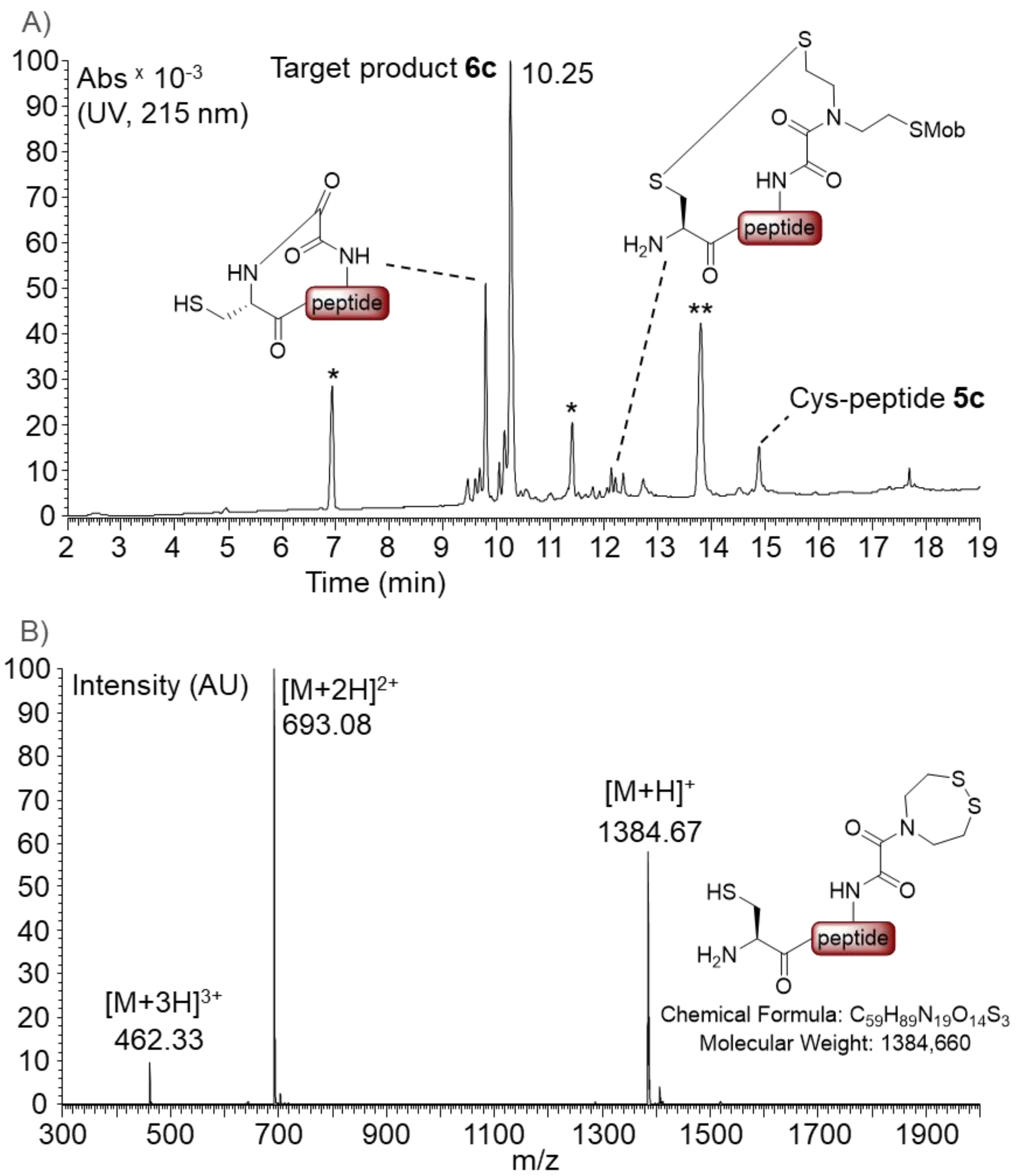

Figure S35. UPLC-MS analysis of the oxidative cleavage of Mob from CALREPK(^{oxo}SEA-Mob)HGW-NH_2 peptide **5c** after 1 hour of reaction. A) LC trace. Eluent A 0.1% TFA in water, eluent B 0.1% TFA in CH_3CN. XBridge BEH C18 (3.5 µm, 300 Å, 2.1 × 150 mm), gradient 0-50% B in 15 min (0.4 mL min^{-1}, detection UV 215 nm). B) MS trace at 10.25 min (compound **6c**): m/z = 1384.67 ([M+H]$^{+}$), 693.08 ([M+2H]$^{2+}$), 462.33 ([M+3H]$^{3+}$). Calcd. for [M] (average): 1384.66, found: 1383.92. *Non-peptidic impurity. **Thioanisole.

7. Synthesis and characterization of ^{oxo}SEA-containing polypeptide

Synthesis

Synthesis and characterization of ILKEPVHGACALREPK(^{oxo}SEA-Mob)HGW-NH_2 peptide **9a**

Ligated peptide **9a** (ILKEPVHGACALREPK(^{oxo}SEA-Mob)HGW-NH_2) was synthesized from peptide **5c** CALREPK(^{oxo}SEA-Mob)HGW-NH_2 according to two different procedures (Figure S36):

- Native Chemical Ligation (NCL) with thioester peptide **16** (ILKEPVHGA-MPA)
- SEA-mediated ligation with thioester peptide **7a** (ILKEPVHGA-SEA)

A)

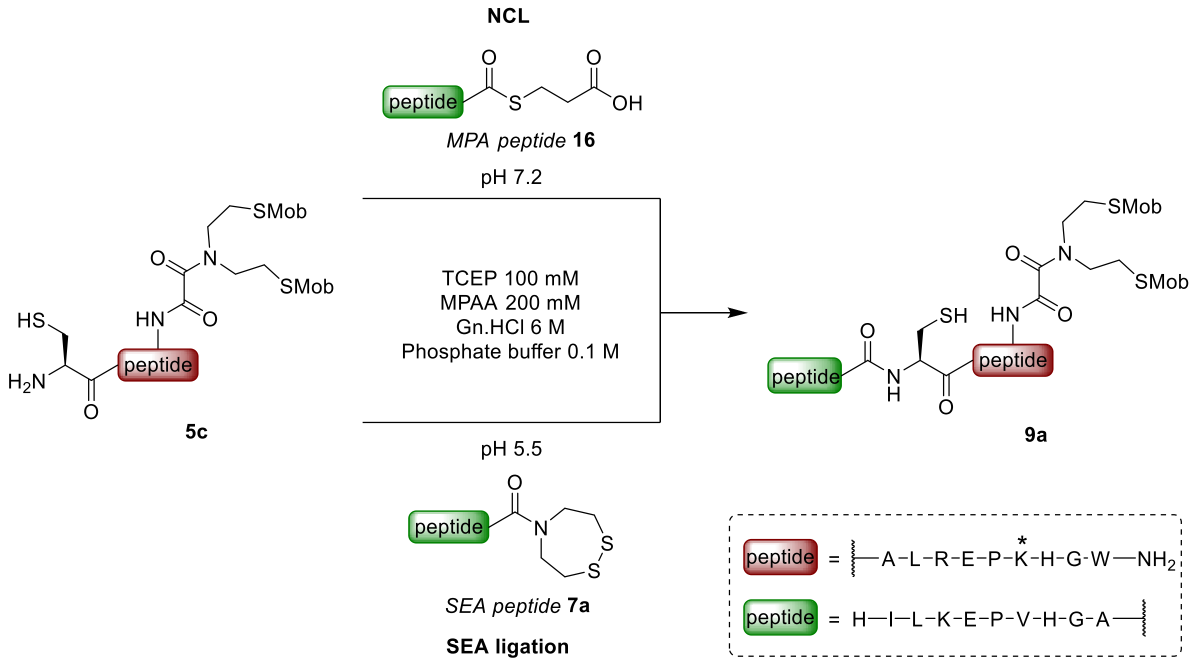

B)

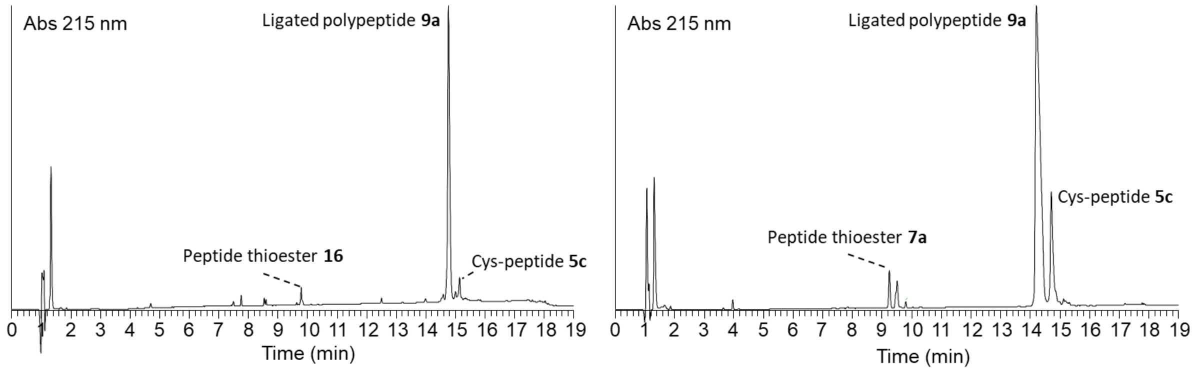

Figure S36. A) Procedures for the synthesis of ^{oxo}SEA polypeptide **9a**. B) UPLC analysis of the formation of ILKEPVHGACALREPK(^{oxo}SEA-Mob)HGW-NH_2 peptide **9a** according to NCL reaction (left) and SEA-mediated ligation reaction (right) after overnight reaction (Eluent A 0.1% TFA in water, eluent B 0.1% TFA in CH_3CN. XBridge BEH C18 (3.5 µm, 300 Å, 2.1 × 150 mm), gradient 0-50% B in 15 min (0.4 mL min^{-1}, detection UV 215 nm)).

Via NCL reaction

To a solution of Gn·HCl (573 mg) in 0.1 M, pH 7.2 phosphate buffer (600 µL) were added TCEP (28.7 mg; 100 µmol; 100 mM) and MPAA (33.6 mg; 200 µmol; 200 mM). The pH of the mixture was adjusted to 7.2 by addition of NaOH 6 M. The Cys-peptide **5c** (2.0 mg; 1.02 µmol; 3.6 mM) was dissolved in 283 µL of the above solution. The resulting mixture (283 µL) was added on the thioester peptide **16** (1.2 mg; 0.85 µmol; 3 mM) and the reaction was stirred overnight at 37°C and under nitrogen atmosphere. The mixture was quenched by addition of glacial acetic acid (28 µL) and extracted with diethyl ether (4 × 500 µL). Purification of the crude was performed by preparative RP-HPLC using a preparative C18 XBridge BEH300 column (5 µm, 300 Å, 10 × 250 mm, 50 °C, 215 nm, 6 mL min^{-1}, eluent A: 0.1% by vol. of TFA in water, eluent B: 0.1% by vol. of TFA in acetonitrile, 0-30% B in 10 min, then 30-55% B in 50 min). The purified fractions were combined and lyophilized to give the title peptide as a white solid (1.67 mg, 74%).

MS (ESI, positive detection mode, Figure S37) m/z = 1286.75 ($[M+2H]^{2+}$), 858.33 ($[M+3H]^{3+}$), 644.00 ($[M+4H]^{4+}$). Calcd. for [M] (average): 2572.11, found: 2571.75.

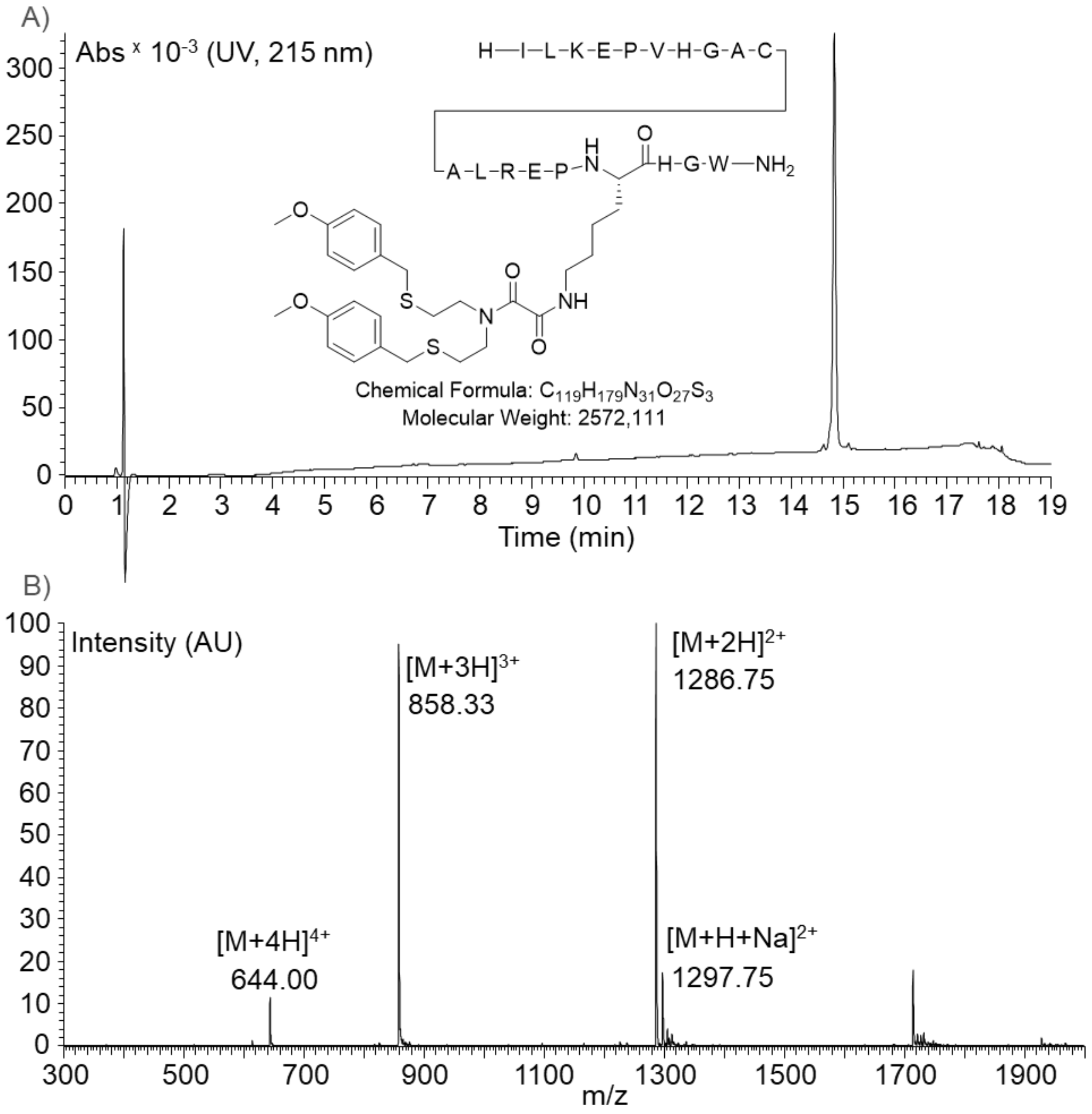

Figure S37. UPLC-MS analysis of ILKEPVHGACALREPK(^{oxo}SEA-Mob)HGW-NH_2 peptide **9a**. A) LC trace. Eluent A 0.1% TFA in water, eluent B 0.1% TFA in CH_3CN. XBridge BEH C18 (3.5 µm, 300 Å, 2.1 × 150 mm), gradient 0-50% B in 15 min (0.4 mL min^{-1}, detection UV 215 nm). B) MS trace: m/z = 1286.75 ($[M+2H]^{2+}$), 858.33 ($[M+3H]^{3+}$), 644.00 ($[M+4H]^{4+}$). Calcd. for [M] (average): 2572.11, found: 2571.75.

Via SEA-mediated ligation reaction

To a solution of Gn·HCl (1.15 g) in 0.1 M, pH 7.2 phosphate buffer (1.2 mL) were added TCEP (57.4 mg; 200 µmol; 100 mM) and MPAA (67.2 mg; 400 µmol; 200 mM). The pH of the mixture was adjusted to 5.5 by addition of NaOH 6 M. The Cys-peptide **5c** (10.0 mg; 5.08 µmol; 3.6 mM) was dissolved in 1.41 mL of the above solution. The resulting mixture (1.41 mL) was added on the thioester peptide **7a** (6.0 mg; 4.23 µmol; 3 mM) and the reaction was stirred overnight at 37°C and under nitrogen atmosphere. The mixture was quenched by addition of glacial acetic acid (141 µL) and the mixture was extracted with diethyl ether (4 × 2 mL). Purification of the crude was performed by preparative RP-HPLC using a preparative C18 XBridge BEH300 column (5 µm, 300 Å, 10 × 250 mm, 50 °C, 215 nm, 6 mL min^{-1}, eluent A: 0.1% by vol. of TFA in water, eluent B: 0.1% by vol. of TFA in acetonitrile, 0-30% B in 10 min, then 30-55% B in 50 min). The purified fractions were combined and lyophilized to give the title peptide as a white solid (9.9 mg, 75%).

MS (ESI, positive detection mode, Figure S38) m/z = 1286.67 ($[M+2H]^{2+}$), 858.25 ($[M+3H]^{3+}$), 644.08 ($[M+4H]^{4+}$). Calcd. for [M] (average): 2572.11, found: 2571.55.

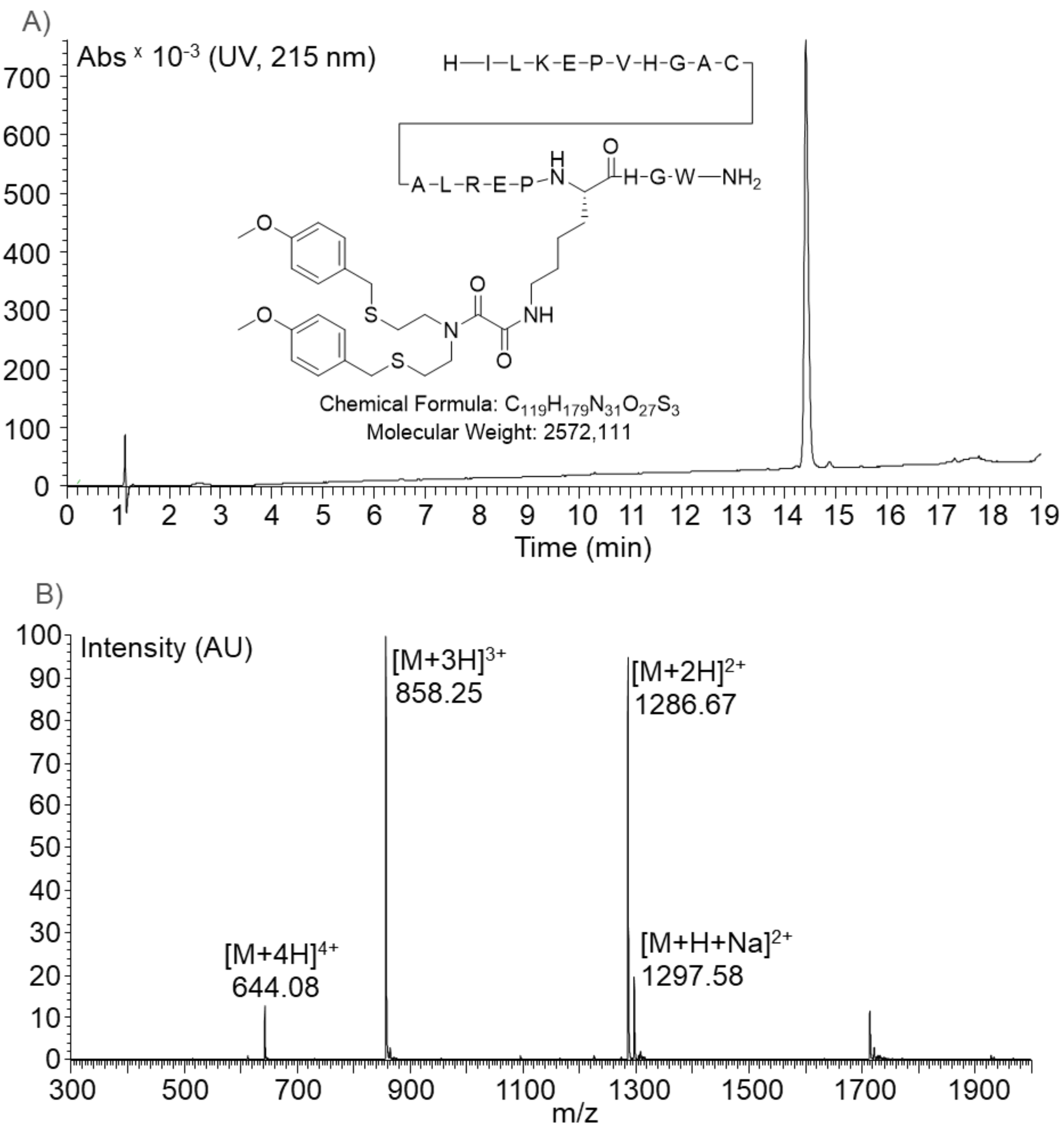

Figure S38. UPLC-MS analysis of ILKEPVHGACALREPK(oxoSEA-Mob)HGW-NH_2 peptide **9a**. A) LC trace. Eluent A 0.1% TFA in water, eluent B 0.1% TFA in CH_3CN. XBridge BEH C18 (3.5 µm, 300 Å, 2.1 × 150 mm), gradient 0-50% B in 15 min (0.4 mL min^{-1}, detection UV 215 nm). B) MS trace: m/z = 1286.67 ([M+2H]$^{2+}$), 858.25 ([M+3H]$^{3+}$), 644.08 ([M+4H]$^{4+}$). Calcd. for [M] (average): 2572.11, found: 2571.55.

Synthesis and characterization of ILKEPVHGAAALREPK(^{oxo}SEA-Mob)HGW-NH_2 peptide **10a**

To a solution of Gn·HCl (1.72 g) in 0.1 M, pH 7.2 phosphate buffer (1.8 mL) were added TCEP (172.0 mg; 600 µmol; 200 mM), GSH (46.1 mg; 150 µmol; 50 mM) and VA-044 (19.4 mg; 60 µmol; 20 mM). The pH of the mixture was adjusted to 6.5 by addition of NaOH 6 M. The polypeptide **9a** (8.0 mg; 2.55 µmol; 1 mM) was dissolved in 2.55 mL of the above solution and the reaction was stirred during 3 hours at 37°C. Purification of the crude was performed by preparative RP-HPLC using a preparative C18 XBridge BEH300 column (5 µm, 300 Å, 10 × 250 mm, 50 °C, 215 nm, 6 mL min^{-1}, eluent A: 0.1% by vol. of TFA in water, eluent B: 0.1% by vol. of TFA in acetonitrile, 0-30% B in 10 min, then 30-55% B in 50 min). The purified fractions were combined and lyophilized to give the title peptide as a white solid (4.14 mg, 52%).

MS (ESI, positive detection mode, Figure S39) m/z = 1270.67 ($[M+2H]^{2+}$), 847.67 ($[M+3H]^{3+}$), 636.00 ($[M+4H]^{4+}$). Calcd. for [M] (average): 2540.05, found: 2539.68.

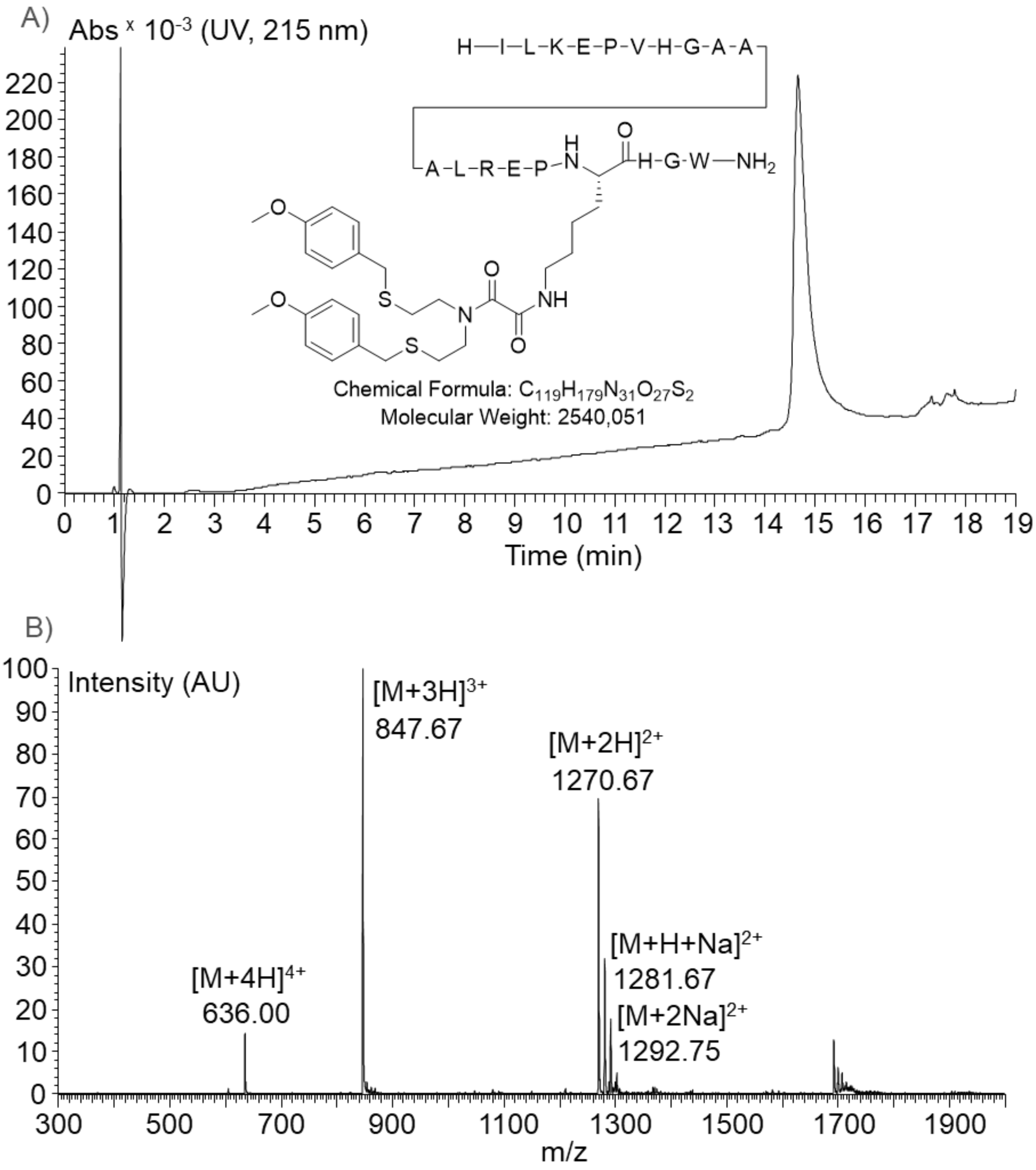

Figure S39. UPLC-MS analysis of ILKEPVHGAAALREPK(^{oxo}SEA-Mob)HGW-NH_2 peptide **10a**. A) LC trace. Eluent A 0.1% TFA in water, eluent B 0.1% TFA in CH_3CN. XBridge BEH C18 (3.5 µm, 300 Å, 2.1 × 150 mm), gradient 0-50% B in 15 min (0.4 mL min^{-1}, detection UV 215 nm). B) MS trace: m/z = 1270.67 ($[M+2H]^{2+}$), 847.67 ($[M+3H]^{3+}$), 636.00 ($[M+4H]^{4+}$). Calcd. for [M] (average): 2540.05, found: 2539.68.

Synthesis and characterization of ILKEPVHGAAALREPK(^{oxo}SEA)HGW-NH$_2$ peptide **11a**

To 680 µL of a TFA/thioanisole mixture (95/5) was added trichloromethylsilane (7.88 µL; 68 µmol; 100 mM) and the polypeptide **10a** (2.10 mg; 0.68 µmol; 1 mM). Diphenylsulfoxide (1.38 mg; 6.8 µmol; 10 mM) was added to the mixture and the reaction was stirred during 20 minutes at room temperature. Reaction was monitored by UPLC-MS through sampling of 2 µL aliquots which were quenched by addition of 0.2 M, pH 7.2 phosphate buffer (20 µL) and then extracted with diethyl ether (3 × 100 µL) before injection (Figure S40).

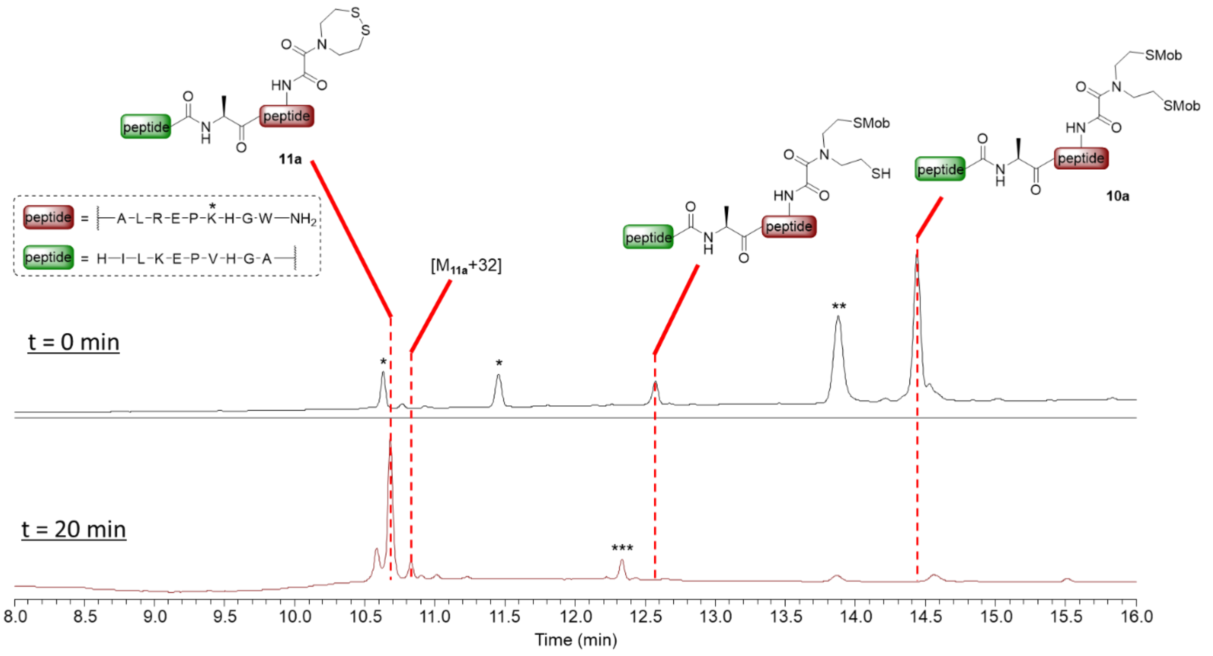

Figure S40. UPLC chromatograms of the monitoring at 215 nm of the Mob cleavage of peptide **10a** (ILKEPVHGAAALREPK(^{oxo}SEA-Mob)HGW-NH$_2$) (peptide **10a** 1 mM; diphenylsulfoxide 10 mM; trichloromethylsilane 100 mM; TFA/thioanisole 95/5 v/v) (LC trace. Eluent A 0.1% TFA in water, eluent B 0.1% TFA in CH_3CN. XBridge BEH C18 (3.5 µm, 300 Å, 2.1 × 150 mm), gradient 0-50% B in 15 min (0.4 mL min^{-1}, detection UV 215 nm)). *Non-peptidic impurity. **Thioanisole. ***Uncharacterized peptidic material.

The mixture was quenched by addition of 0.2 M, pH 7.2 phosphate buffer (3.5 mL) and the mixture was extracted with diethyl ether (4 × 5 mL). Purification of the crude was performed by preparative RP-HPLC using a preparative C18 XBridge BEH300 column (5 µM, 300 Å, 10 × 250 mm, 50 °C, 215 nm, 6 mL min^{-1}, eluent A: 0.1% by vol. of TFA in water, eluent B: 0.1% by vol. of TFA in acetonitrile, 0-20% B in 10 min, then 20-45% B in 45 min). The purified

fractions were combined and lyophilized to give the title peptide as a white solid (0.29 mg, 15%).

MS (ESI, positive detection mode, Figure S41) m/z = 1149.58 ($[M+2H]^{2+}$), 766.83 ($[M+3H]^{3+}$), 575.33 ($[M+4H]^{4+}$). Calcd. for [M] (average): 2297.73, found: 2297.33.

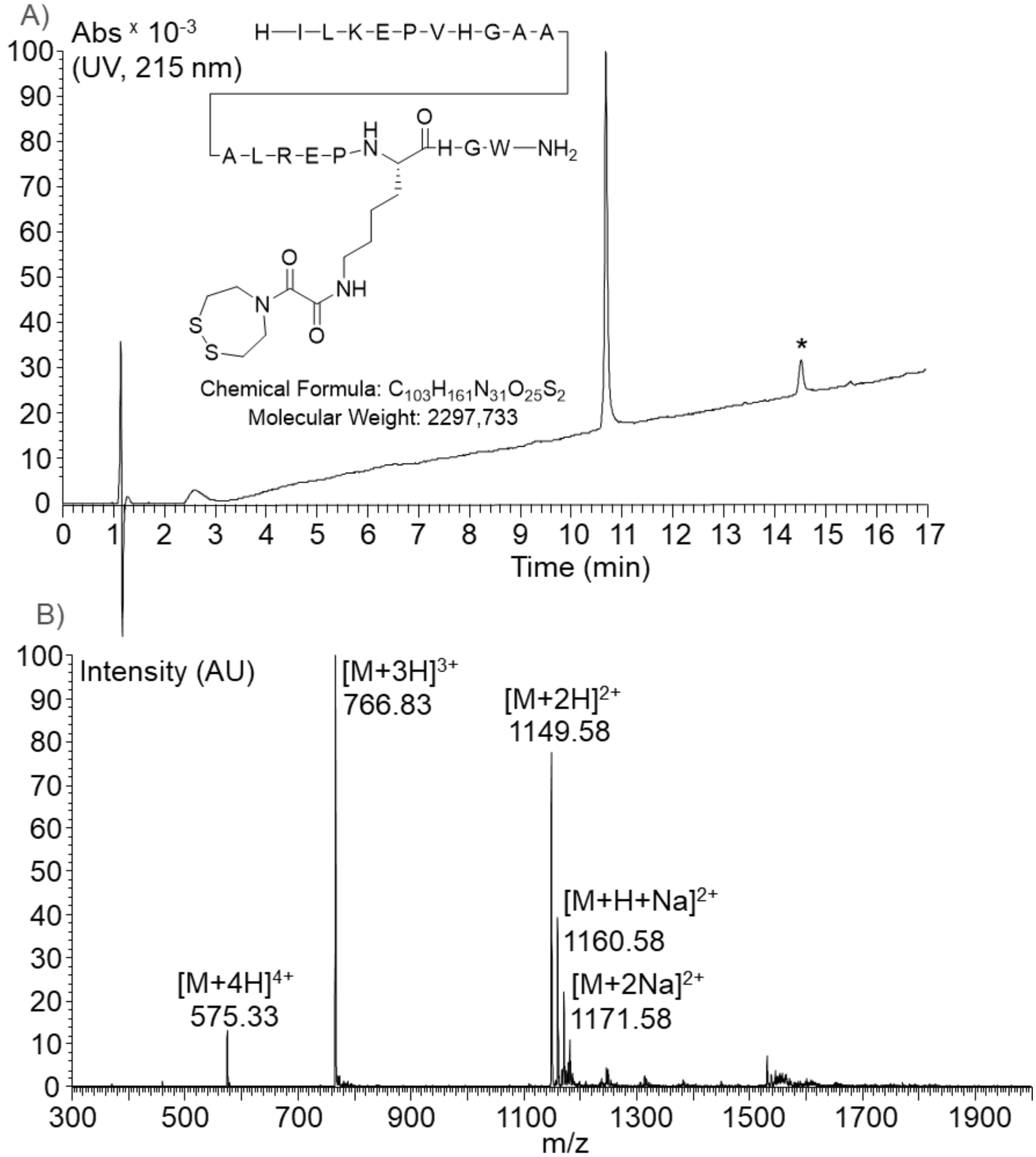

Figure S41. UPLC-MS analysis of ILKEPVHGAAALREPK(^{oxo}SEA)HGW-NH_2 peptide **11a**. A) LC trace. Eluent A 0.1% TFA in water, eluent B 0.1% TFA in CH_3CN. XBridge BEH C18 (3.5 µm, 300 Å, 2.1 × 150 mm), gradient 0-50% B in 15 min (0.4 mL min^{-1}, detection UV 215 nm). B) MS trace: m/z = 1149.58 ($[M+2H]^{2+}$), 766.83 ($[M+3H]^{3+}$), 575.33 ($[M+4H]^{4+}$). Calcd. for [M] (average): 2297.73, found: 2297.33. *Non-peptidic impurity.

^{oxo}SEA reactivity validation

The ^{oxo}SEA polypeptide **11a** (ILKEPVHGAAALREPK(^{oxo}SEA)HGW-NH_2) was involved in a Fast-SEA ligation reaction with Cys-peptide **12** (CILKEPVHGA-NH_2) according to standard ligation conditions and the reaction was monitored by analytical RP-HPLC (Figure S42).

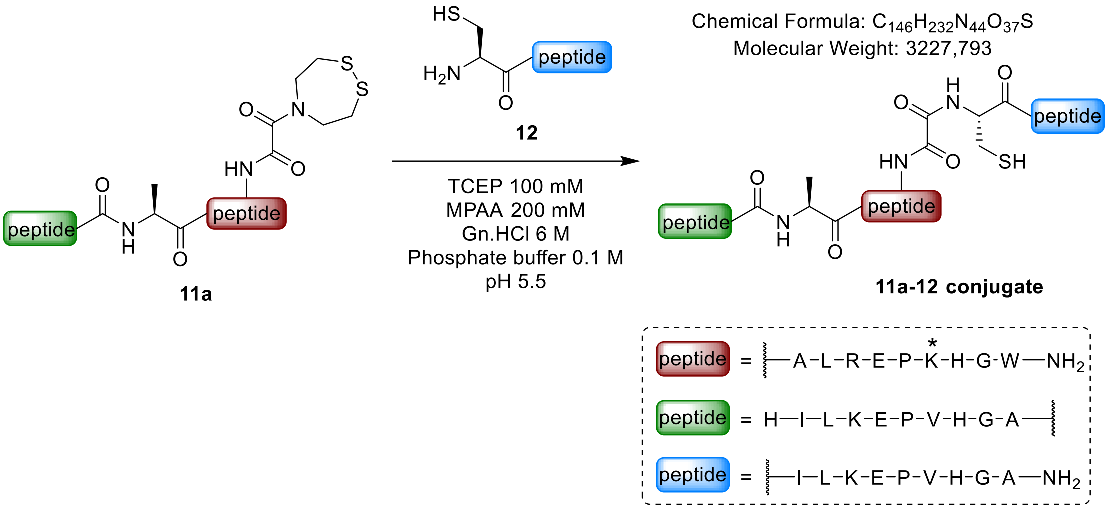

Figure S42. Synthetic scheme for the Fast-SEA ligation reaction between the ^{oxo}SEA polypeptide **11a** (ILKEPVHGAAALREPK(^{oxo}SEA)HGW-NH_2) and the Cys-peptide **12** (CILKEPVHGA-NH_2).

<u>Protocol</u>

To a solution of Gn·HCl (573 mg) in 0.1 M, pH 7.2 phosphate buffer (600 µL) were added TCEP (28.7 mg; 100 µmol; 100 mM) and MPAA (33.6 mg; 200 µmol; 200 mM). The pH of the mixture was adjusted to 5.5 by addition of NaOH 6 M. The Cys-peptide **12** (0.436 mg; 0.31 µmol; 0.6 mM) was dissolved in 520 µL of the above solution. The resulting mixture (520 µL) was added on the ^{oxo}SEA polypeptide **11a** (0.750 mg; 0.26 µmol; 0.5 mM) and the reaction was left to proceed at 37°C and under nitrogen atmosphere. The reaction was monitored by RP-HPLC through sampling of 20 µL aliquots which were quenched by addition of acetic acid 10% v/v in water (100 µL) and then extracted with diethyl ether (4 × 300 µL) before injection. Identification of ligation intermediates and products was ensured by UPLC-MS analysis.

Kinetic monitoring

Conversion to **11a-12 conjugate** was calculated from the UV trace at 280 nm (Trp residue absorption). Conversions were transformed into concentrations of the product based on the starting ^{oxo}SEA polypeptide **11a** concentration (Figure S43).

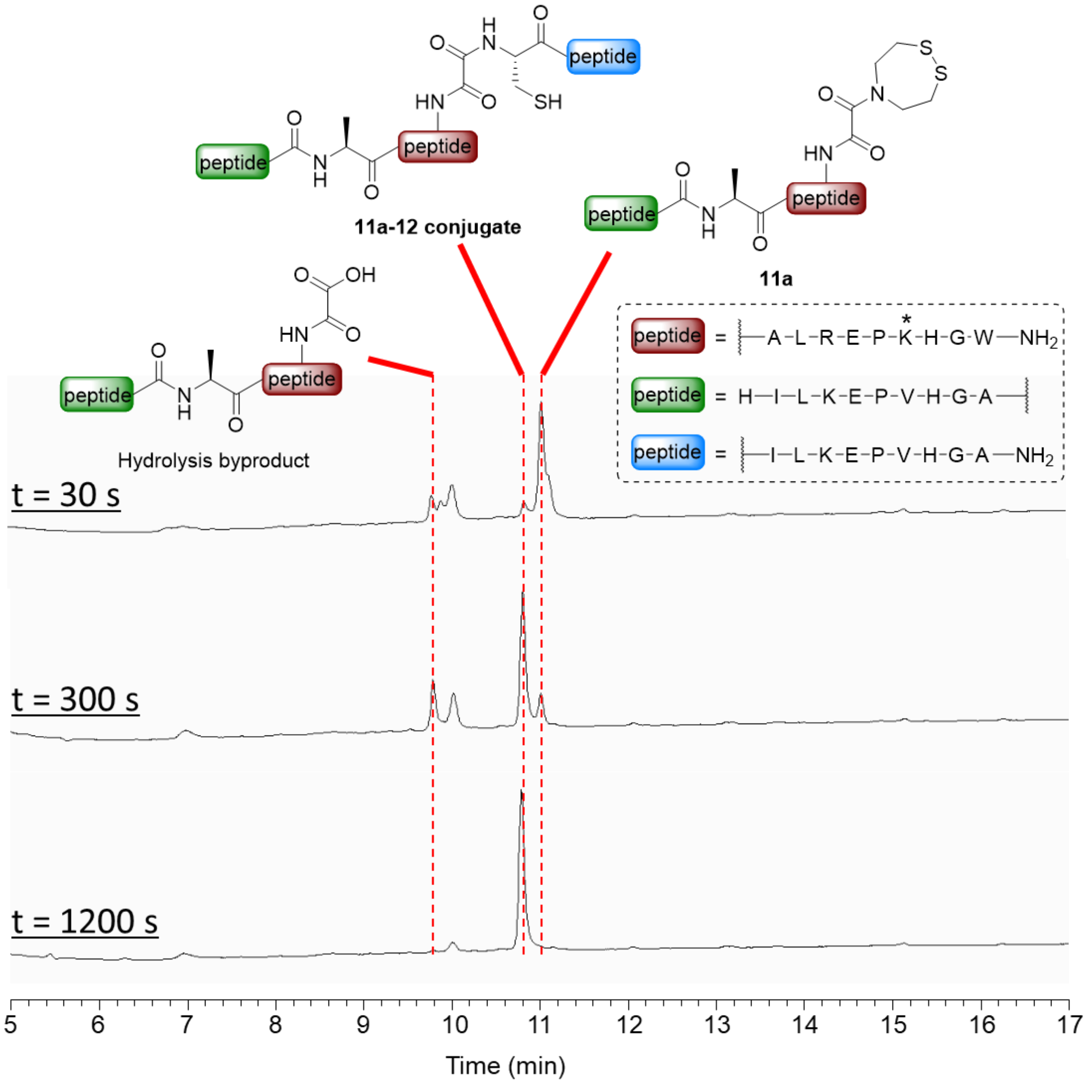

Figure S43. Examples of RP-HPLC chromatograms of the monitoring at 280 nm of the Fast-SEA ligation presented in Figure S42 performed at 500 µM **11a** concentration (^{oxo}SEA polypeptide **11a** 500 µM; Cys-peptide **12** 600 µM; TCEP 100 mM; MPAA 200 mM; 6 M Gn·HCl in 0.1 M phosphate buffer; pH 5.5; 37 °C) (Eluent A 0.1% TFA in water, eluent B 0.1% TFA in CH_3CN. XBridge BEH C18 (3.5 µm, 300 Å, 4.6 × 150 mm), gradient 0-50% B in 15 min (1.0 mL min^{-1}, detection UV 280 nm).

Conversion rate of the Fast-SEA ligation performed with ^{oxo}SEA polypeptide **11a** proceeded at a rate in agreement with previous work[3] (Figure S44).

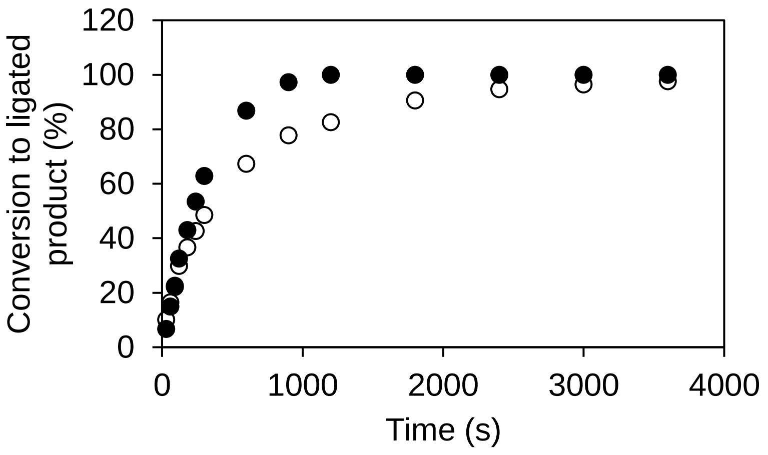

Figure S44. RP-HPLC monitoring of the Fast-SEA ligation presented in Figure S42 performed at 500 µM **11a** concentration (^{oxo}SEA polypeptide **11a** 500 µM; Cys-peptide **12** 600 µM; TCEP 100 mM; MPAA 200 mM; 6 M Gn·HCl in 0.1 M phosphate buffer; pH 5.5; 37 °C). Black dots correspond to the formation of **11a-12** conjugate. White dots correspond to data obtained from previous work.

8. Synthesis and characterization of Ubiquitin(^{oxo}SEA) protein

General synthetic scheme

The following scheme describes the synthesis of Ubiquitin(^{oxo}SEA) **11b**, an analogue of the ubiquitin presenting the ^{oxo}SEA group on the side chain of a lysine residue. The starting peptide segments were synthesized by SPPS according to standard protocols using a SEA PS solid support for segment **7b** and a Wang solid support for segment **8**. A cysteine residue was introduced on the *N*-terminal position of segment **8**. A SEA-mediated ligation of the two segments provided polypeptide **9b**. The latter was then submitted to a desulfurization reaction to restore the native alanine residue and yield Ubiquitin(^{oxo}SEA-Mob) **10b**. Finally, selective cleavage of Mob protecting groups resulted in the formation of the targeted Ubiquitin(^{oxo}SEA) **11b**.

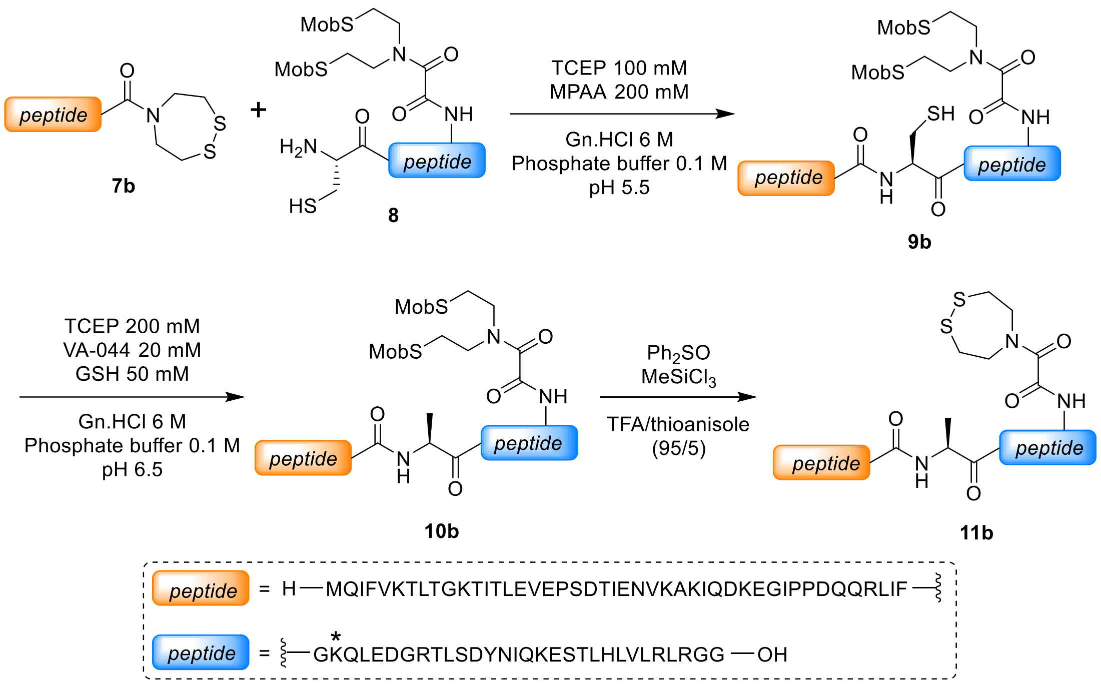

Figure S45. General synthetic scheme for the synthesis of K48 ^{oxo}SEA-ubiquitin **11b**.

Protocols

<u>Synthesis and characterization of **7b** segment[6]</u>

Segment **7b** was synthesized on a 0.05 mmol scale as described in the general procedure presented in the Methods section. The peptide was cleaved from the solid support and deprotected using a cocktail TFA/H_2O/thiophenol/thioanisole/TIS 87.5/2.5/2.5/2.5/5 v/v/v/v/v (5 mL) during 2 hours, precipitated in 100 mL of ice-cold Et_2O/heptane 1/1 v/v, solubilized in water and lyophilized. Purification of the crude was performed by preparative RP-HPLC using a preparative C18 XBridge BEH300 column (5 µm, 300 Å, 19 × 150 mm, 50 °C, 215 nm, 20 mL min^{-1}, eluent A: 0.1% by vol. of TFA in water, eluent B: 0.1% by vol. of TFA in acetonitrile, 0-25% B in 10 min, then 25-50% B in 45 min). The purified fractions were combined and lyophilized to give the title peptide as a white solid (25.2 mg, 8%).

[6] Desmet, R.; Boidin-Wichlacz, C.; Mhidia, R.; Tasiemski, A.; Agouridas, V.; Melnyk, O. An Iron-Catalyzed Protein Desulfurization Method Reminiscent of Aquatic Chemistry. *Angew. Chem., Int. Ed.* **2023**, *62*, e202302648.

MS (ESI, positive detection mode, Figure S46) m/z = 1744.42 ([M+3H]$^{3+}$), 1308.67 ([M+4H]$^{4+}$), 1047.17 ([M+5H]$^{5+}$), 872.83 ([M+6H]$^{6+}$), 748.25 ([M+7H]$^{7+}$). Calcd. for [M] (average): 5231.18, found: 5230.77.

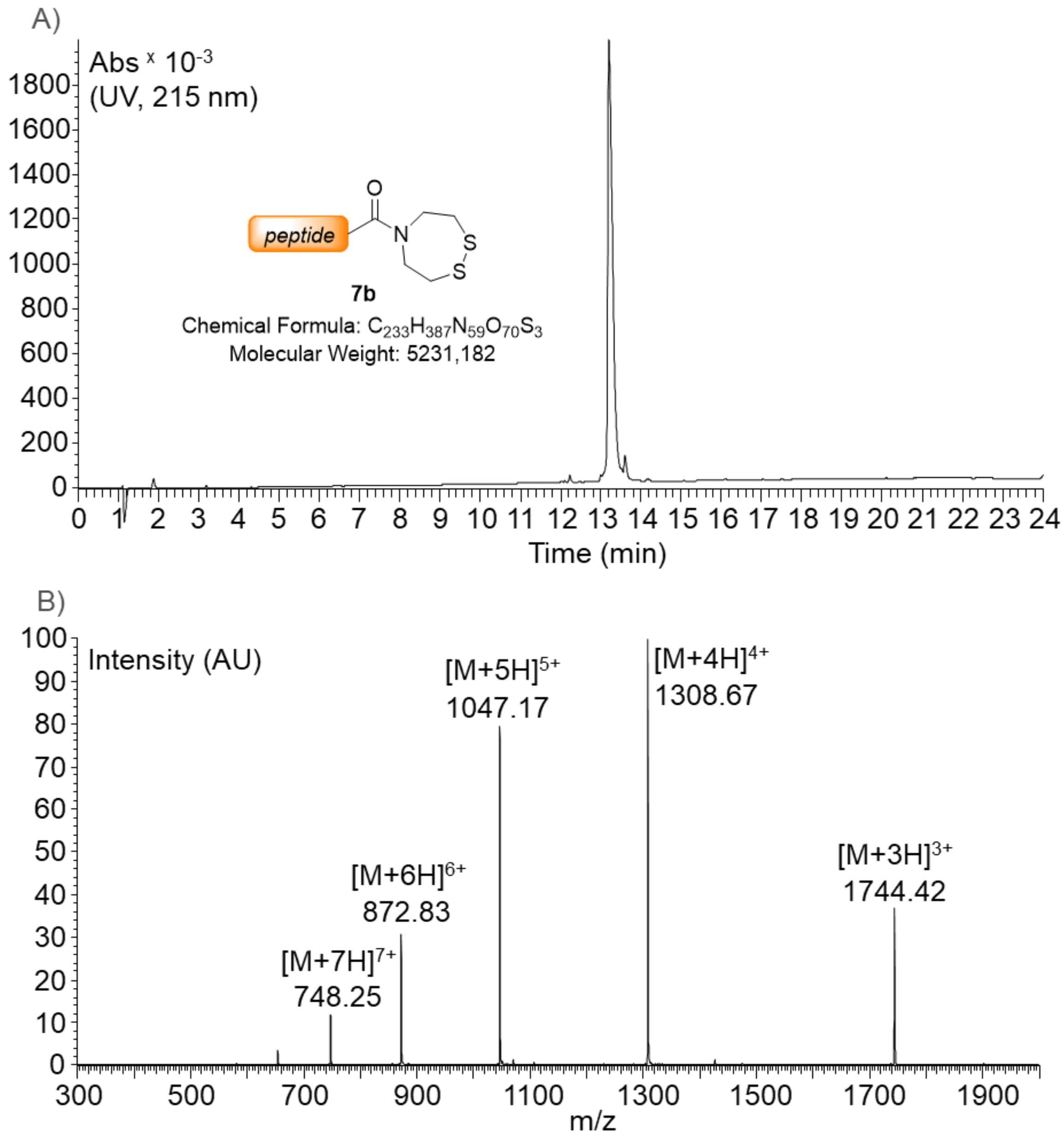

Figure S46. UPLC-MS analysis of **7b** segment. A) LC trace. Eluent A 0.1% TFA in water, eluent B 0.1% TFA in CH_3CN. XBridge BEH C18 (3.5 µm, 300 Å, 2.1 × 150 mm), gradient 0-50% B in 24 min (0.4 mL min^{-1}, detection UV 215 nm). B) MS trace: m/z = 1744.42 ([M+3H]$^{3+}$), 1308.67 ([M+4H]$^{4+}$), 1047.17 ([M+5H]$^{5+}$), 872.83 ([M+6H]$^{6+}$), 748.25 ([M+7H]$^{7+}$). Calcd. for [M] (average): 5231.18, found: 5230.77.

Synthesis and characterization of **8** segment

Segment **8** was synthesized on a 0.1 mmol scale as described in the general procedure presented in the Methods section. The peptide was cleaved from the solid support and deprotected using a cocktail TFA/H_2O/EDT/TIS 90/2.5/2.5/5 v/v/v/v (10 mL) during 50 minutes, precipitated in 200 mL of ice-cold Et_2O/heptane 1/1 v/v, solubilized in water and lyophilized. Purification of the crude was performed by preparative RP-HPLC using a preparative C18 XBridge BEH300 column (5 µm, 300 Å, 19 × 150 mm, 50 °C, 215 nm, 20 mL min^{-1}, eluent A: 0.1% by vol. of TFA in water, eluent B: 0.1% by vol. of TFA in acetonitrile, 0-30% B in 10 min, then 30-55% B in 45 min). The purified fractions were combined and lyophilized to give the title peptide as a white solid (21.9 mg, 5%).

MS (ESI, positive detection mode, Figure S47) m/z = 1311.75 ($[M+3H]^{3+}$), 984.25 ($[M+4H]^{4+}$). Calcd. for [M] (average): 3932.55, found: 3932.63.

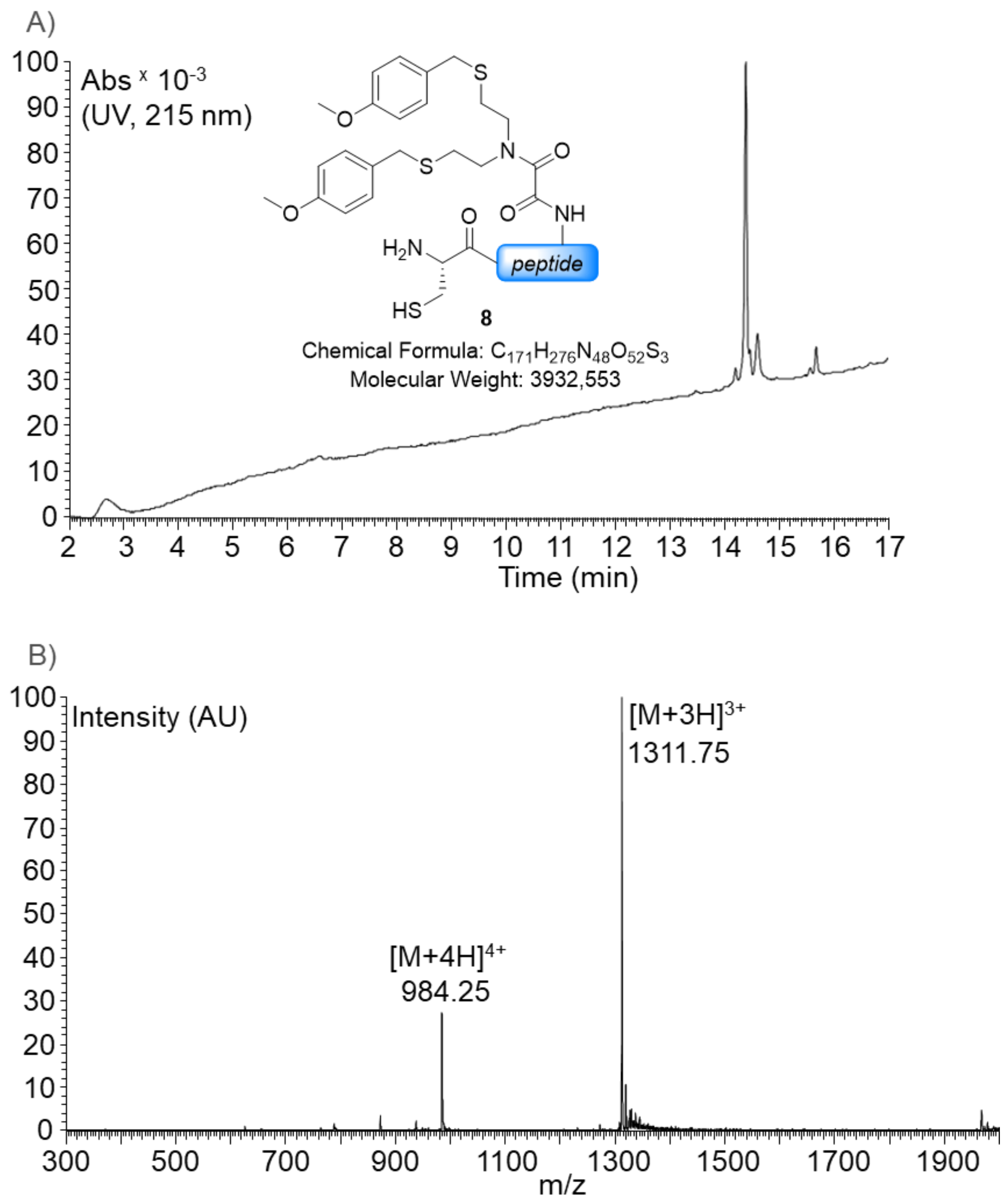

Figure S47. UPLC-MS analysis of **8** segment. A) LC trace. Eluent A 0.1% TFA in water, eluent B 0.1% TFA in CH_3CN. XBridge BEH C18 (3.5 µm, 300 Å, 2.1 × 150 mm), gradient 0-50% B in 15 min (0.4 mL min^{-1}, detection UV 215 nm). B) MS trace: m/z = 1311.75 ($[M+3H]^{3+}$), 984.25 ($[M+4H]^{4+}$). Calcd. for [M] (average): 3932.55, found: 3932.63.

Synthesis and characterization of polypeptide **9b**

To a solution of Gn·HCl (573 mg) in 0.1 M, pH 7.2 phosphate buffer (600 µL) were added TCEP (28.7 mg; 100 µmol; 100 mM) and MPAA (33.6 mg; 200 µmol; 200 mM). The pH of the mixture was adjusted to 5.5 by addition of NaOH 6 M. The **8** segment (3.19 mg; 0.69 µmol; 1.2 mM) was dissolved in 580 µL of the above solution. The resulting mixture (580 µL) was added on the **7b** segment (3.49 mg; 0.58 µmol; 1 mM) and the reaction was stirred overnight at 37°C and under nitrogen atmosphere. The mixture was quenched by addition of glacial acetic acid (64 µL) and the mixture was extracted with diethyl ether (4 × 2 mL). Purification of the crude was performed by preparative RP-HPLC using a preparative C18 XBridge BEH300 column (5 µm, 300 Å, 10 × 250 mm, 50 °C, 215 nm, 6 mL min^{-1}, eluent A: 0.1% by vol. of TFA in water, eluent B: 0.1% by vol. of TFA in acetonitrile, 0-25% B in 10 min, then 25-55% B in 50 min). The purified fractions were combined and lyophilized to give the title peptide as a white solid (3.36 mg, 56%).

MS (ESI, positive detection mode, Figure S48) m/z = 1806.67 ($[M+5H]^{5+}$), 1505.58 ($[M+6H]^{6+}$), 1290.67 ($[M+7H]^{7+}$), 1129.50 ($[M+8H]^{8+}$), 1004.17 ($[M+9H]^{9+}$), 903.83 ($[M+10H]^{10+}$), 821.83 ($[M+11H]^{11+}$). Calcd. for [M] (average): 9028.49, found: 9027.59.

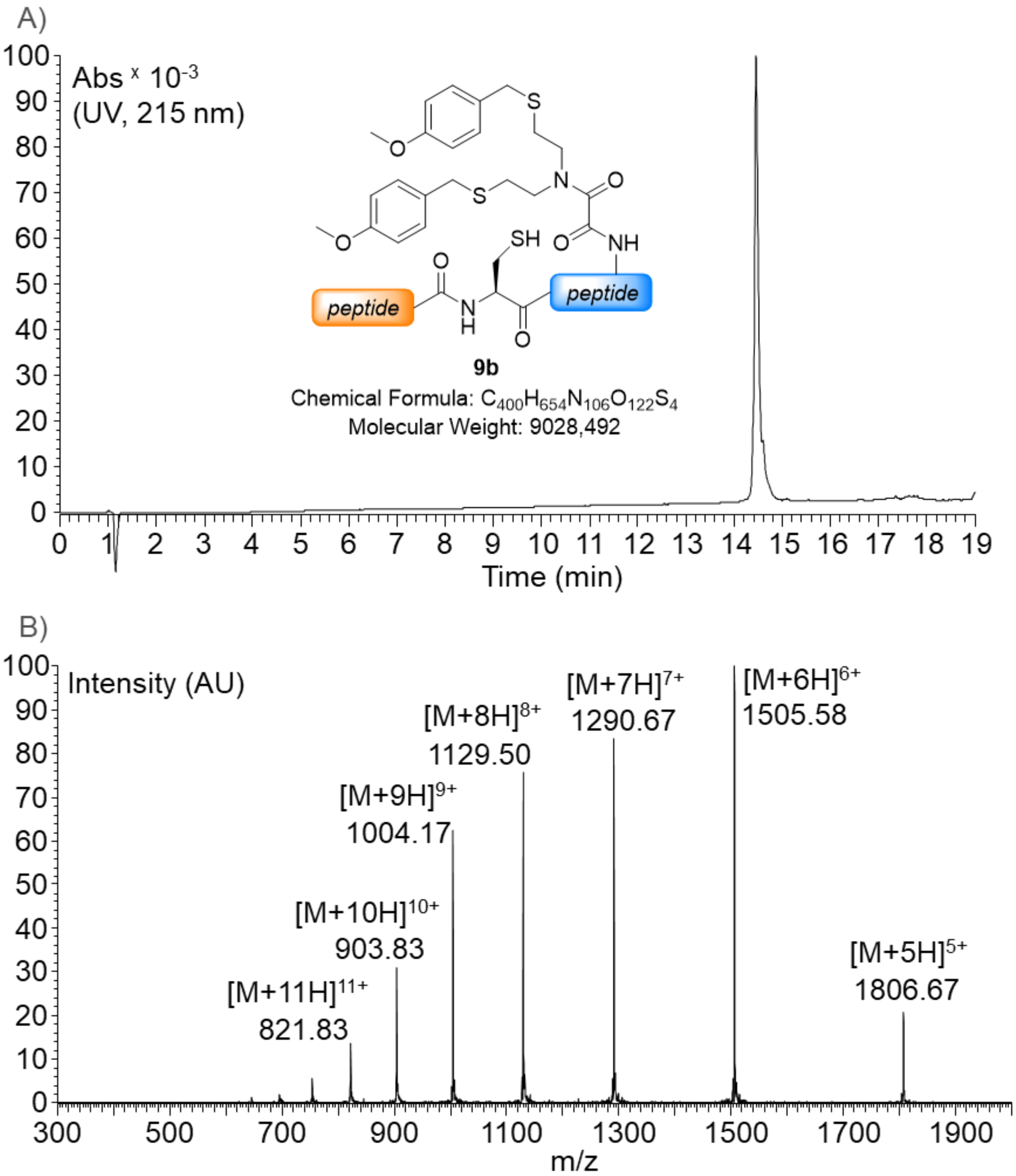

Figure S48. UPLC-MS analysis of polypeptide **9b**. A) LC trace. Eluent A 0.1% TFA in water, eluent B 0.1% TFA in CH_3CN. XBridge BEH C18 (3.5 µm, 300 Å, 2.1 × 150 mm), gradient 0-50% B in 15 min (0.4 mL min^{-1}, detection UV 215 nm). B) MS trace: m/z = 1806.67 ($[M+5H]^{5+}$), 1505.58 ($[M+6H]^{6+}$), 1290.67 ($[M+7H]^{7+}$), 1129.50 ($[M+8H]^{8+}$), 1004.17 ($[M+9H]^{9+}$), 903.83 ($[M+10H]^{10+}$), 821.83 ($[M+11H]^{11+}$). Calcd. for [M] (average): 9028.49, found: 9027.59.

Synthesis and characterization of polypeptide **10b**

To a solution of Gn·HCl (573 mg) in 0.1 M, pH 7.2 phosphate buffer (600 µL) were added TCEP (57.33 mg; 200 µmol; 200 mM), GSH (15.37 mg; 50 µmol; 50 mM) and VA-044 (6.47 mg; 20 µmol; 20 mM). The pH of the mixture was adjusted to 6.5 by addition of NaOH 6 M. The polypeptide **9b** (3.36 mg; 0.32 µmol; 1 mM) was dissolved in 320 µL of the above solution and the reaction was stirred during 5 hours at 37°C. Purification of the crude was performed by preparative RP-HPLC using a preparative C18 XBridge BEH300 column (5 µm, 300 Å, 10 × 250 mm, 50 °C, 215 nm, 6 mL min^{-1}, eluent A: 0.1% by vol. of TFA in water, eluent B: 0.1% by vol. of TFA in acetonitrile, 0-30% B in 10 min, then 30-55% B in 50 min). The purified fractions were combined and lyophilized to give the title peptide as a white solid (1.38 mg, 42%).

MS (ESI, positive detection mode, Figure S49) m/z = 1800.00 ($[M+5H]^{5+}$), 1500.08 ($[M+6H]^{6+}$), 1286.00 ($[M+7H]^{7+}$), 1125.42 ($[M+8H]^{8+}$), 1000.50 ($[M+9H]^{9+}$), 900.50 ($[M+10H]^{10+}$), 819.00 ($[M+11H]^{11+}$). Calcd. for [M] (average): 8996.43, found: 8994.74.

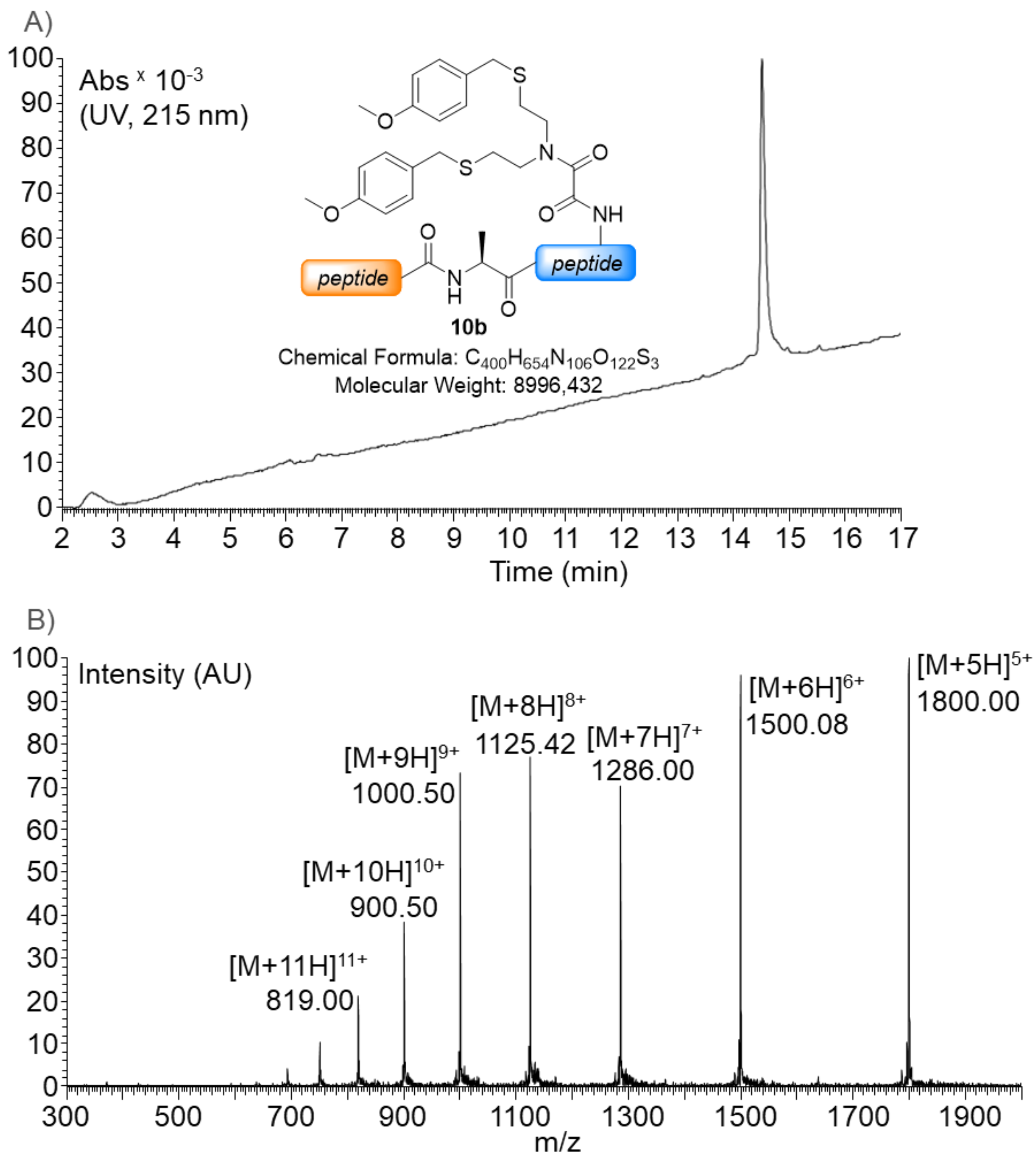

Figure S49. UPLC-MS analysis of polypeptide **10b**. A) LC trace. Eluent A 0.1% TFA in water, eluent B 0.1% TFA in CH_3CN. XBridge BEH C18 (3.5 µm, 300 Å, 2.1 × 150 mm), gradient 0-50% B in 15 min (0.4 mL min^{-1}, detection UV 215 nm). B) MS trace: m/z = 1800.00 ([M+5H]$^{5+}$), 1500.08 ([M+6H]$^{6+}$), 1286.00 ([M+7H]$^{7+}$), 1125.42 ([M+8H]$^{8+}$), 1000.50 ([M+9H]$^{9+}$), 900.50 ([M+10H]$^{10+}$), 819.00 ([M+11H]$^{11+}$). Calcd. for [M] (average): 8996.43, found: 8994.74.

Synthesis and characterization of K48 ^{oxo}SEA-ubiquitin **11b**

To 1 mL of a TFA/thioanisole mixture (95/5) was added trichloromethylsilane (11.5 µL; 100 µmol; 100 mM) and the diphenylsulfoxide (2.0 mg; 10 µmol; 10 mM). Polypeptide **10b** (1.38 mg; 0.13 µmol; 1 mM) was dissolved in 130 µL of the above mixture and the reaction was stirred during 20 minutes at room temperature. The mixture was quenched by addition of 0.2 M, pH 7.2 phosphate buffer (650 µL) and the mixture was extracted with diethyl ether (4 × 1 mL). Purification of the crude was performed by preparative RP-HPLC using a preparative C18 XBridge BEH300 column (5 µm, 300 Å, 10 × 250 mm, 50 °C, 215 nm, 6 mL min^{-1}, eluent A: 0.1% by vol. of TFA in water, eluent B: 0.1% by vol. of TFA in acetonitrile, 0-25% B in 10 min, then 25-50% B in 45 min). The purified fractions were combined and lyophilized to give the title peptide as a white solid (0.33 mg, 25%).

MS (ESI, positive detection mode, Figure S51) m/z = 1751.58 ($[M+5H]^{5+}$), 1459.75 ($[M+6H]^{6+}$), 1251.50 ($[M+7H]^{7+}$), 1095.17 ($[M+8H]^{8+}$), 973.67 ($[M+9H]^{9+}$), 876.42 ($[M+10H]^{10+}$), 796.83 ($[M+11H]^{11+}$), 730.50 ($[M+12H]^{12+}$). Calcd. for [M] (average): 8754.11, found: 8753.70.

Analytical scale reaction was monitored by UPLC-MS through sampling of 2 µL aliquots which were quenched by addition of 0.2 M, pH 7.2 phosphate buffer (20 µL) and then extracted with diethyl ether (3 × 100 µL) before injection (Figure S50).

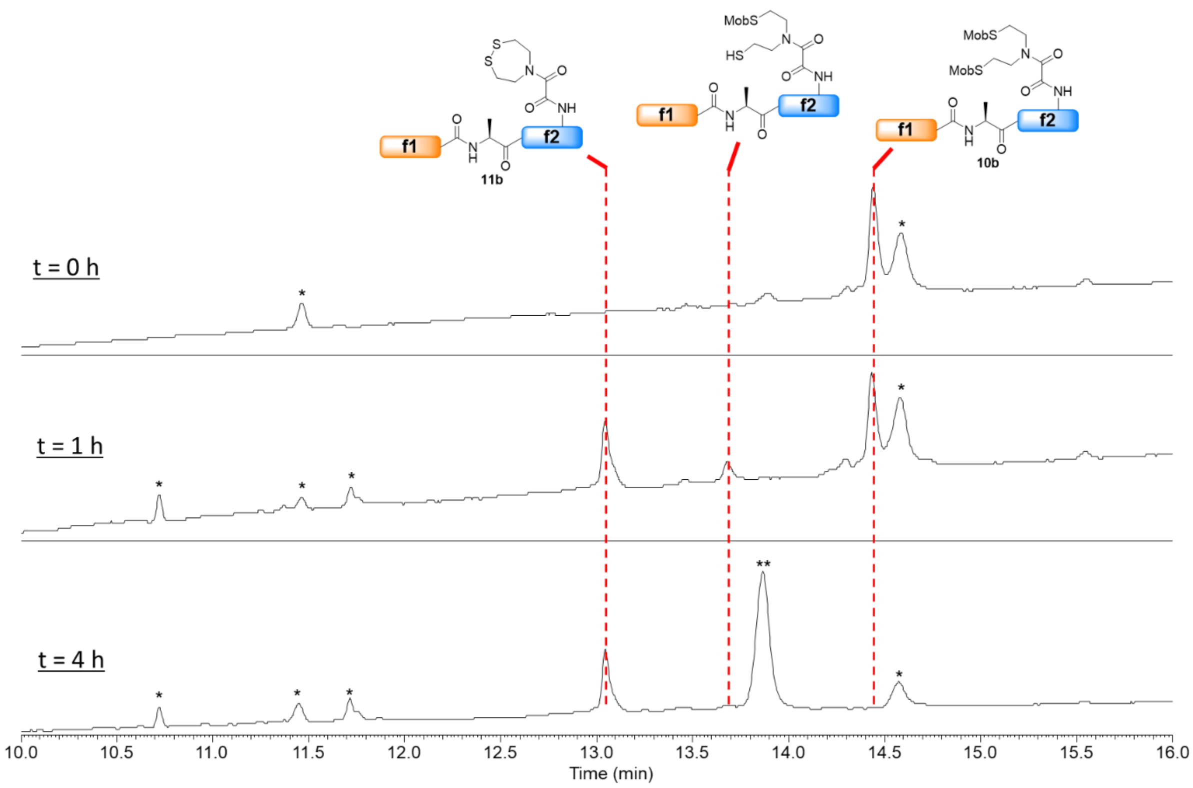

Figure S50. UPLC chromatograms of the monitoring at 215 nm of the Mob cleavage of polypeptide **10b** (polypeptide **10b** 1 mM; diphenylsulfoxide 10 mM; trichloromethylsilane 100 mM; TFA/thioanisole 95/5 v/v) (LC trace. Eluent A 0.1% TFA in water, eluent B 0.1% TFA in CH_3CN. XBridge BEH C18 (3.5 µm, 300 Å, 2.1 × 150 mm), gradient 0-50% B in 15 min (0.4 mL min^{-1}, detection UV 215 nm)). *Non-peptidic impurity. **Thioanisole.

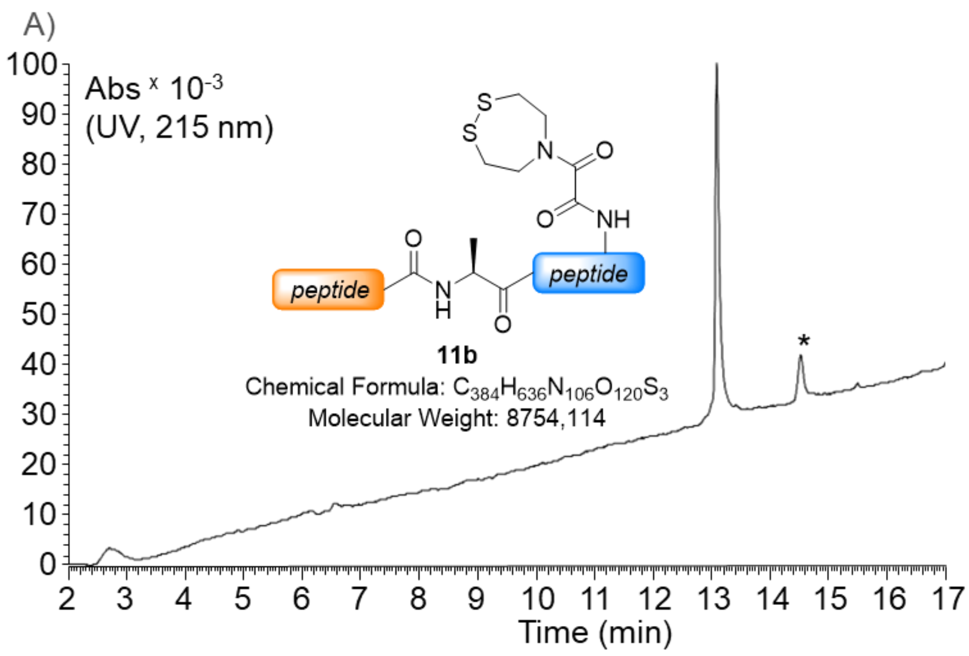
A)
Abs x 10-3
(UV, 215 nm)
peptide
peptide
11b
Chemical Formula: C384H636N106O120S3
Molecular Weight: 8754,114
*
Time (min)

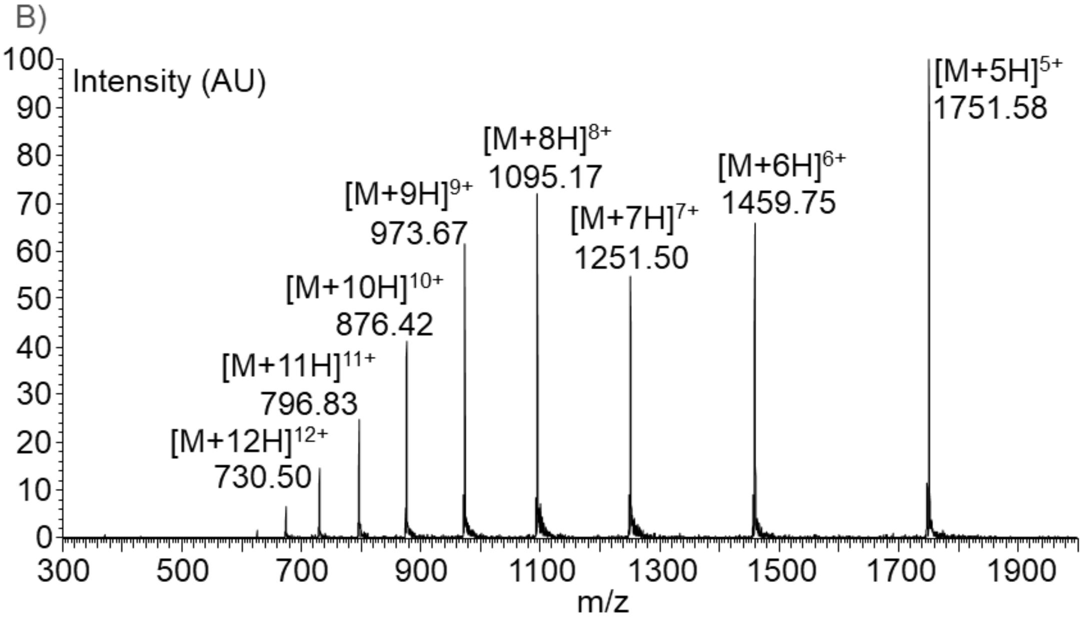
B)
Intensity (AU)
[M+5H]5+
1751.58
[M+8H]8+
1095.17
[M+6H]6+
1459.75
[M+9H]9+
973.67
[M+7H]7+
1251.50
[M+10H]10+
876.42
[M+11H]11+
796.83
[M+12H]12+
730.50
m/z

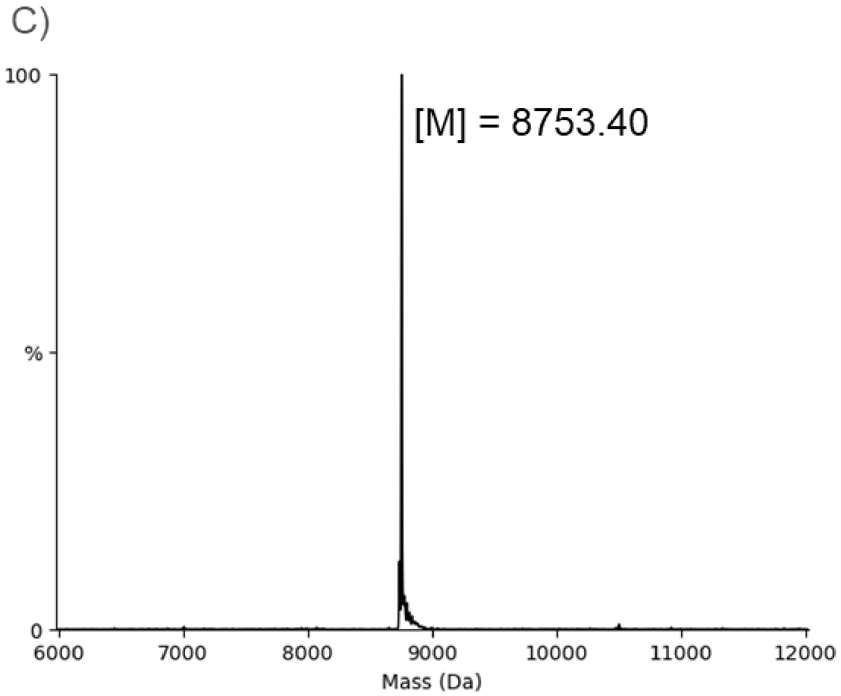

Figure S51. UPLC-MS analysis of K48 ^{oxo}SEA-ubiquitin **11b**. A) LC trace. Eluent A 0.1% TFA in water, eluent B 0.1% TFA in CH_3CN. XBridge BEH C18 (3.5 µm, 300 Å, 2.1 × 150 mm), gradient 0-50% B in 15 min (0.4 mL min^{-1}, detection UV 215 nm). B) MS trace: m/z = 1751.58 ($[M+5H]^{5+}$), 1459.75 ($[M+6H]^{6+}$), 1251.50 ($[M+7H]^{7+}$), 1095.17 ($[M+8H]^{8+}$), 973.67 ($[M+9H]^{9+}$), 876.42 ($[M+10H]^{10+}$), 796.83 ($[M+11H]^{11+}$), 730.50 ($[M+12H]^{12+}$). Calcd. for [M] (average): 8754.11, found: 8753.70. C) Deconvoluted MS spectrum of K48 ^{oxo}SEA-ubiquitin **11b**. *Non-peptidic impurity.

Functional characterization

The K48 ^{oxo}SEA-ubiquitin **11b** was involved in a Fast-SEA ligation reaction with Cys-peptide **12** (CILKEPVHGA-NH_2) according to standard ligation conditions at 50 µM peptide concentration and the reaction was monitored by analytical RP-HPLC (Figure S52).

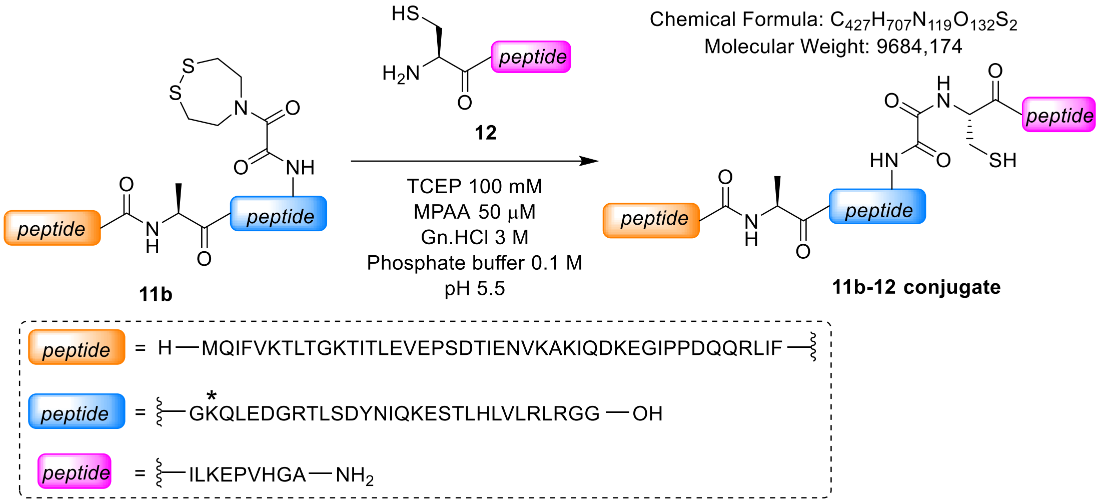

Figure S52. Synthetic scheme for the Fast-SEA ligation reaction between K48 ^{oxo}SEA-ubiquitin **11b** and the Cys-peptide **12** (CILKEPVHGA-NH_2).

Protocol

To a solution of Gn·HCl (573 mg) in 0.1 M, pH 7.2 phosphate buffer (600 µL) were added TCEP (57.4 mg; 200 µmol; 200 mM) and MPAA (16.8 mg; 100 µmol; 100 mM). The pH of the mixture was adjusted to 5.5 by addition of NaOH 6 M. The Cys-peptide **12** (0.21 mg; 0.15 µmol; 150 µM) was dissolved in the above solution. 50 µL of the resulting mixture were added on 50 µL of a 0.1 M pH 5.5 phosphate buffer solution containing the K48 ^{oxo}SEA-ubiquitin **11b** (0.065 mg; 0.0064 µmol; 100 µM) and the reaction was left to proceed at 37°C and under nitrogen atmosphere. The reaction was monitored by RP-HPLC through sampling of 2 µL aliquots which were quenched by addition of acetic acid 10% v/v in water (30 µL) and then extracted with diethyl ether (4 × 100 µL) before injection. Identification of ligation intermediates and products was ensured by UPLC-MS analysis (Figure S53).

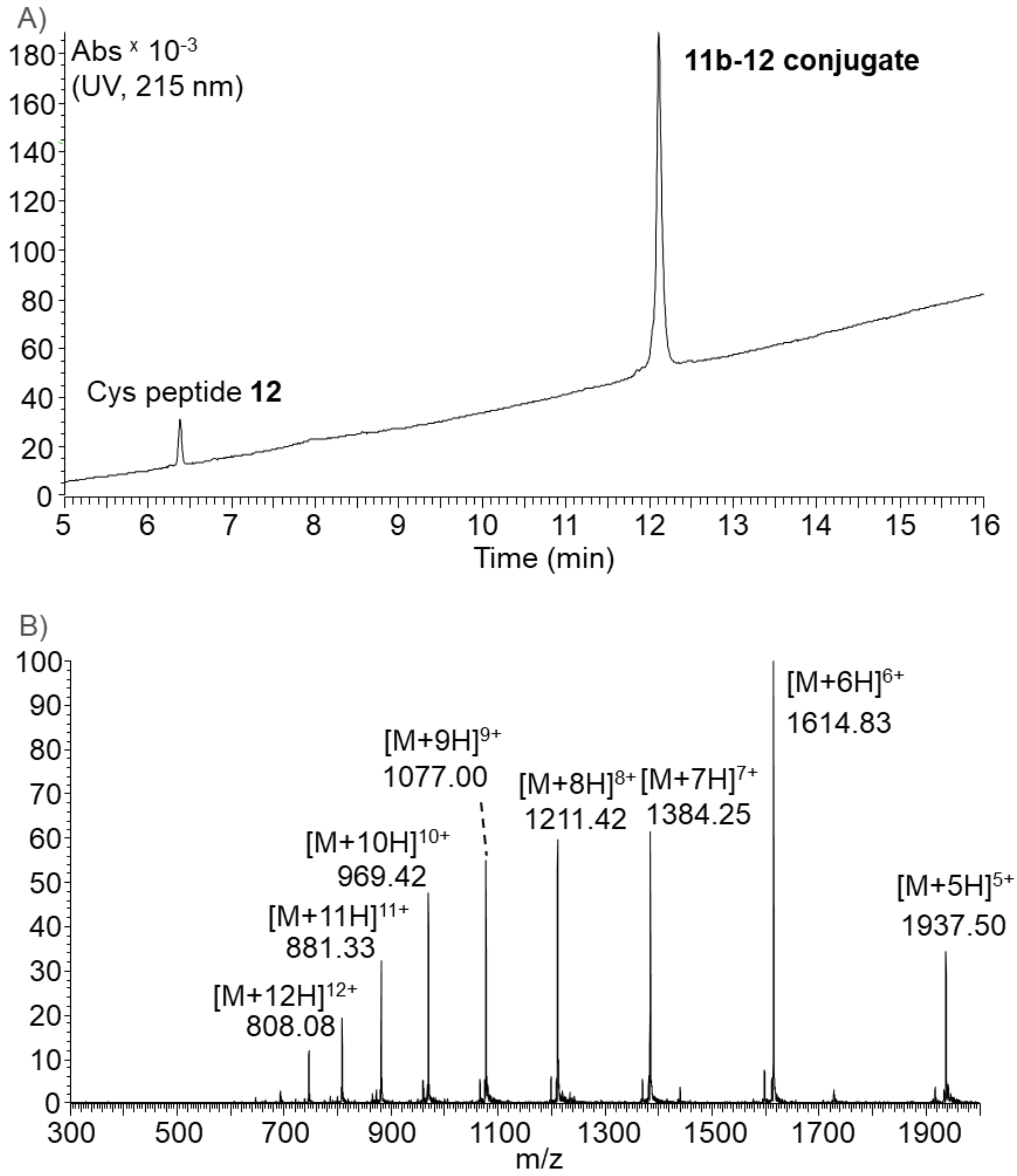

Figure S53. UPLC-MS analysis of the formation of ligation product **11b-12 conjugate**. A) LC trace. Eluent A 0.1% TFA in water, eluent B 0.1% TFA in CH_3CN. XBridge BEH C18 (3.5 µm, 300 Å, 2.1 × 150 mm), gradient 0-50% B in 15 min (0.4 mL min^{-1}, detection UV 215 nm). B) MS trace of compound **11b-12 conjugate**: m/z = 1937.50 ($[M+5H]^{5+}$), 1614.83 ($[M+6H]^{6+}$), 1384.25 ($[M+7H]^{7+}$), 1211.42 ($[M+8H]^{8+}$), 1077.00 ($[M+9H]^{9+}$), 969.42 ($[M+10H]^{10+}$), 881.33 ($[M+11H]^{11+}$), 808.08 ($[M+12H]^{12+}$). Calcd. for [M] (average): 9684.17, found: 9682.87.

Kinetic monitoring

Conversion to ligated product **11b-12 conjugate** was calculated from the UV trace at 215 nm. Conversions were transformed into concentrations of the product based on the starting Ubiquitin(^{oxo}SEA) polypeptide **11b** concentration (Figure S54).

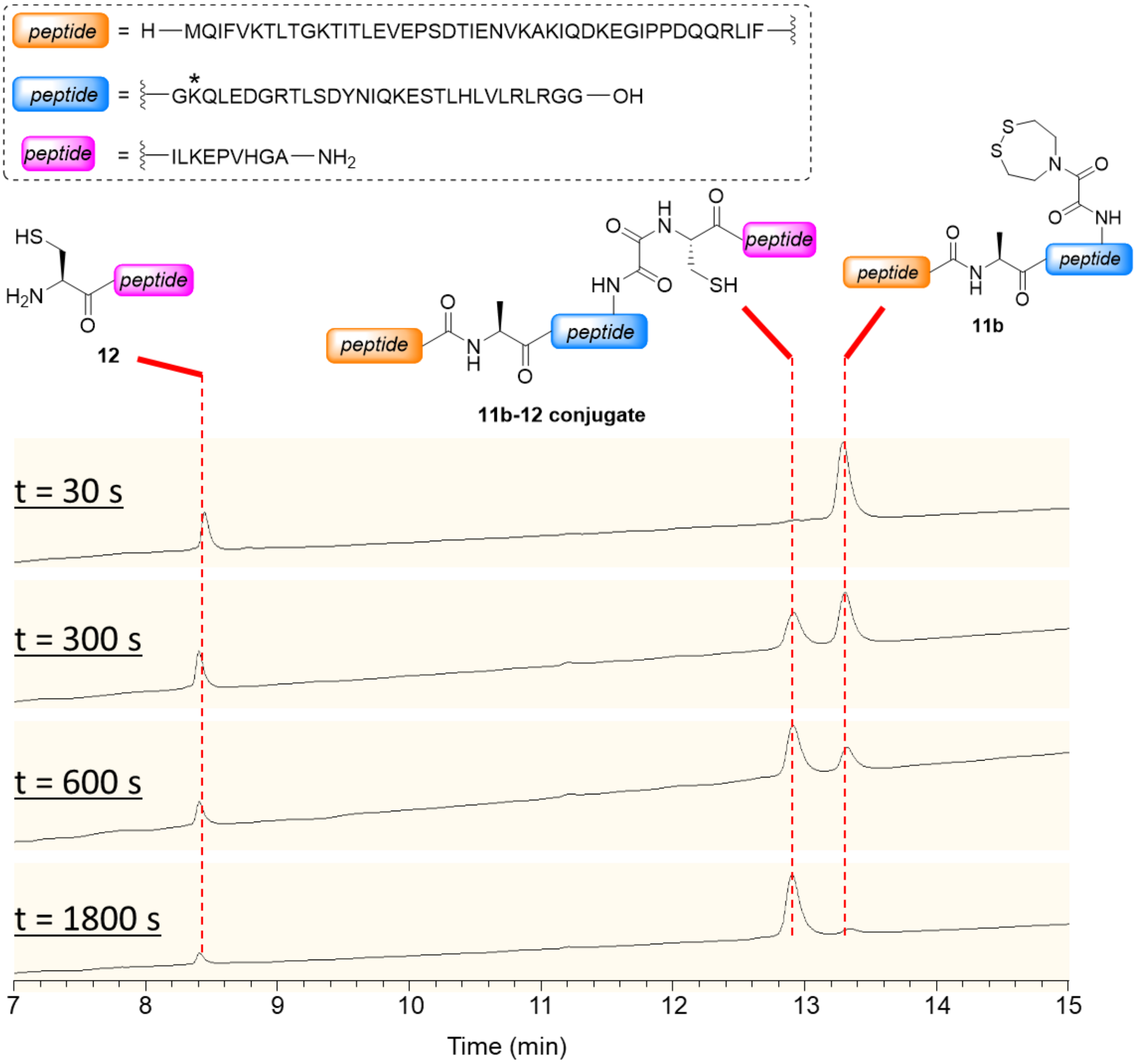

Figure S54. Examples of RP-HPLC chromatograms of the monitoring at 215 nm of the Fast-SEA ligation presented in Figure S52 performed at 50 µM **11b** concentration (Ubiquitin (^{oxo}SEA) **11b** 50 µM; Cys-peptide **12** 75 µM; TCEP 100 mM; MPAA 50 mM; 3 M Gn·HCl in 0.1 M phosphate buffer; pH 5.5; 37 °C) (Eluent A 0.1% TFA in water, eluent B 0.1% TFA in CH_3CN. XBridge BEH C18 (3.5 µm, 300 Å, 4.6 × 150 mm), gradient 0-50% B in 15 min (1.0 mL min^{-1}, detection UV 215 nm).

Proteomic analysis of the conjugate

The identity of the ligation product was confirmed by proteomic analysis of the main peak which was collected following purification by RP-HPLC. After completion of the reaction, the mixture was quenched by addition of glacial acetic acid (10 µL) and then extracted with diethyl ether (4 × 500 µL). Purification of the crude was performed by analytical RP-HPLC using an analytical 300-SB C3 column (3.5 µm, 4.6 × 150 mm, 50 °C, 215 nm, 1 mL min^{-1}, eluent A: 0.1% by vol. of TFA in water, eluent B: 0.1% by vol. of TFA in acetonitrile, 0-25% B in 10 min, then 25-50% B in 45 min). The purified fractions were combined and lyophilized to give **conjugate 11b-12** as a white solid.

Alkylation step. **Conjugate 11b-12** (30 µg) was treated with a solution of dithiothreitol (1 mg mL^{-1}, 15 µL) in a 25 mM ammonium bicarbonate buffer for 15 min. Then a solution of iodoacetamide (10 mg mL^{-1}, 15 µL) in a 25 mM ammonium bicarbonate buffer was added for 15 min. The alkylation step was monitored by UPLC-MS (Figure S55).

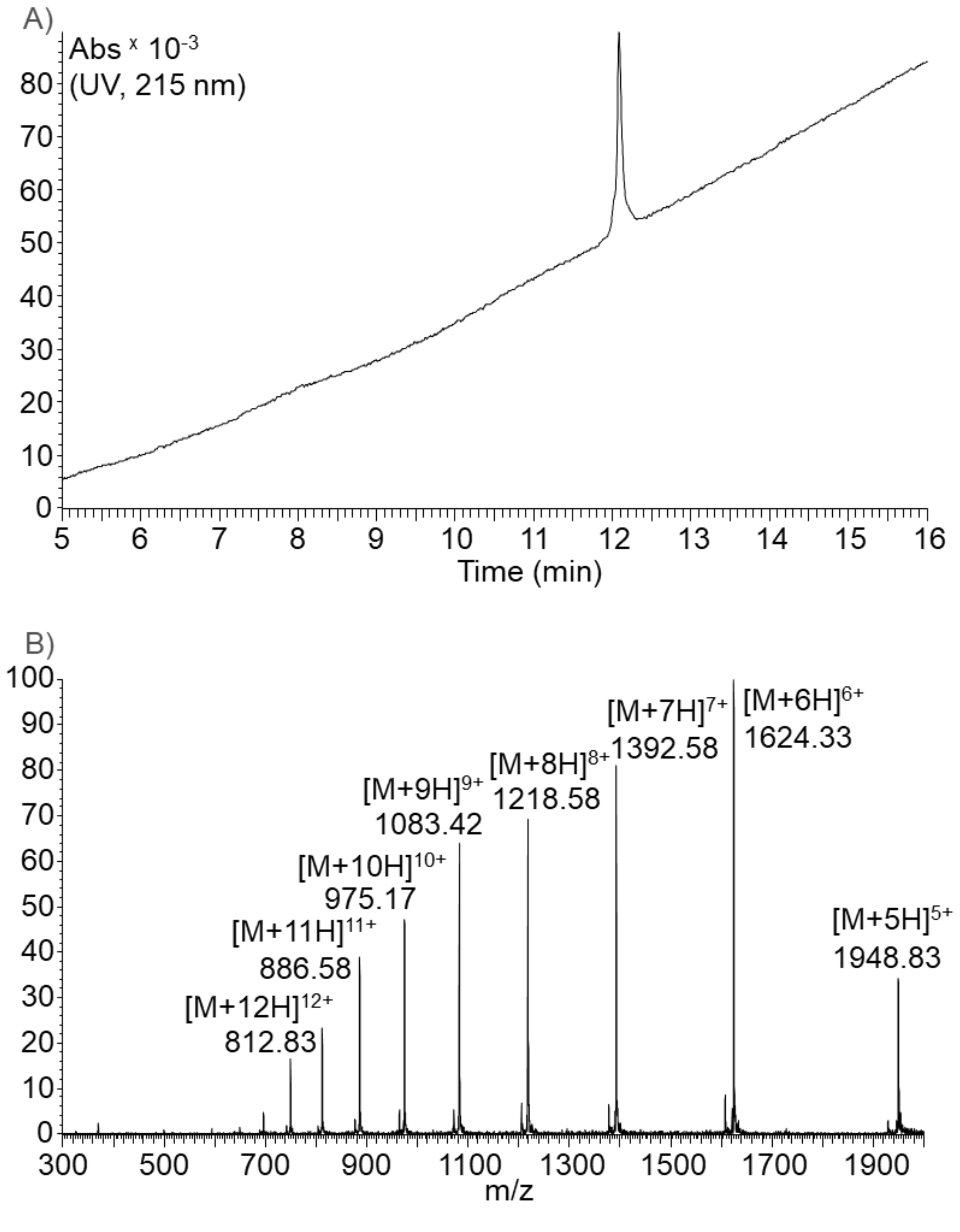

Figure S55. Proteomic analysis of ligation product **conjugate 11b-12**. UPLC-MS analysis of alkylated **conjugate 11b-12**. A) LC trace. Eluent A 0.1% TFA in water, eluent B 0.1% TFA in CH_3CN. XBridge BEH C18 (3.5 µm, 300 Å, 2.1 × 150 mm), gradient 0-50% B in 15 min (0.4 mL min^{-1}, detection UV 215 nm). B) MS trace: m/z = 1948.83 ($[M+5H]^{5+}$), 1624.33 ($[M+6H]^{6+}$), 1392.58 ($[M+7H]^{7+}$), 1218.58 ($[M+8H]^{8+}$), 1083.42 ($[M+9H]^{9+}$), 975.17 ($[M+10H]^{10+}$), 886.58 ($[M+11H]^{11+}$), 812.83 ($[M+12H]^{12+}$). Calcd. for [M] (average): 9741.11, found: 9740.52.

Trypsin digestion. Trypsin (0.1 mg/mL, 0.3 µL) was added to the mixture to cleave the alkylated peptide. The fragments resulting from the enzymatic cleavage were identified by UPLC MS (Figure S56). The fragments obtained confirm the coupling of the cysteinyl peptide **12** with the engineered Ubiquitin protein **11b**. The new junction formed is in fragments F6-F9.

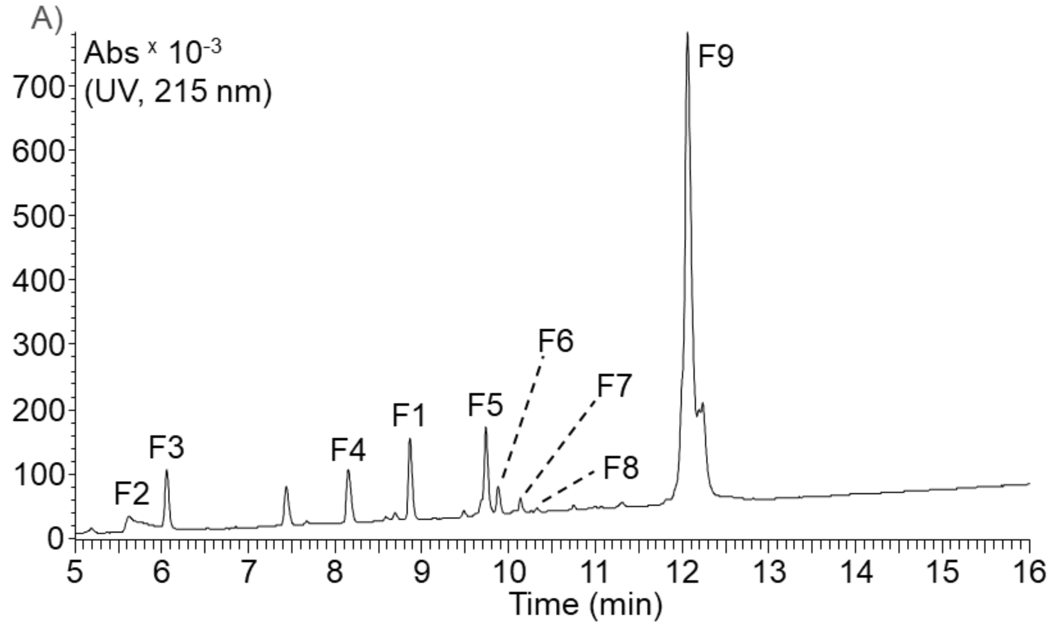

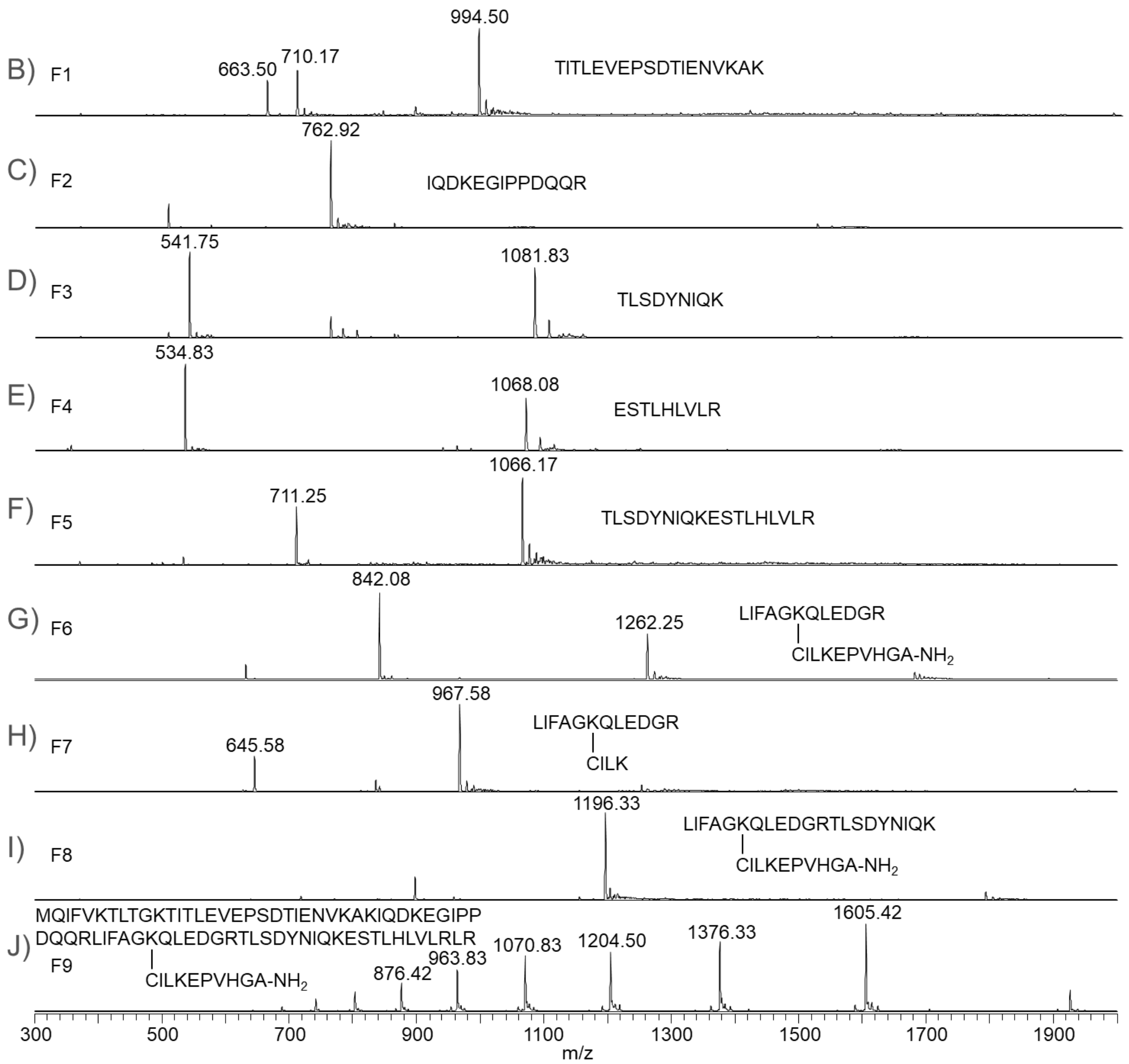

Figure S56. Proteomic analysis of ligation product **conjugate 11b-12**. UPLC-MS analysis of the crude enzymatic lysate after trypsin digestion during 90 min. A) LC trace. Eluent A 0.1% TFA in water, eluent B 0.1% TFA in CH_3CN. XBridge BEH C18 (3.5 µm, 300 Å, 2.1 × 150 mm), gradient 0-50% B in 15 min (0.4 mL min^{-1}, detection UV 215 nm). B-J) MS traces of fractions F1-9 respectively.

9. References

(1) Ollivier, N.; Dheur, J.; Mhidia, R.; Blanpain, A.; Melnyk, O. *Bis*(2-sulfanylethyl)amino Native Peptide Ligation. *Org. Lett.* **2010**, *12*, 5238-5241.

(2) Ollivier, N.; Raibaut, L.; Blanpain, A.; Desmet, R.; Dheur, J.; Mhidia, R.; Boll, E.; Drobecq, H.; Pira, S. L.; Melnyk, O. Tidbits for the synthesis of bis(2-sulfanylethyl)amido (SEA) polystyrene resin, SEA peptides and peptide thioesters. *J. Pept. Sci*. **2014**, *20*, 92-97.

(3) Snella, B.; Grain, B.; Vicogne, J.; Capet, F.; Wiltschi, B.; Melnyk, O.; Agouridas, V. Fast Protein Modification in the Nanomolar Concentration Range Using an Oxalyl Amide as Latent Thioester. *Angew. Chem., Int. Ed.* **2022**, *61*, e202204992.

(5) Ste.Marie, E. J.; Hondal, R. J. Reduction of cysteine *S*-protecting groups by triisopropylsilane. *J. Pept. Sci*. **2018**, *24*, e3130.

(6) Desmet, R.; Boidin-Wichlacz, C.; Mhidia, R.; Tasiemski, A.; Agouridas, V.; Melnyk, O. An Iron-Catalyzed Protein Desulfurization Method Reminiscent of Aquatic Chemistry. *Angew. Chem., Int. Ed.* **2023**, *62*, e202302648.
